# Disparities in mortality among 25–44-year-olds in England: a longitudinal, population-based study

**DOI:** 10.1016/S2468-2667(18)30177-4

**Published:** 2018-10-31

**Authors:** Evangelos Kontopantelis, Iain Buchan, Roger T Webb, Darren M Ashcroft, Mamas A Mamas, Tim Doran

**Affiliations:** a Division of Informatics, Imaging and Data Sciences, Faculty of Biology, Medicine and Health, University of Manchester, Manchester, UK; b Division of Psychology and Mental Health, Faculty of Biology, Medicine and Health, University of Manchester, Manchester, UK; c Division of Pharmacy and Optometry, Faculty of Biology, Medicine and Health, University of Manchester, Manchester, UK; d Public Health and Clinical Informatics, Department of Public Health and Policy, Faculty of Health and Life Sciences, University of Liverpool, Liverpool, UK; e National Institute for Health Research, School for Primary Care Research, University of Manchester, Manchester, UK; f NIHR Greater Manchester Patient Safety Translational Research Centre, University of Manchester, Manchester, UK; g Manchester Academic Health Sciences Centre, Manchester, UK; h Keele Cardiovascular Research Group, Centre for Prognosis Research, Institute of Primary Care and Health Sciences, Keele University, UK; i Department of Health Sciences, Seebohm Rowntree Building, University of York, York, UK

## Abstract

**Background:**

Since the mid-1990s, excess mortality has increased markedly for adults aged 25–44 years in the north compared with the south of England. We examined the underlying causes of this excess mortality and the contribution of socioeconomic deprivation.

**Methods:**

Mortality data from the Office of National Statistics for adults aged 25–44 years were aggregated and compared between England's five most northern versus five most southern government office regions between Jan 1, 1981, and Dec 31, 2016. Poisson regression models, adjusted for age and sex, were used to quantify excess mortality in the north compared with the south by underlying cause of death (accidents, alcohol related, cardiovascular disease and diabetes, drug related, suicide, cancer, and other causes). The role of socioeconomic deprivation, as measured by the 2015 Index of Multiple Deprivation, in explaining the excess and regional variability was also explored.

**Findings:**

A mortality divide between the north and south appeared in the mid-1990s and rapidly expanded thereafter for deaths attributed to accidents, alcohol misuse, and drug misuse. In the 2014–16 period, the northern excess was incidence rate ratio (IRR) 1·47 (95% CI 1·39–1·54) for cardiovascular reasons, 2·09 (1·94–2·25) for alcohol misuse, and 1·60 (1·51–1·70) for drug misuse, across both men and women aged 25–44 years. National mortality rates for cardiovascular deaths declined over the study period but a longstanding gap between north and south persisted (from 33·3 [95% CI 31·8–34·8] in 1981 to 15·0 [14·0–15·9] in 2016 in the north *vs* from 23·5 [22·3–24·8] to 9·9 [9·2–10·5] in the south). Between 2014 and 2016, estimated excess numbers of death in the north versus the south for those aged 25–44 years were 1881 (95% CI 726–2627) for women and 3530 (2216–4511) for men. Socioeconomic deprivation explained up to two-thirds of the excess mortality in the north (IRR for northern effect reduced from 1·15 [95% CI 1·14–1·15; unadjusted] to 1·05 [1·04–1·05; adjusted for Index of Multiple Deprivation]). By 2016, in addition to the persistent north–south gap, mortality rates in London were lower than in all other regions, with IRRs ranging from IRR 1·13 (95% CI 1·12–1·15) for the East England to 1·22 (1·20–1·24) for the North East, even after adjusting for deprivation.

**Interpretation:**

Sharp relative rises in deaths from cardiovascular reasons, alcohol misuse and drug misuse in the north compared with the south seem to have created new health divisions between England's regions. This gap might be due to exacerbation of existing social and health inequalities that have been experienced for many years. These divisions might suggest increasing psychological distress, despair, and risk taking among young and middle-aged adults, particularly outside of London.

**Funding:**

Medical Research Council and Wellcome Trust.

## Introduction

Historical economic, political, and cultural patterns in England have created enduring inequalities in health outcomes between different social groups and communities. The most striking of these patterns is known as the north–south divide, a persistent disparity in wealth and health between the populations living in the north and south of the country. Worse health outcomes in the north reflect higher mean levels of deprivation,^1^ but deprived neighbourhoods in the north also have worse health outcomes than similarly deprived neighbourhoods in the south,^2^ and people with the same socioeconomic status tend to have poorer health if they live in the north than if they live in the south.^3^ The possible causes of these regional disparities in England are complex, and are likely to include environmental, occupational, migratory, epigenetic, and lifestyle factors, in addition to long-term structural imbalances of resources and investment in the south, particularly in and around London.^4^

In England, the north–south divide in health is most evident in patterns of mortality. Although national mortality has fallen over time in England, and the leading causes of death have transitioned from infectious diseases and occupational injuries to long-term conditions, the risk of premature death (aged <75 years) has remained consistently higher in the north than in the south.^5^ Since the 1960s, the proportion of people dying prematurely has been at least 10% higher in the north than the south,^6^ and the gap has persisted, despite high-profile inquiries^7^ and national programmes intended to address health inequalities.^8^ Such national variability is not uncommon. In the USA, those states with the lowest healthy life expectancies are almost exclusively concentrated in the south.^9^ In Spain, disability-free life expectancy at birth and at 65 years is substantially lower in the south than the north, with the difference at least partially attributed to education, unemployment, and smoking.^10^ A north–south mortality divide also exists in France, with most départements in the south having some of the highest life expectancies for both men and women, and those in the north consistently having the lowest.^11^ Italy has one of the highest life expectancies in the world, but a more modest north–south gap also exists there, with both men and women in the north living longer on average than those in the south.^12^

**Research in context**

**Evidence before this study**

In England, profound regional differences exist in standards of living, both within and between regions. A stark contrast exists between the north and the south of the country, termed the north–south divide. Cultural, economic, and social differences between the north and the south of England have existed for centuries. From 1965 to 2008, the chances of dying early (<75 years) were a fifth higher in the north of England than in the south, while England's overall mortality fell by around 50% in men and 40% in women. From the mid-1990s, the north of England experienced a profound rise in premature mortality in adults aged 25–44 years compared with the south. This rise was preceded by at least three decades of narrowing inequalities in mortality between the regions.

**Added value of this study**

Excess mortality in the north for ages 25–44 years was primarily attributed to alcohol, cardiovascular and drug-related deaths, with suicides, cancer and accidents also playing important roles. Measured deprivation explained up to two-thirds of excess mortality in the north, with the remaining one-third likely attributable to unmeasured aspects of deprivation or cultural differences. Most northern regions have higher mortality than in most southern regions for young and middle-aged adults, but mortality in both north and south are at least 13% higher than in London. All of these are findings from this study are important to address previously unaswered questions such as to what extent are causes of deaths socioeconomically driven and is there additional regional variability?

**Implications of all the available evidence**

Socioeconomic inequalities are at the heart of the north–south divide in mortality for people aged 25–44 years. Major structural change is needed to counter England's centralist tradition around London, which is driving important public health inequalities for people aged 25–44 years, especially men.

Since the mid-1990s in England, there has been a marked trend of increasing excess mortality in the north for those aged 25–44 years, which, until recently, was concealed in the mortality statistics for the wider English population.^5^ This north–south divide first began to emerge in the mid-1990s, and the mortality was 40% higher in the north than in the south by 2015.^5^ Deaths in this age group are most commonly attributable to cardiovascular disease; accidents; and suicide, poisoning, and the sequelae of drug and alcohol misuse. However, how much each of these factors contributed to the recent rapid rise in mortality in young adults living in the north of England compared with the south is not known. We identified and explored the reasons behind these increases in excess mortality in the north, and examined how much of this excess mortality could be explained by socioeconomic deprivation.

## Methods

### Study design and procedures

Information on data sources and methodological and population details are provided in the appendix (pp 1–4).^13^

Mortality statistics were based on information recorded when deaths were certified and registered. Most deaths are certified by a medical practitioner, using the Medical Certificate of Cause of Death. This certificate is taken to a registrar by an informant—usually a near relative of the deceased. Deaths should be registered within 5 days of the date of death, although there are a number of situations when the registration of a death will be delayed. In cases of a coroner investigation, the coroner sends information to the registrar and this is used to register the death. Dates recorded varied across cause of death.

Person-level consent and study registration was not required because public administrative data were used for this population-level study.

### Statistical analysis

Deaths and population data were aggregated into the ten government office regions, which were then categorised as either north (North East, North West, Yorkshire and the Humber, East Midlands, and West Midlands) or south (East of England, South Central, South West, South East, and London).^5^, ^6^

Cause-specific mortality rates were calculated from death registrations with the information used in the Office of National Statistics (ONS) mortality statistics and came from one of four sources: (1) details supplied by the doctor when certifying a death—for example, whether the body was seen after death, cause of death, when the deceased was last seen alive, and whether a post mortem was carried out; (2) details supplied by the informant to the registrar—for example, occupation of deceased, sex, usual address, date and place of birth, marital status, date of death, place of death; (3) details supplied by a coroner to the registrar following investigation—for example, cause of death (following post mortem), place of accident (following inquest). In the case of deaths certified after inquest, the coroner supplies the registrar with all the particulars that would have been supplied by the informant; and (4) details derived from information supplied by one of the above—for example, age of deceased is derived from date of birth and coded cause of death.^13^ Cause-specific mortality rates were also calculated from mid-year population estimates and examined by age group (25–29 years, 30–34 years, 35–39 years, and 40–44 years), sex, and north–south dichotomy over time. Categories of death used in this study were consistent with those designated by the ONS. For each of the groups by cause of death (accidents, alcohol related, female breast cancer, other cancers, cardiovascular disease and diabetes, drug related, suicide, and all other causes), we used a Poisson regression model to calculate excess mortality in the north in each calendar year, for each sex, adjusted for the age distribution of the population in the two respective areas.

The estimated incidence rate ratios (IRRs), which can be interpreted as the percentage of population-adjusted excess mortality in the north compared with the south, are presented in sex-specific contour plots, by sex, to visualise changes in excess mortality according to age groups as a time-dependent variable. Using the IRR estimates from these models and the population structure, we also estimated the number of adjusted excess deaths in the north by underlying cause (ie, the disease or injury that initiated the train of events directly leading to death or the circumstances of the accident or violence that produced the fatal injury) as recorded on the death certificate, cross-sectionally for the last 3 years of the study period (2014–16) using the following equation:

$$D_{N}*(1-1/IRR)$$

Where *D*_N_ is the number of deaths in the north. Standardised mortality rates per 100 000 people, by age and sex and with the total respective population of the study period as the reference (from 1981 or 1993, depending on the underlying cause), were calculated for each calendar year and by area and plotted over time.

To evaluate the role of deprivation in excess mortality in the north, we fitted Poisson regression models, with the unit of analysis being a low geography level (lower super output area level; approximately 1500 people, appendix p 1). Deprivation was measured by the 2015 Index of Multiple Deprivation (IMD) with a scale of 0–100, transformed to be normally distributed, where a higher value means higher deprivation; we report the changed association of region when controlling for deprivation.^14^ The outcome was all-cause mortality in 2016 and the two covariates of interest were area and deprivation; all models were adjusted for the age–sex population structure. The first model included only area (north *vs* south), a second one included area and overall deprivation, and a third included the ten English regions and the overall deprivation. A fourth model included area and the IMD domains for employment, housing, crime, and environment chosen to focus on socioeconomic aspects and avoid collinearity; the fifth and final model included the ten English regions and these four IMD domains.

To highlight contemporary patterns in numbers of deaths, we focused on deaths between 2014 and 2016. Additionally, we present time trends for the whole study period from 1981 (1993 for drug poisonings) to 2016. Stata version 15 was used throughout. Scripts for all analyses are provided in the appendix.

### Role of the funding source

The funders of the study had no role in study design, data collection, data analysis, data interpretation, or writing of the report. The corresponding author had full access to all the data in the study and had final responsibility for the decision to submit for publication.

## Results

From Jan 1, 2014, to Dec 31, 2016, 22 530 deaths occurred in men and 13 179 in women aged 25–44 years in England. The population was larger in the south for all four age subgroups, with an annual mean of 4 081 133 women and 4 070 223 men aged 25–44 years, compared with 3 229 532 women and 3 211 064 men in the north. We used official ONS data based on extrapolations and over 3 years.

**Figure 1:**
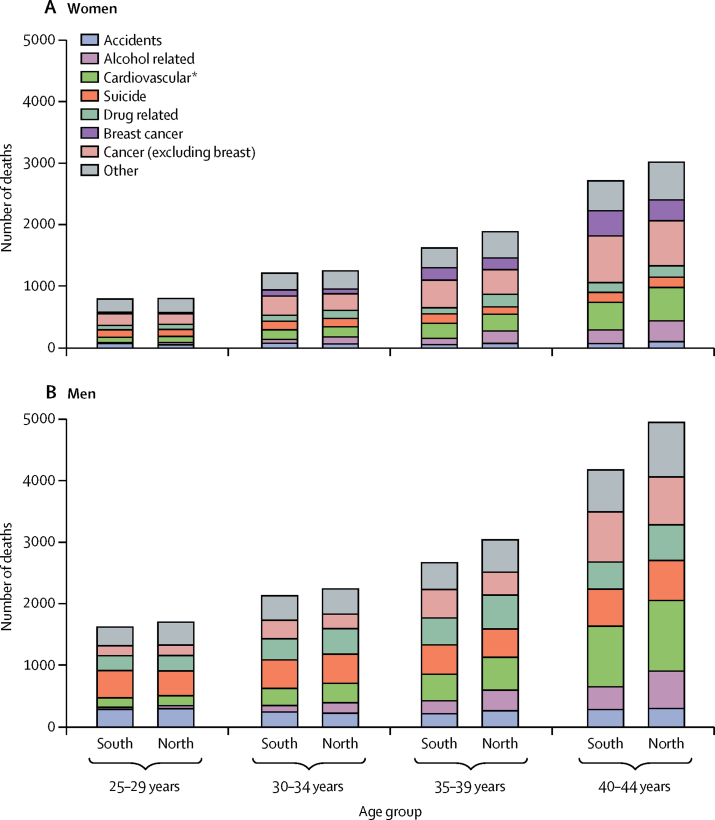
Numbers of deaths attributed to specific underlying causes between 2014 and 2016, for women (A) and men (B) *Includes diabetes and obesity, with most deaths attributed to cardiovascular disease. For men and women, 94·3% of deaths were due to cardiovascular reasons, 3·8% were from diabetes, and 1·9% were from obesity.

Deaths were substantially higher for men compared with women in all four age subgroups: 3 320 versus 1 572 for the 25–29 years group, 4 370 versus 2 431 for the 30–34 years group, 5 706 versus 3 466 for the 35–39 years group, and 9 134 versus 5 710 for the 40–44 years group (figure 1). Accidents (571 [17·2%] of 3320 deaths for the 25–29 years group, 455 [10·4%] of 4 370 for the 30–34 years group, 466 [8·2%] of 5706 for the 35–39 years group, and 573 [6·3%] of 9134 for the 40–44 years group), suicides (850 [25·6%] of 3320, 939 [21·5%] of 4370, 940 [16·5%] of 5706, and 1254 [13·7%] of 9134, respectively) and drug misuse (490 [14·8%] of 3320, 760 [17·4%] of 4370, 991 [17·4%] of 5706, and 1021 [11·2%] of 9134, respectively) were the most common causes of deaths in all age groups in men (figure 1). Deaths attributed to cardiovascular disease in men increased with age: (318 [9·6%] of 3320 deaths for the 25–29 years group, 594 [13·6%] of 4370 for the 30–34 years group, 966 [16·9%] of 5706 for the 35–39 years group, and 2137 [23·4%] of 9134 for the 40–44 years group; figure 1).

The pattern was similar for women, with a smaller contribution from suicides (234 [14·9%] of 1572 deaths for the 25–29 years group, 272 [11·2%] of 2431 for the 30–34 years group, 268 [7·7%] of 3466 for the 35–39 years group, and 331 [5·8%] of 5710 for the 40–44 years group) and accidents (111 [7·1%] of 1572 deaths for the 25–29 years group, 132 [5·4%] of 2 431 for the 30–34 years group, 116 [3·3%] of 3466 for the 35–39 years group, and 164 [2·9%] of 5710 for the 40–44 years group).

Overall in the 2014–16 period, the northern excess was 1·47 (95% CI 1·39–1·54) for cardiovascular reasons, 2·09 (1·94–2·25) for alcohol misuse, and 1·60 (1·51–1·70) for drug misuse, across both men and women aged 25–44 years. The number of people who died between Jan 1, 2014, and Dec 31, 2016, was higher in the north than the south (18 825 *vs* 16 884), despite the larger population in the south (figure 1). A greater proportion of all deaths in people aged 25–44 years were not included in one of the seven main groups of interest for women compared with men, and consequently were included in the other category (4020 [17·8%] men and 2827 [21·5%] women). The relative contributions of each group of underlying causes are presented in the appendix (p 7).

Changes in excess mortality in the north compared with the south from 1981 (1993 for drug-related deaths) to 2016 are presented in contour plots for each group of underlying causes for all ages (appendix pp 10–18). At the start of the study period in 1981, all-cause mortality did not differ substantially between the north and the south in people aged 25–44 years (appendix p 10). In some years during the early-to-mid-1990s, there was no difference in mortality in men between the north and the south. From the mid-1990s, excess mortality in the north increased for both sexes, with an IRR of 1·52 (95% CI 1·46–1·58) by the end of the study period in 2016 in the 35–39 years group. Accidental, alcohol-related, and drug-related deaths were associated with the highest excess mortality in the north over the whole study period (appendix pp 11, 12, 15), and for all causes excess mortality in the north increased over time (appendix pp 10–18). Variation between sexes was small, although there was higher excess mortality from drug misuse for women and higher excess suicide risk for men (appendix pp 14, 15).

**Figure 2:**
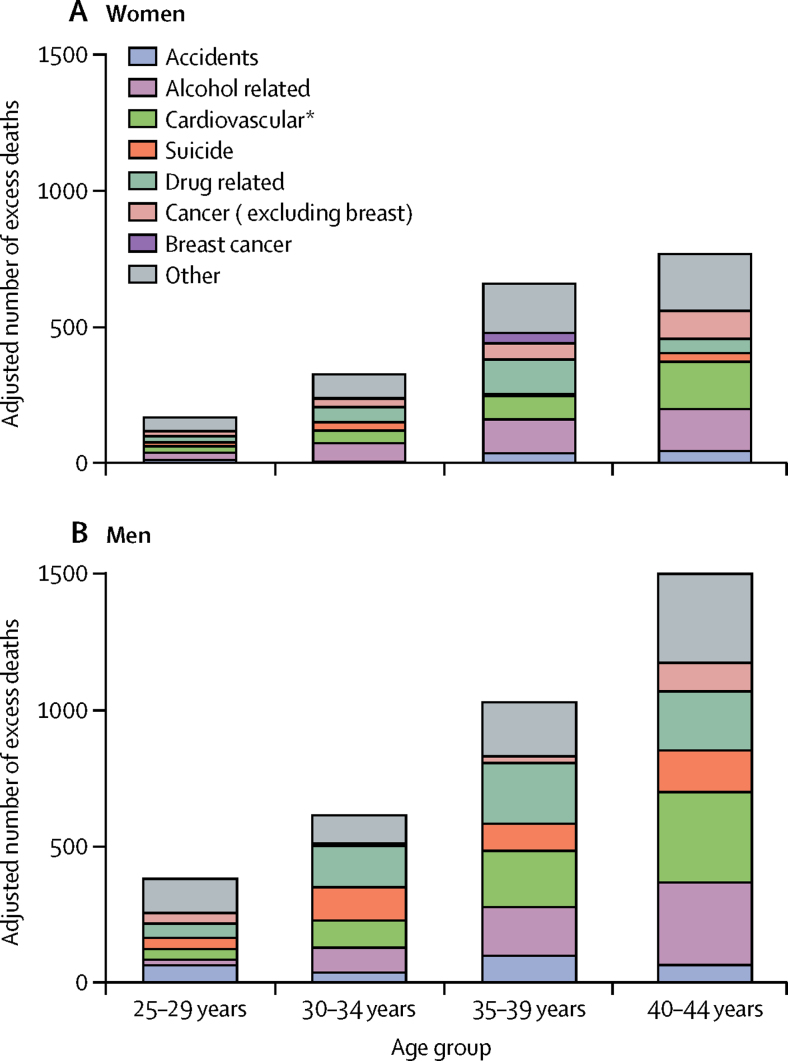
Numbers of excess deaths in the north compared with the south of England attributed to specific underlying causes between 2014 and 2016, for women (A) and men (B) In women, there were two small negative estimates (appendix p 8). *Cardiovascular includes diabetes and obesity, with most deaths attributed to cardiovascular disease. For men and women, 94·3% of deaths were due to cardiovascular reasons, 3·8% were from diabetes, and 1·9% were from obesity.

Between 2014 and 2016, the number of excess deaths in the north compared with the south among women was 1881 (95% CI 726 to 2627) in total with 153 (−95 to 282) for the 25–29 years group, 318 (55 to 478) for the 30–34 years group, 650 (377 to 835) for the 35–39 years group, and 760 (389 to 1032) for the 40–44 years groups; for men, the figure was 3530 (2216 to 4511) in total with the respective figures of 382 (103 to 572), 615 (315 to 832), 1031 (711 to 1272), and 1502 (1087 to 1835; figure 2; appendix p 8). For women, the major contributing causes, excluding other causes, were alcohol related (369 [19·6%] of 1881 deaths), drug related (258 [13·7%] of 1881), cardiovascular disease (330 [17·5%] of 1881), and all-causes cancer (246 [13·1%] of 1881). For men, the patterns were similar, but with some key differences. First, suicide was also a major contributor for all age groups combined (417 [11·8%] of 3530 deaths) in men. Second, the contribution of cancer was substantially smaller in men than in women (178 [5·0%] of 3530 deaths). Third, accidents contributed more towards deaths in men (264 [7·5%] of 3530 deaths) than women and this underlying cause was the largest specific contributor to excess deaths for the 25–29 years group (63 [16·5%] of 382 deaths). The proportional contribution of each underlying cause is presented in the appendix (pp 8, 9, 19). A large proportion of excess deaths in the north across all age groups was attributed to causes in the other category (526 [28·0%] of 1881 for women and 752 [21·3%] of 3530 for men). IRRs by age group and underlying cause are reported in the appendix (p 9).

**Figure 3:**
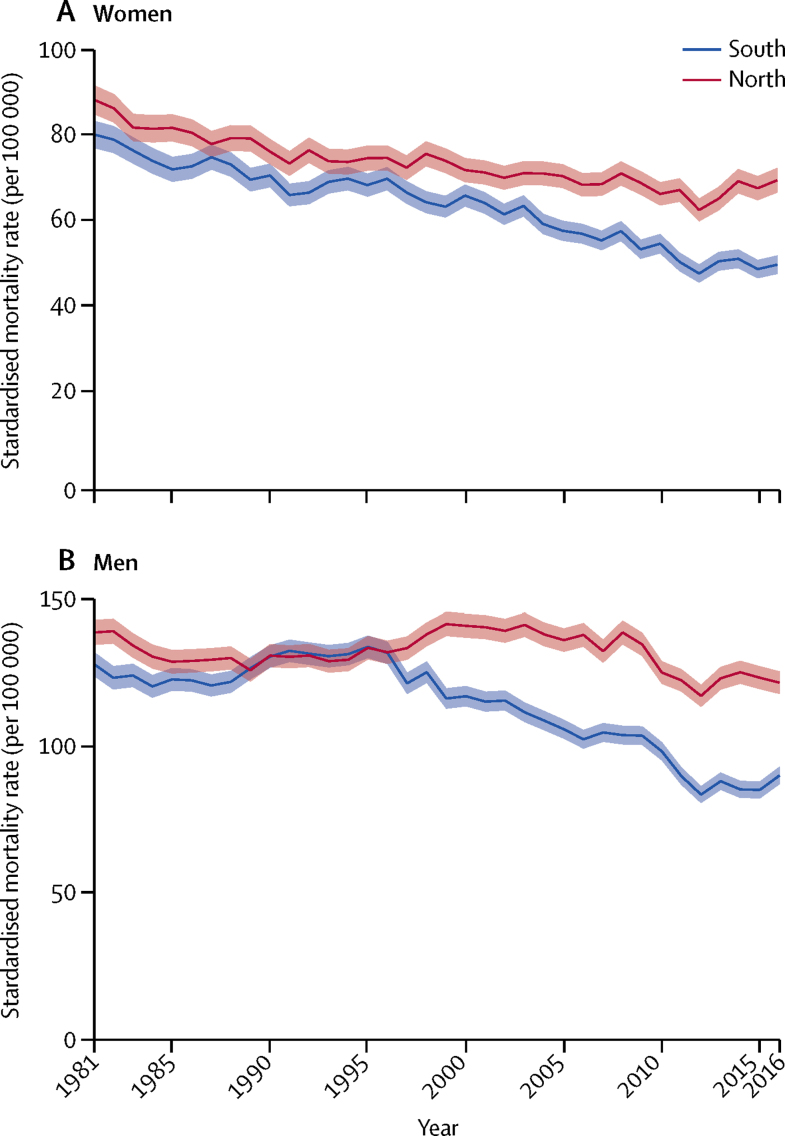
Age-standardised all-cause mortality for people aged 25–44 years in the north and the south of England from 1981 to 2016 in women (A) and men (B) Shaded areas are 95% CIs.

Age-standardised mortality rates from 1981 to 2016 suggested a marked and widening north–south gap among both men and women (figure 3). After 2010, mortality rates increased nationwide for both sexes, but much more sharply in the north than the south, resulting in a substantial gap by 2016. Underlying cause-specific plots of standardised mortality rates are provided in the appendix (pp 20–27); large gaps have emerged since the mid-1990s, in alcohol-related (appendix p 21) and drug-related (appendix p 24) deaths for both sexes. Rates in alcohol-related and drug-related deaths have increased in the north since the mid-1990s, while remaining relatively stable in the south. For accidents, a gap for men emerged in the late 1990s as mortality rates declined in the south, although rates appear to become similar between north and south around 2010 and again in 2015 (appendix p 20).

National mortality rates for cardiovascular deaths declined over the study period but a longstanding gap between north and south persisted (from 33·3 [95% CI 31·8–34·8] in 1981 to 15·0 [14·0–15·9] in 2016 in the north vs from 23·5 [–24·8] to 9·9 [9·2–10·5] in the south (appendix p 22). Cancer death rates (excluding breast cancer) decreased nationwide over time, but the decease plateaued from 2005 for women and 2010 for men, with small levels of northern excess mortality for women persisting (appendix p 25). Breast cancer mortality rates decreased over time, but plateaued after 2010, with no evident mortality excess (appendix p 26). Suicide rates have decreased since around 1998 in both sexes, and throughout most of the study period there was little difference between north and south. A gap in suicides emerged for men between 2010 and 2015 as rates climbed in the north (appendix p 23). The pattern for other causes of death declined over time in the south, for both men and women, with the exception of a spike for men in the south between 1988 and 1996, but remained largely stable in the north. However, increases in northen excess deaths in this category emerged around 2000 for men and 2005 for women.

**Figure 4:**
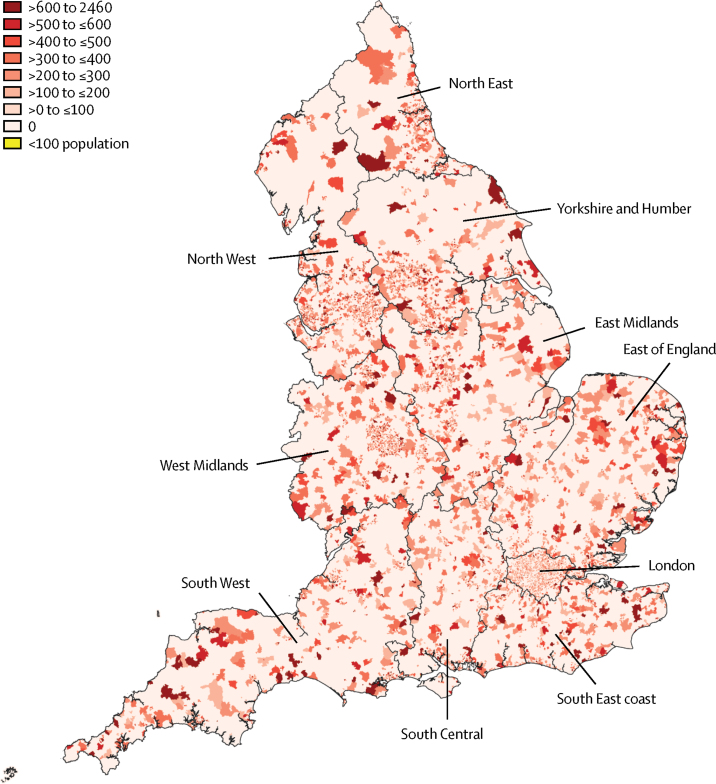
Age-standardised all-cause mortality for people aged 25–44 years in 2016 at small area geographical level for the whole of England* *Data for areas with fewer than 100 residents aged 25–44 years are not shown.

Spatial analysis showed clustering of high mortality in the post-industrial cities of the north, particularly in the North East and North West (figure 4). These standardised mortality rates were associated with socioeconomic deprivation, which is clustered in urban areas. Additional spatial maps, by sex and region, are provided in the appendix (pp 28–60).

**Figure 5:**
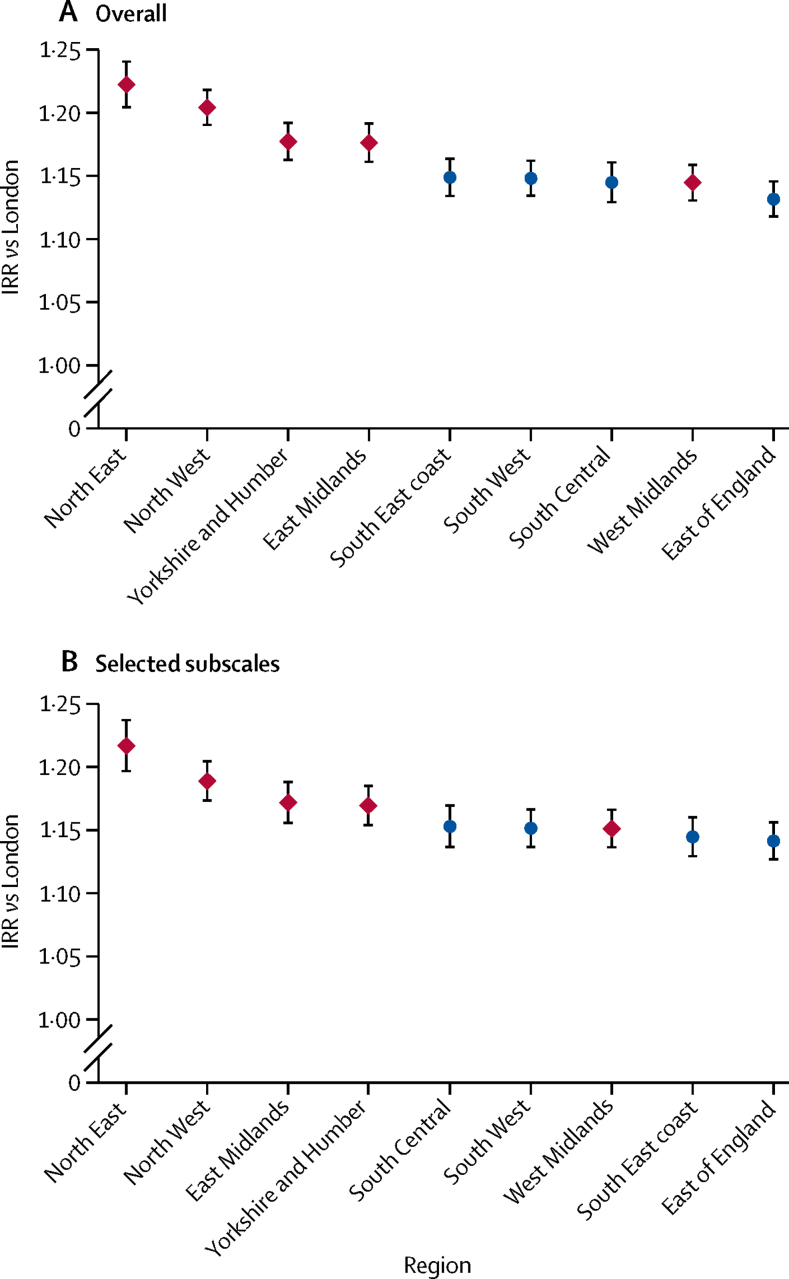
Regional age–sex adjusted incidence rate ratios for excess mortality in England, compared with London, adjusting for overall IMD (A) and selected IMD subscales (B) The 2015 IMD was used; domains in the bottom model were employment, housing, crime, and environment (driven by collinearity concerns, except health, which was removed owing to overlap with the outcome). Red dots represent northern regions and blue dots represent southern regions. Bars are 95% CIs. IMD=Index of Multiple Deprivation. IRR=incidence rate ratio.

We used several models to evaluate the role of deprivation on excess mortality in the north in 2016. In the first model, which did not include deprivation, the IRR for the association between area and excess mortality in the north was 1·15 (95% CI 1·14–1·15). In the second model, which included area and overall deprivation, the IRR was 1·07 (95% CI 1·06–1·07). In the third model, in which we fitted all ten English regions as categories instead of a north–south dichotomy, all regions reported higher IRRs compared with London and ranged from IRR 1·13 (1·12–1·15) in East England to the North West (1·20, 1·19–1·22) and North East (IRR 1·22, 95% CI 1·20–1·24; figure 5). In the fourth model, which included four subdomains of the IMD as covariates rather than the overall IMD, the IRR for the association between area and mortality was 1·05 (95% CI 1·04–1·05). In the fifth model, which included the ten English regions and these four IMD domains, we observed a similar regional pattern to the third model (figure 5). Pseudo-*R*^2^ reflected about 53% of explained variation for all models.

## Discussion

Against a background of longstanding excess mortality in the north of England for the whole population, there has been a rapid and marked divergence between northern and southern mortality for adults aged 25–44 years since the mid-1990s. Between 2014 and 2016, this divergence resulted in a mean annual excess of deaths in the north of 627 among women and 1177 among men. The major contributors to this disparity were drug misuse, alcohol misuse, and cardiovascular disease. Important contributions were also made by suicide among men, especially in those aged 30–34 years, and by cancer among women. Overall excess mortality in the north was only partially explained by deprivation measured at the low geography level. Regional analyses showed that much of the north–south divide is attributable to substantially lower mortality in London. Although mortality rates in most northern regions were higher than in southern regions, the IRR for the region with the lowest rates compared with London was 1·13.

The numbers of excess deaths in the north differed between men and women aged 25–44 years, with the north–south difference being almost double for men between 2014 and 2016. This disparity is the result of a combination of factors. Primarily, mortality rates for men are generally higher; therefore, comparable relative differences across sexes would yield higher absolute numbers of excess deaths in men in the north. Additionally, we observed a greater difference in mortality risk in men between the north and the south, compared with that in women between these two regions. This sex difference in the north–south mortality gap is plausibly related to greater susceptibility of men to socioeconomic pressures.^15^

The relative contribution of different causes of death towards excess mortality in the north has changed substantially over time. Cardiovascular deaths have been consistently higher in the north, and because cardiovascular disease remains a major contributor to total deaths, particularly in older men, it explains much of the mortality gap between north and south. However, cardiovascular disease mortality has decreased by over 50% over the past four decades, and its contribution to regional variations in mortality seems to be weakening.^16^

Of the broad causes we examined, three—accidents, alcohol related, and drug related—showed an increase in men in the north from the mid-1990s, coinciding with a widening gap compared with the south in all-cause mortality. For fatal accidents and drug-related deaths, there was further separation from 2010, with mortality rates increasing nationwide, but more quickly in the north than the south. If these recent patterns are not stopped, the national gains made from falling cardiovascular deaths will be over-ridden and excess mortality in the north could exceed 50%.^5^

Accidents, alcohol misuse, drug misuse, and suicide are all strongly associated with socioeconomic status. For example, in international studies, the most deprived men are twice as likely to die by suicide as the most affluent men, whereas the most deprived women are 1×5 times as likely to die by suicide.^17^ In England, suicide risk has also been associated with unemployment,^18^ and substantial increases in suicide have been observed during periods of recession, especially among men.^15^ However, physical health, mental health, and deprivation—strong independent risk predictors of suicide^19^—confound the relationship between unemployment and suicide.^20^ For accidents, there is a consistent association between socioeconomic deprivation and trauma incidence, especially penetrating serious trauma, and accident and emergency attendance.^21^ For road traffic fatalities, excessive speed, intoxication, failure to wear seatbelts, and unlicensed or uninsured driving are most prevalent in the most deprived areas in England, whereas pedestrian casualties are also associated with deprivation.^22^ Geographical analysis of fatal traffic accidents found that nine of the ten highest-risk counties are in the north of England, with nine of the ten lowest-risk counties in the south.^23^ In particular, there is a north–south divide with the north having higher mortality on single carriageways, where most road traffic casualties occur, with the exception of the South East (highest risk) and the West Midlands (lowest risk).^24^ Northern infrastructure might be relevant in this context, with transport infrastructure investment heavily skewed towards the south, especially London.^25^ Work-related fatal accidents are also higher in the north of England, mainly owing to variations in regional industries and occupations and their associated risks.^26^ Fatal domestic accidents, such as fires, are associated with risk factors linked to socioeconomic deprivation, including smoking and alcohol misuse.^27^

The role of alcohol is important in this context, contributing to a large proportion of accidental injuries seen in English hospitals, not only through traffic and cycling accidents, but also accidental falls.^28^ Alcohol use also underpins the steep and sustained increase in liver cirrhosis deaths in Britain from the 1990s,^29^ when the north–south divide in mortality for those aged 25–44 years started to emerge.^5^ Socioeconomic inequalities, in terms of alcohol-related mortality, are greatest among people aged 25–44, with five-fold and four-fold relative risks in the most deprived areas for men and women, respectively.^30^ Men aged 25–39 years in the unskilled manual socioeconomic class are ten to 20 times more likely to die from alcohol-related causes, compared with those in the professional class,^31^ with personal income being the best predictor of alcohol-related death for men.^27^ Additionally, people living in urban areas experience higher alcohol-related mortality compared with those living in rural areas, with the association being much stronger for men than for women.^32^

For almost all types of cancer, risk of death is higher and death is likely to occur more quickly in more deprived areas.^33^, ^34^ Particularly striking deprivation gradients are observed for cancers of the oropharynx and lung, which are primarily attributed to smoking.^35^ Similar disparities have been reported for cancer of the cervix, which is caused by human papillomavirus, and is largely determined by sexual behaviour risk factors, including age at first intercourse, number of sexual partners, failure to use a condom during intercourse with multiple partners, and screening programme non-engagement.^36^ Alcohol misuse also plays a role in cancer mortality, with steep socioeconomic gradients for cancer incidence and survival observed for stomach and liver cancers.^37^ After alcohol, heroin and crack cocaine are the most harmful drugs at the population level in England, and deaths from drug overdoses are also strongly associated with deprivation,^38^ with evidence of strong cohort effects emerging.^39^

More research is needed to establish what triggered the observed divergence in mortality between north and south for young and middle-aged adults since the mid-1990s, but socioeconomic factors are likely to be central.^40^ For example, in the USA, mortality rates have been rising for white non-Hispanics without a college degree and falling for those with a degree. Mechanisation, which has affected a large number of mid-level jobs in developed countries, might be partly to blame for the increased mortality in more deprived strata of populations worldwide. Although it has also led to an increase in demand for high-skilled jobs, there are fewer jobs created than the number of mid-level jobs lost, and individuals from a deprived background are at a disadvantage when competing for the high-skilled jobs.^41^

However, our findings of a persistent north–south divide, even after controlling for area-level deprivation, suggest there must be additional explanatory factors related to geography—for example, net migration (internal and external) of healthier individuals to the south of England.^42^ Furthermore, the mortality trends for cardiovascular dsease are known to be driven by socioeconomic factors, suggesting that divergence between north and south is not inevitable. For cardiovascular disease, worsening temporal trends in some risk factors (eg, physical activity, obesity, and diabetes) have been offset by decreases in others (eg, smoking cessation and more optimal blood pressure and cholesterol control).^43^ National cardiovascular initiatives have included targeted elements resulting in both overall reductions in mortality and some reductions in inequalities, particularly in younger age groups,^44^ although the north–south divide in cardiovascular mortality remains. For example, the National Service Framework in coronary heart disease promoted assessment and treatment of cardiovascular risk in patients deemed to be at higher risk, the redesign of heart attack centres, and the roll out of primary percutaneous coronary interventions, yet the gap remains.^45^ Similar coordinated initiatives, both tackling underlying causes and improving health service responses, will be needed to reverse the marked recent trends in mortality attributable to accidents, alcohol misuse, and drug misuse.

Our study has some limitations. First, the north–south dichotomy as analysed here is one of convenience, although it reflects administrative boundaries delineating areas of political responsibility, as well as established social, economic, and cultural divisions. Second, the categorisation of underlying causes of death enabled us to attribute most of the deaths to cohesive groups of causes, but many were also necessarily placed in the other category. Third, a small number of deaths attributed to diabetes, and even fewer to obesity, were included in cardiovascular deaths. Our aim was to generate an inclusive category, since these underlying causes are often linked. Fourth, the IMD includes indicators that are closely linked to mortality (eg, road traffic accidents), albeit with a time lag in the 2016 data that we used. Fifth, we assumed a uniform population distribution within each age group, which might not be the case for London. Sixth, there is a delay in registration for one in five premature deaths, and some of our estimates may not accurately reflect time of death. Seventh, the role of internal and external migration is not taken into account and our findings will be at least partially explained by the migration of healthy individuals, attracted by better job opportunities to the south, especially, London.^46^ Eighth, one could argue that London is unique and an alternative north–south comparison should exclude London, to quantify the extent to which the north–south differences are driven by the population of London. However, we could not obtain detailed mortality data at the regional level, only aggregated into north and south. This aspect should be explored in future work, as well as the role of cohort effects.

In summary, the north–south divide in mortality for people aged 25–44 years first emerged during the mid-1990s and continued into 2016. This mortality divide grew quickly over that period for accidents (in men), alcohol misuse, and drug misuse, while a longstanding gap for cardiovascular deaths remained and a gap for suicide in men emerged more recently. The sharp rises in mortality for alcohol misuse, and drug misuse are concerning. Regional analysis confirms that most of the north is faring worse than most of the south, but further shows that all regions, north and south, have substantially higher mortality rates than London. The reasons for this are complex and reach back centuries, with extreme concentration of power, wealth, and opportunity in London, which without major structural change could continue to damage public health.

For the **ONS data** see https://www.ons.gov.uk/peoplepopulationandcommunity/birthsdeathsandmarriages/deaths/adhocs/008228deathsregisteredinthenorthandsouthofengland1981to2016, and https://www.ons.gov.uk/peoplepopulationandcommunity/birthsdeathsandmarriages/deaths/adhocs/006148deathsregisteredinenglandbylowersuperoutputarea2005to2015

**This online publication has been corrected. The corrected version first appeared at thelancet.com/public-health on October 31, 2018**

## Data sharing
