# Correction to *Lancet Public Health* 2018; 3: 567–75

**DOI:** 10.1016/S2468-2667(18)30231-7

**Published:** 2018-11-01

*Kontopantelis E, Buchan I, Webb RT, Ashcroft DM, Mamas MA, Doran T. Disparities in mortality among 25–44-year-olds in England: a longitudinal population-based study.* Lancet Public Health *2018; **3:** 567–75—*In figure 5 of this Article, the labels on the x-axis for the overall data in figure 5A were presented as the same for the labels on the x-axis for the selected subscales in figure 5B. The corrections to the labels on the x-axis for figure 5A have been made to the online version as of Oct 31, 2018.

