# Supplementary material for: Disparities in mortality among 25–44-year-olds in England: a longitudinal, population-based study
Source: Lancet Public Health. 2018 Oct 31;3(12):e567–75. doi: 10.1016/S2468-2667(18)30177-4 (PMC6277813; doi:10.1016/S2468-2667(18)30177-4)
Supplement: Supplementary appendix [file mmc1.pdf]

THE LANCET
Public Health

## Supplementary appendix

This appendix formed part of the original submission and has been peer reviewed. We post it as supplied by the authors.

Supplement to: Kontopantelis E, Buchan I, Webb RT, Ashcroft DM, Mamas MA, Doran T. Disparities in mortality among 25–44-year-olds in England: a longitudinal population-based study. *Lancet Public Health* 2018; published online Oct 30. http://dx.doi.org/10.1016/S2468-2667(18)30177-4.

# Supplementary file for: *Explaining North-South disparities in mortality rates among 25-44 year olds in England: a longitudinal population study*

## Methods

### Data sources

The Office for National Statistics (ONS) provided data on cause-specific mortality from. Death certificates in England record a single underlying cause of death, which is assigned an ICD-10 code (or ICD-9, before January 2001). This information is used by ONS to routinely group deaths into broad categories. We obtained cause-specific mortality data aggregated into the following groups: accidents, alcohol-related, cardiovascular disease and diabetes mellitus, drug poisoning, suicide, malignant neoplasm (i.e. cancer but excluding breast cancer), malignant neoplasm of the breast, and all 'other' causes combined. ICD-9 and ICD-10 codes for each group are provided in the supplement (Tables S1 to S4). For alcohol related deaths, the ONS definition changed in 2016 following a consultation: we used the older definition to include partially attributable conditions, like cirrhosis of the liver. To obtain mutually exclusive groupings we requested a bespoke dataset as some deaths are recorded in more than one category by the ONS as standard. Accidental poisoning by drugs was only included under drug-poisoning (and not under accidents); intentional or undetermined intent self-poisoning by drugs under suicide (and not drug poisoning); accidental poisoning by alcohol under alcohol-related (and not accidents); and intentional self-poisoning by alcohol under suicides (and not alcohol-related). Data were available from 1981 to 2016 for all causes except drug poisoning, which were available from 1993. We also derived mid-year population estimates from ONS for the years 1993 to 2016. These estimates are based on decennial Censuses in 1991, 2001 and 2011, with interpolated estimates for the remaining years (extrapolated for 2012 onwards). Death and population data were stratified by sex and into 5-year age groups.

As for previous studies,[1,2] deaths and population data were aggregated into the ten Government Office Regions which were then categorised as either North (North East, North West, Yorkshire & the Humber, East Midlands and West Midlands) or South (East of England, South Central, South West, South East and London). In 2016, there were estimated to be 14,900,946 males and 14,559,738 females in the South, compared to 13,066,201 males and 12,741,182 females in the North. For the 25-44 age-group, there were 4,084,954 males and 4,107,009 females in the South, compared to 3,239,147 males and 3,222,377 females in the North.

Additionally, all-cause mortality and population data for 2016 were obtained for small geographical areas: lower layer super output area (LSOA).[3] Following the 2011 Census there were 32,844 LSOAs in England, with a mean population of 1,620.[4] We also measured area deprivation at the LSOA level using the 2015 English Index of Multiple Deprivation (IMD). The IMD is the most comprehensive aggregate measure of English deprivation, covering seven domains: income, employment, health, education and skills, housing, crime, and the environment.[5]

## Statistical analyses

Cause-specific mortality rates were calculated from death registrations and mid-year population estimates and examined by age group, sex and area dichotomy (North vs. South of England) over time. For each of the groups by cause of death (accidents, alcohol-related, cancer - excluding breast cancer, breast cancer in women, cardiovascular disease and diabetes, drug poisoning, suicide, and all other causes) we used Poisson regression models to calculate excess mortality rates in the North in each calendar year, adjusted for the age distribution of the population in the two respective areas; separate models were ran for each sex.

The estimated Incidence Rate Ratios (IRRs), which can be interpreted as the percentage of population-adjusted excess mortality in the North compared to the South, were depicted as sex-specific contour plots, by sex, to visualise changes in excess mortality according to age groups as a time-dependent variable. Using the IRR estimates from these models and the population structure, we also estimated the number of adjusted excess deaths in the North by underlying cause, cross-sectionally for the last three years of the study period (2014 to 2016). These are presented as stacked bar plots by 5-year age-bands of numbers and percentages.

Standardised Mortality Rates (SMRs) per 100,000 people, by age and sex and with the total respective population of the study period as the reference (from 1981 or 1993 depending on the underlying cause),[6] were calculated for each calendar year and by area and plotted over time. Although estimating SMRs for small geographical areas can be problematic due to small numbers and imprecise estimation, we nonetheless used the 2016 all-cause mortality data at the LSOA level to obtain such estimates, allowing us to visualise their spatial distribution.

To evaluate the role of measured deprivation in excess mortality in the North, we fitted Poisson regression models with the unit of analysis being the LSOA level. The outcome was all-cause mortality in 2016 and the two covariates of interest were area and deprivation, while all models were adjusted for the age-sex population structure. The first model only included area (North vs South), a second one included area (North vs. South) and the overall IMD, and a third included the 10 English regions (rather than an area dichotomy) and the overall IMD. Since the IMD includes a health domain with a subdomain in premature mortality (albeit predating our outcome),[7] we ran two more models where we included specific domains of the IMD rather than the aggregate score. Besides the health domain, we also excluded income and education due to very strong collinearity with employment, which is known to be important in this context.[8] Therefore, the fourth model included area (North vs. South) and the employment, housing, crime and environment IMD domains. The fifth and final model included the 10 English regions and these four IMD domains. Stata v15 was used for all analyses, and all IRR and SMR estimates are reported with 95% confidence intervals.

## Limitations

As mentioned in the main paper, we assumed a uniform population distribution within each age stratum, something that does not necessarily stand for London. London is an anomaly in terms of the population, as shown below in Figure A. So it very likely that for at least two of the age groups (and possibly 3) the age distribution is not uniform (i.e. we would have more 35 year olds than 39 year olds in the 35-39 stratum). However, this should only modestly affect the mean age within each of our age

groups. For example, we estimate the mean ages in the East of England and London from the numbers shown in the graph below (under the uniform assumption) and we obtain: 34.9 (F) and 34.8 (F) for the East; 34.1 (F) and 34.2 (M) for London. Of course, as explained, the uniform assumption does not hold for London, but this calculation aims to show that, for London and if similar distributional patterns can be assumed for each of the age groups we investigated, that would lead to very small regional differences in the means of each group (arguably, approximately a fourth of what we reported before, around 0.2 years). Therefore, we conclude that the risk in death differences across regions and especially London, because of non-uniform distributions within age-groups, should be small. However, they will of course explain a small part of the effect reported in Figure 5 of the main paper.

Figure A: Population in 2016 by age group and region, males and females

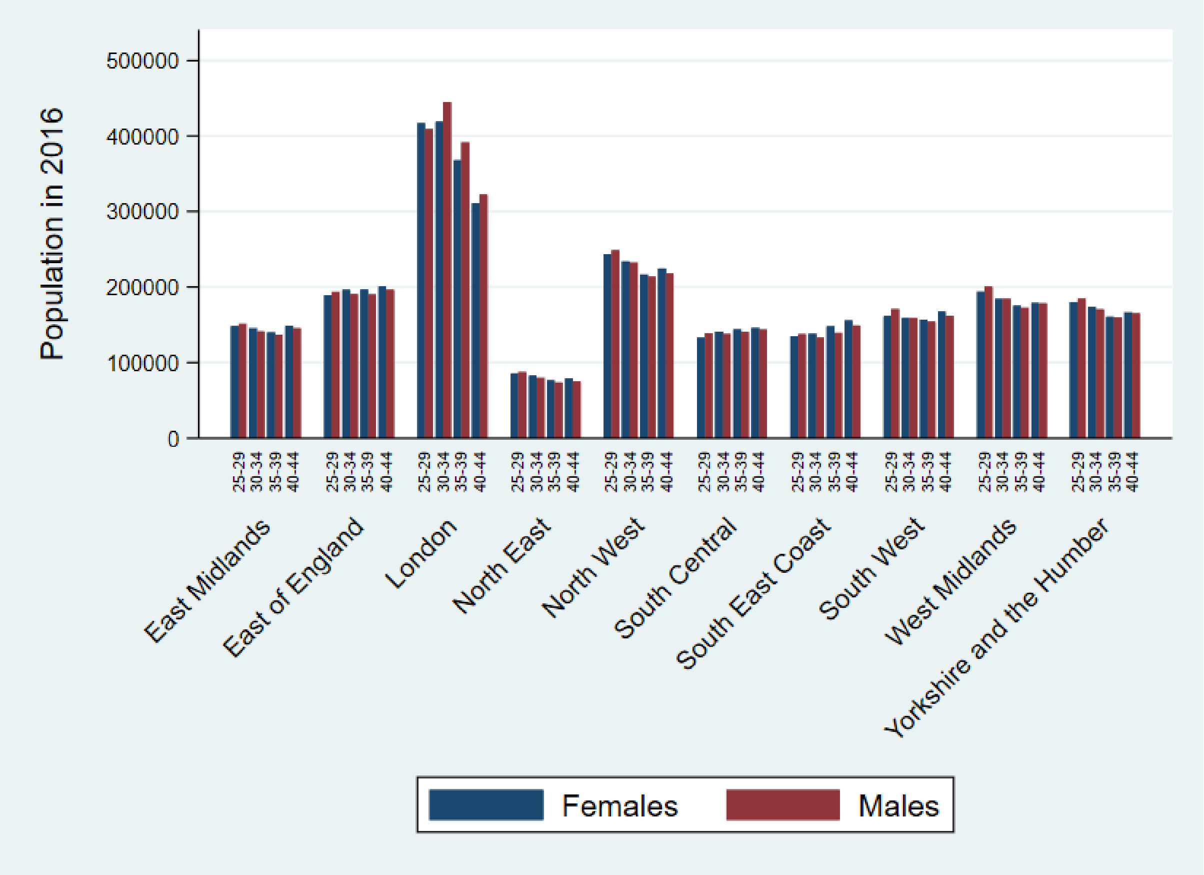


## References

1. Buchan IE, Kontopantelis E, Sperrin M, Chandola T, Doran T. North-South disparities in English mortality 1965-2015: longitudinal population study. *J Epidemiol Commun H* 2017; **71**(9): 928-36.

2. Hacking JM, Muller S, Buchan IE. Trends in mortality from 1965 to 2008 across the English north-south divide: comparative observational study. *Brit Med J* 2011; **342**.

3. Office for National Statistics. Super Output Area mid-year population estimates for England and Wales, Mid-2011 (Census Based). http://www.ons.gov.uk/ons/publications/re-reference-tables.html?edition=tcm%3A77-2856292016).

4. Office for National Statistics. Changes to Output Areas and Super Output Areas in England and Wales, 2001 to 2011, 2012.

5. Communities and Local Government. The English Indices of Deprivation 2015: Technical Report: Department for Communities and Local Government, 2015.

6. Kontopantelis E, Springate DA, Ashworth M, Webb RT, Buchan IE, Doran T. Investigating the relationship between quality of primary care and premature mortality in England: a spatial whole-population study. *Bmj-Brit Med J* 2015; **350**.
7. Kontopantelis E, Mamas M, van Marwijk H, Ryan AM, Doran T. Geographical epidemiology of health and overall deprivation in England, its changes and persistence from 2004 to 2015. *Journal of Epidemiology & Community Health,* 2017.
8. Stuckler D, Basu S, Suhrcke M, Coutts A, McKee M. The public health effect of economic crises and alternative policy responses in Europe: an empirical analysis. *Lancet* 2009; **374**(9686): 315-23.

Table S1: International Classification of Diseases (ICD) codes used to define deaths related to drug poisoning[1]

| Description | ICD-9 Codes | ICD-10 Codes |
|---|---|---|
| Mental and behavioural disorders due to drug use (excluding alcohol and tobacco) | 292, 304, 305.2–305.9 | F11–F16, F18–F19 |
| Accidental poisoning by drugs, medicaments and biological substances | E850–E858 | X40–X44 |
| Assault by drugs, medicaments and biological substances | E962.0 | X85 |

[1] Intentional self-poisoning by drugs, medicaments and biological substances, ICD-9 codes E950.0–E950.5 & ICD-10 codes X60–X64 and poisoning by drugs, medicaments and biological substances, undetermined intent, ICD-9 codes E980.0–E980.5 & ICD-10 codes Y10-Y14 are excluded from drug related deaths and included in suicides.

Table S2: International Classification of Diseases (ICD) codes used to define deaths related to suicide

| Description | ICD-9 codes | ICD-10 codes |
|---|---|---|
| Intentional self-harm | E950-E959 | X60-X84 |
| Injury/poisoning of undetermined intent | E980-E989[1,2] | Y10-Y34[2,3] |

[1] Excluding E988.8 for England and Wales.

[2] Excluding injury/poisoning of undetermined intent for persons aged 10-14.

[3] Excluding Y33.9 where the coroner's verdict was pending in England and Wales for the years 2001-2006. From 2007 onwards, deaths which were previously coded to Y33.9 are coded to U50.9.

Table S3: International Classification of Diseases (ICD) codes used to define deaths related to alcohol[1 2]

| Description | ICD type | code |
|---|---|---|
| Mental and behavioural disorders due to use of alcohol | 10 | F10 |
| Degeneration of nervous system due to alcohol | 10 | G31.2 |
| Alcoholic polyneuropathy | 10 | G62.1 |
| Alcoholic cardiomyopathy | 10 | I42.6 |
| Alcoholic gastritis | 10 | K29.2 |
| Alcoholic liver disease | 10 | K70 |
| Chronic hepatitis, not elsewhere classified | 10 | K73 |
| Fibrosis and cirrhosis of liver (Excluding K74.3-K74.5 - Biliary cirrhosis) | 10 | K74 |
| Alcohol induced chronic pancreatitis | 10 | K86.0 |
| Accidental poisoning by and exposure to alcohol | 10 | X45 |
| Poisoning by and exposure to alcohol, undetermined intent | 10 | Y15 |
| Alcoholic psychoses | 9 | 291 |
| Alcohol dependence syndrome | 9 | 303 |
| Non-dependent abuse of alcohol | 9 | 305 |
| Alcoholic cardiomyopathy | 9 | 425.5 |
| Alcoholic fatty liver | 9 | 571 |
| Acute alcoholic hepatitis | 9 | 571.1 |
| Alcoholic cirrhosis of liver | 9 | 571.2 |
| Alcoholic liver damage, unspecified | 9 | 571.3 |
| Chronic hepatitis | 9 | 571.4 |
| Cirrhosis of liver without mention of alcohol | 9 | 571.5 |
| Other chronic nonalcoholic liver disease | 9 | 571.8 |
| Unspecified chronic liver disease without mention of alcohol | 9 | 571.9 |
| Accidental poisoning by alcohol | 9 | E860 |

[1] The definition of alcohol related death has recently changed, however, for the purpose of this dataset the old definition has been used for consistency.
[2] 'Intentional self-poisoning by and exposure to alcohol' ICD-10 code X65 is excluded from alcohol related deaths but included in the number of suicides. There is no equivalent ICD-9 code.

Table S4: International Classification of Diseases (ICD) codes used to define all other causes

| Description | ICD-9 codes | ICD-10 codes |
|---|---|---|
| Accidents[1] | E800-E928 (excluding E870-E879, E850-E858 & E860) | V01-X59 (excluding X40-X44 & X45) |
| Cardiovascular (Diseases of the circulatory system) | 390-459 | I00-I99 |
| Diabetes mellitus | 250 | E10-E14 |
| Obesity | 278 | E66 |
| Malignant neoplasms (all except breast cancer) | 140-208 (excluding 174-175) | C00-C97 (excluding C50) |
| Malignant neoplasm of breast | 174-175 | C50 |

[1] 'Accidental poisoning by drugs, medicaments and biological substances', ICD-9 codes E850-E858 & ICD-10 codes X40-X44 and 'Accidental poisoning by and exposure to alcohol' ICD-9 code E86.0 and ICD-10 code X45 are excluded from accidents but included in drug and alcohol related deaths respectively.
[2] ONS does not have a standard definition of obesity. There is an ICD code to identify obesity however there will be an undercount of obesity related deaths if we only count deaths where was recorded as the underlying cause. The best way that we can capture obesity deaths is if we search for any mention of the ICD code on the death certificate - however it will still be an undercount because a lot of obesity related deaths will be coded to other things such as diabetes and heart disease. The numbers we can extract using the underlying cause code for obesity will not reflect the true number of obesity related deaths. Also, deaths might be higher in recent years because there is more awareness of the condition and of it being recorded as a cause of death, the figures might look like obesity has become much more common, but they will not be an accurate reflection of underlying trends (whether obesity is actually on the rise or not).

## Notes on all plots

1) Cardiovascular category includes diabetes mellitus and obesity

## Notes on contour plots, Figures S2-S10

2) cells with fewer than 20 deaths were suppressed to aid visualisation
3) Although the range of IRRs (% of excess mortality) varies by sex, we have tried to use the same plotting range to allow for easier cross-sex comparisons. Therefore, some cells are necessarily outside this plotting range. We stopped short of using the same plotting range across all graphs since the IRR range varies greatly across underlying causes of death.
4) Contour plots report ratios and hence the role and weight of each underlying cause in the mortality excess in the North, is not obvious. These graphs are meant to be interpreted in conjunction with the relevant SMR plots (S12-S19) from which the importance of each underlying cause can be inferred (from the Y-axis). The order of importance, in terms of contribution to the excess can also be derived from Tables S5-S10 and main paper Figure 2.

Figure S1: Percentages of deaths attributed to each of the investigated underlying causes between 2014 and 2016, for females (top) and males (bottom)*

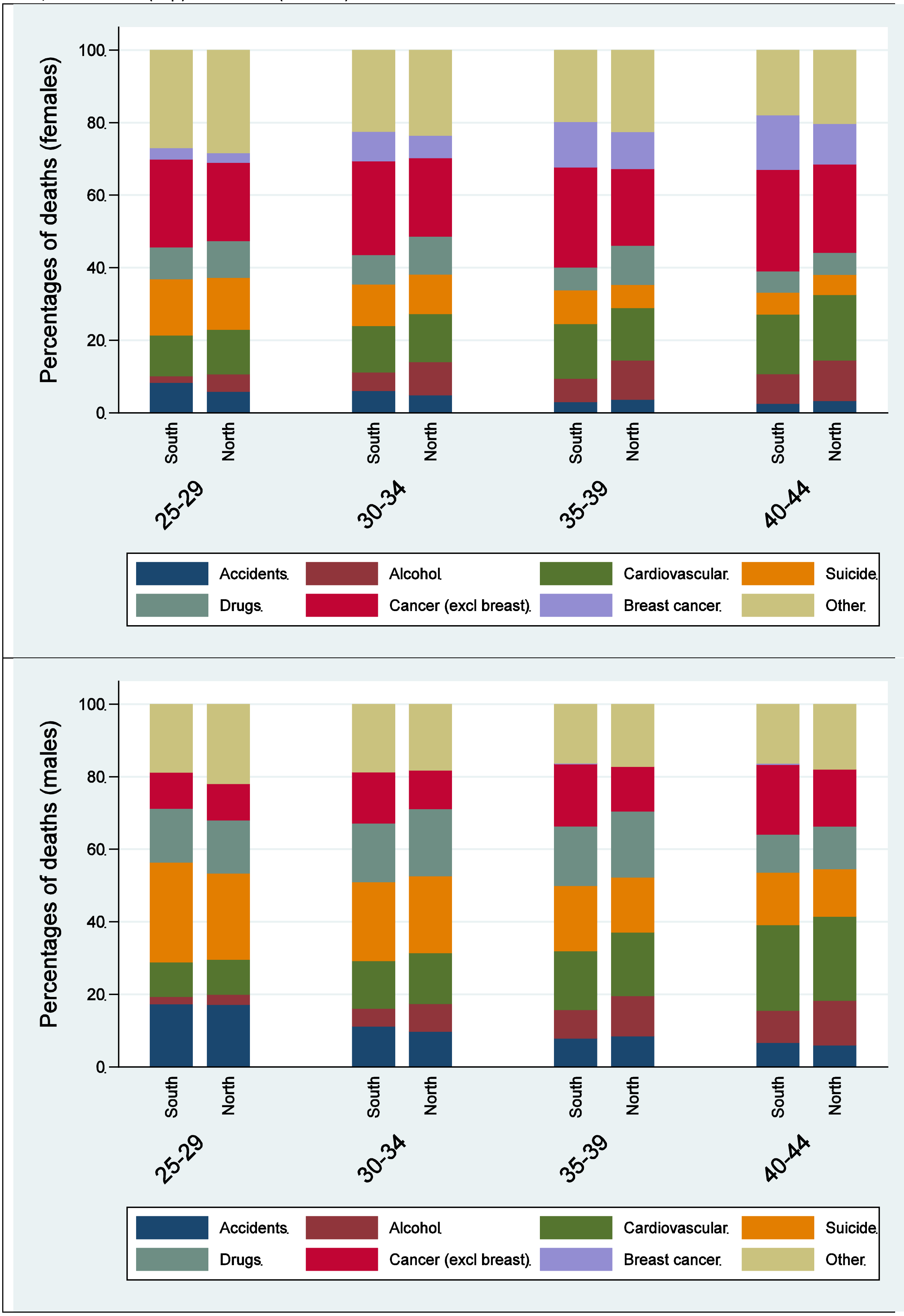


*Cardiovascular includes diabetes and obesity, with the overwhelming majority of deaths attributed to cardiovascular

Table S5: Numbers of excess deaths in the North by underlying cause of death 2014-16, females

| | **Excess North deaths estimate (95% Confidence Interval)** | | | |
|---|---|---|---|---|
| **Underlying cause of death** | **Aged 25-29** | **Aged 30-34** | **Aged 35-39** | **Aged 40-44** |
| Accidents | -7(-39,8) | 5(-24,19) | 32(10,42) | 41(16,55) |
| Alcohol | 27(9,31) | 66(45,77) | 123(99,138) | 153(115,179) |
| Cardiovascular | 25(-4,41) | 45(12,66) | 86(48,113) | 174(121,215) |
| Suicide | 14(-20,35) | 30(-3,50) | 6(-29,28) | 31(-6,55) |
| Drugs | 24(-3,37) | 55(28,70) | 126(103,141) | 53(17,76) |
| Cancer (excluding breast) | 17(-23,43) | 30(-17,63) | 59(7,99) | 102(31,160) |
| Breast cancer | 1(-27,8) | 2(-30,19) | 38(0,63) | -3(-60,40) |
| Other | 52(12,79) | 85(44,114) | 180(139,211) | 209(155,252) |
| Total | 153(-95,282) | 318(55,478) | 650(377,835) | 760(389,1032) |

Table S6: Numbers of excess deaths in the North by underlying cause of death 2014-16, males

| | **Excess North deaths estimate (95% Confidence Interval)** | | | |
|---|---|---|---|---|
| **Underlying cause of death** | **Aged 25-29** | **Aged 30-34** | **Aged 35-39** | **Aged 40-44** |
| Accidents | 63(19,95) | 37(-4,65) | 99(64,124) | 65(20,98) |
| Alcohol | 21(0,29) | 91(65,107) | 179(146,202) | 303(258,338) |
| Cardiovascular | 39(3,61) | 100(59,130) | 207(159,243) | 333(257,397) |
| Suicide | 41(-15,84) | 123(71,163) | 100(47,141) | 153(91,203) |
| Drugs | 53(11,82) | 152(108,185) | 223(175,260) | 218(166,258) |
| Cancer (excluding breast) | 39(3,62) | 9(-38,42) | 25(-30,67) | 105(32,165) |
| Breast cancer | 0(.,.) | 0(.,.) | 0(.,.) | 0(.,.) |
| Other | 126(82,159) | 103(54,140) | 198(150,235) | 325(263,376) |
| Total | 382(103,572) | 615(315,832) | 1031(711,1272) | 1502(1087,1835) |

Table S7: Percentages of excess deaths in the North by underlying cause of death 2014-16, females

| | **Percentage contribution to deaths** | | | |
|---|---|---|---|---|
| **Underlying cause of death** | **Aged 25-29** | **Aged 30-34** | **Aged 35-39** | **Aged 40-44** |
| Accidents | -4.6% | 1.6% | 4.9% | 5.4% |
| Alcohol | 17.6% | 20.8% | 18.9% | 20.1% |
| Cardiovascular | 16.3% | 14.2% | 13.2% | 22.9% |
| Suicide | 9.2% | 9.4% | 0.9% | 4.1% |
| Drugs | 15.7% | 17.3% | 19.4% | 7.0% |
| Cancer (excluding breast) | 11.1% | 9.4% | 9.1% | 13.4% |
| Breast cancer | 0.7% | 0.6% | 5.8% | -0.4% |
| Other | 34.0% | 26.7% | 27.7% | 27.5% |
| Total | 100% | 100% | 100% | 100% |

Table S8: Percentages of excess deaths in the North by underlying cause of death 2014-16, males

| | **Percentage contribution to deaths** | | | |
|---|---|---|---|---|
| **Underlying cause of death** | **Aged 25-29** | **Aged 30-34** | **Aged 35-39** | **Aged 40-44** |
| Accidents | 16.5% | 6.0% | 9.6% | 4.3% |
| Alcohol | 5.5% | 14.8% | 17.4% | 20.2% |
| Cardiovascular | 10.2% | 16.3% | 20.1% | 22.2% |
| Suicide | 10.7% | 20.0% | 9.7% | 10.2% |
| Drugs | 13.9% | 24.7% | 21.6% | 14.5% |
| Cancer (excluding breast) | 10.2% | 1.5% | 2.4% | 7.0% |
| Breast cancer | 0.0% | 0.0% | 0.0% | 0.0% |
| Other | 33.0% | 16.7% | 19.2% | 21.6% |
| Total | 100% | 100% | 100% | 100% |

Table S9: Incidence rate rations in the North vs South comparison by underlying cause of death 2014-16,, females

| | **Incidence rate ratios (95% Confidence Interval)** | | | |
|---|---|---|---|---|
| **Underlying cause of death** | **Aged 25-29** | **Aged 30-34** | **Aged 35-39** | **Aged 40-44** |
| Accidents | 0.87(0.54,1.20) | 1.09(0.72,1.46) | 1.87(1.18,2.57) | 1.74(1.20,2.29) |
| Alcohol | 3.33(1.29,5.38) | 2.42(1.67,3.17) | 2.58(1.97,3.19) | 1.83(1.52,2.14) |
| Cardiovascular | 1.35(0.96,1.74) | 1.38(1.08,1.69) | 1.47(1.22,1.73) | 1.47(1.29,1.65) |
| Suicide | 1.15(0.85,1.44) | 1.29(0.98,1.59) | 1.06(0.80,1.31) | 1.23(0.96,1.49) |
| Drugs | 1.42(0.97,1.88) | 1.74(1.28,2.19) | 2.67(2.03,3.31) | 1.40(1.10,1.70) |
| Cancer (excluding breast) | 1.11(0.88,1.34) | 1.12(0.94,1.31) | 1.18(1.02,1.34) | 1.16(1.04,1.28) |
| Breast cancer | 1.03(0.43,1.63) | 1.02(0.72,1.33) | 1.25(1.00,1.50) | 0.99(0.85,1.14) |
| Other | 1.30(1.06,1.55) | 1.41(1.18,1.65) | 1.75(1.50,2.01) | 1.52(1.34,1.70) |
| Total | 1.24(1.12,1.37) | 1.35(1.24,1.46) | 1.54(1.44,1.64) | 1.34(1.27,1.41) |

Table S10: Incidence rate rations in the North vs South comparison by underlying cause of death 2014-16, males

| | **Incidence rate ratios (95% Confidence Interval)** | | | |
|---|---|---|---|---|
| **Underlying cause of death** | **Aged 25-29** | **Aged 30-34** | **Aged 35-39** | **Aged 40-44** |
| Accidents | 1.28(1.07,1.49) | 1.21(0.98,1.43) | 1.63(1.33,1.93) | 1.28(1.07,1.50) |
| Alcohol | 1.79(1.00,2.58) | 2.13(1.62,2.65) | 2.13(1.76,2.50) | 1.99(1.74,2.25) |
| Cardiovascular | 1.31(1.02,1.60) | 1.47(1.23,1.71) | 1.63(1.43,1.84) | 1.41(1.29,1.53) |
| Suicide | 1.11(0.96,1.26) | 1.35(1.17,1.52) | 1.28(1.11,1.44) | 1.31(1.16,1.45) |
| Drugs | 1.27(1.05,1.50) | 1.58(1.35,1.80) | 1.67(1.46,1.88) | 1.60(1.40,1.80) |
| Cancer (excluding breast) | 1.30(1.02,1.58) | 1.04(0.86,1.22) | 1.07(0.92,1.22) | 1.16(1.04,1.27) |
| Breast cancer | 1.00(.,.) | 1.00(.,.) | 1.00(.,.) | 1.00(.,.) |
| Other | 1.51(1.28,1.74) | 1.34(1.15,1.52) | 1.60(1.40,1.81) | 1.58(1.42,1.73) |
| Total | 1.29(1.21,1.38) | 1.38(1.30,1.46) | 1.51(1.43,1.59) | 1.44(1.38,1.50) |

Figure S2: All-cause excess mortality in the North compared to the South, females (top) and males (bottom)

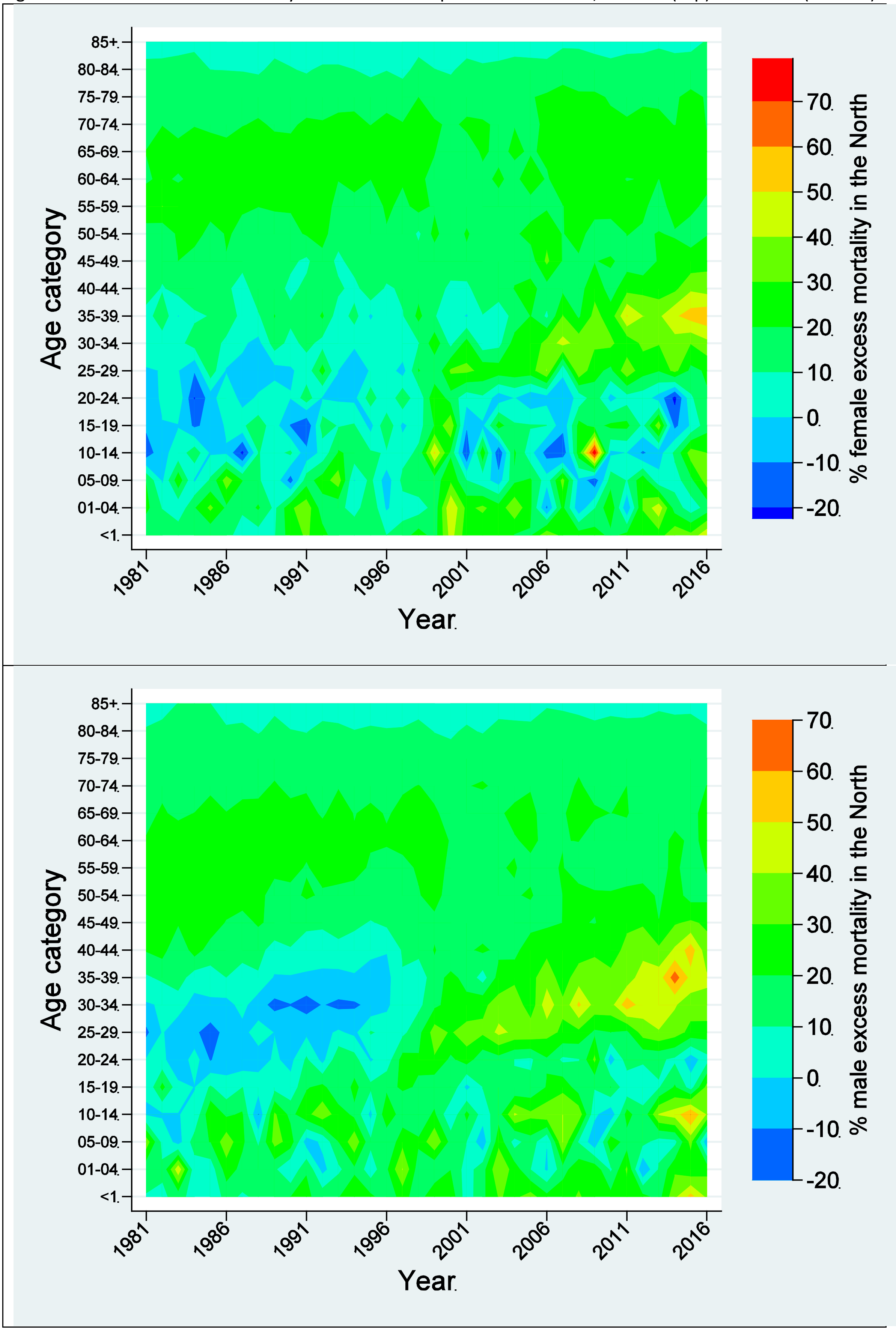

Figure S3: Accident related excess mortality in the North compared to the South, females (top) and males (bottom)

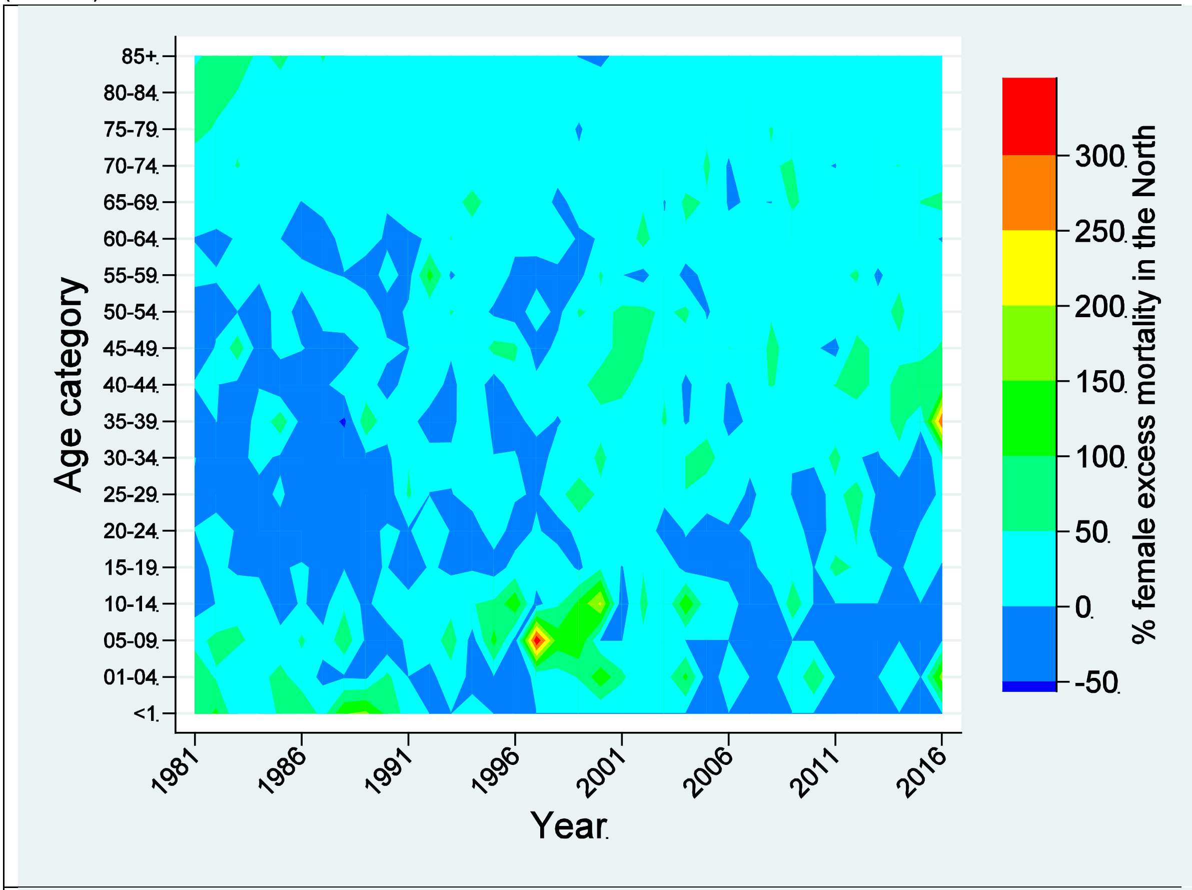


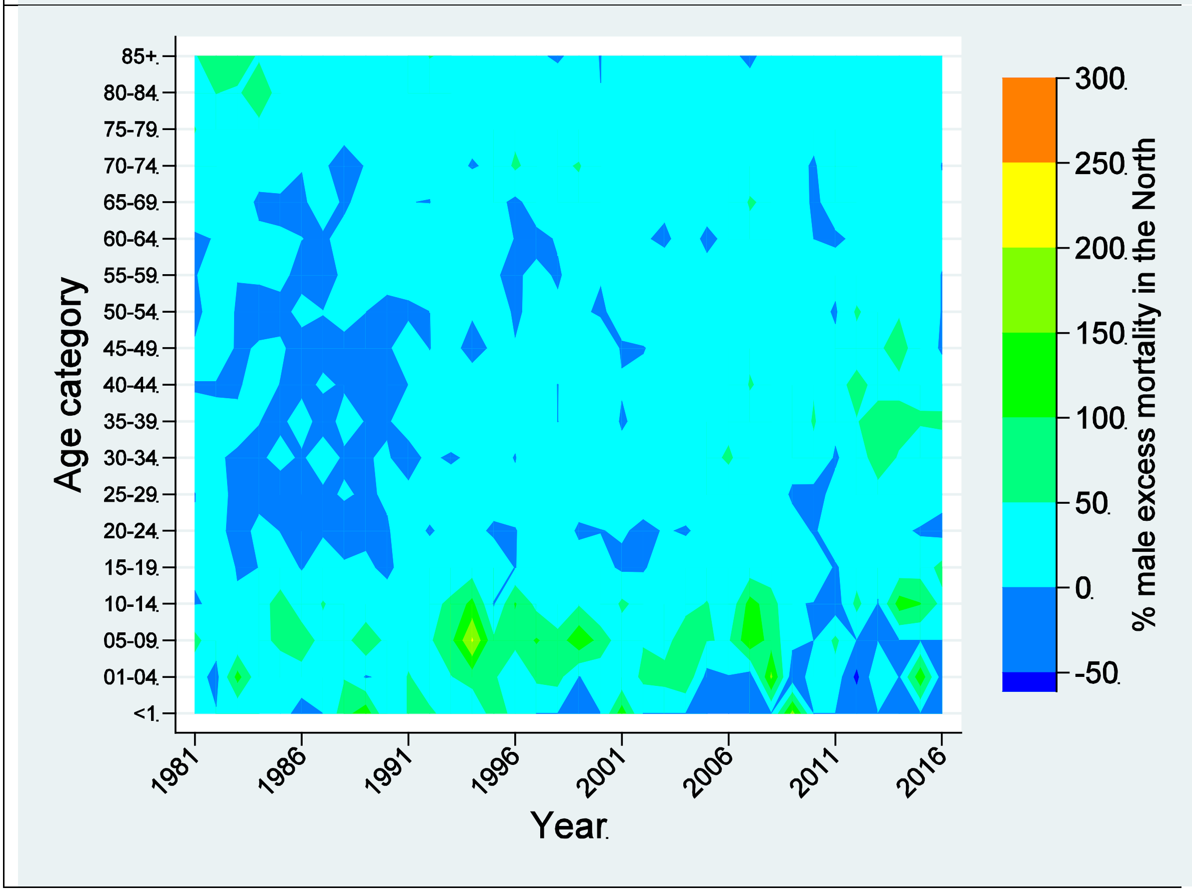

Figure S4: Alcohol related excess mortality in the North compared to the South, females (top) and males (bottom)

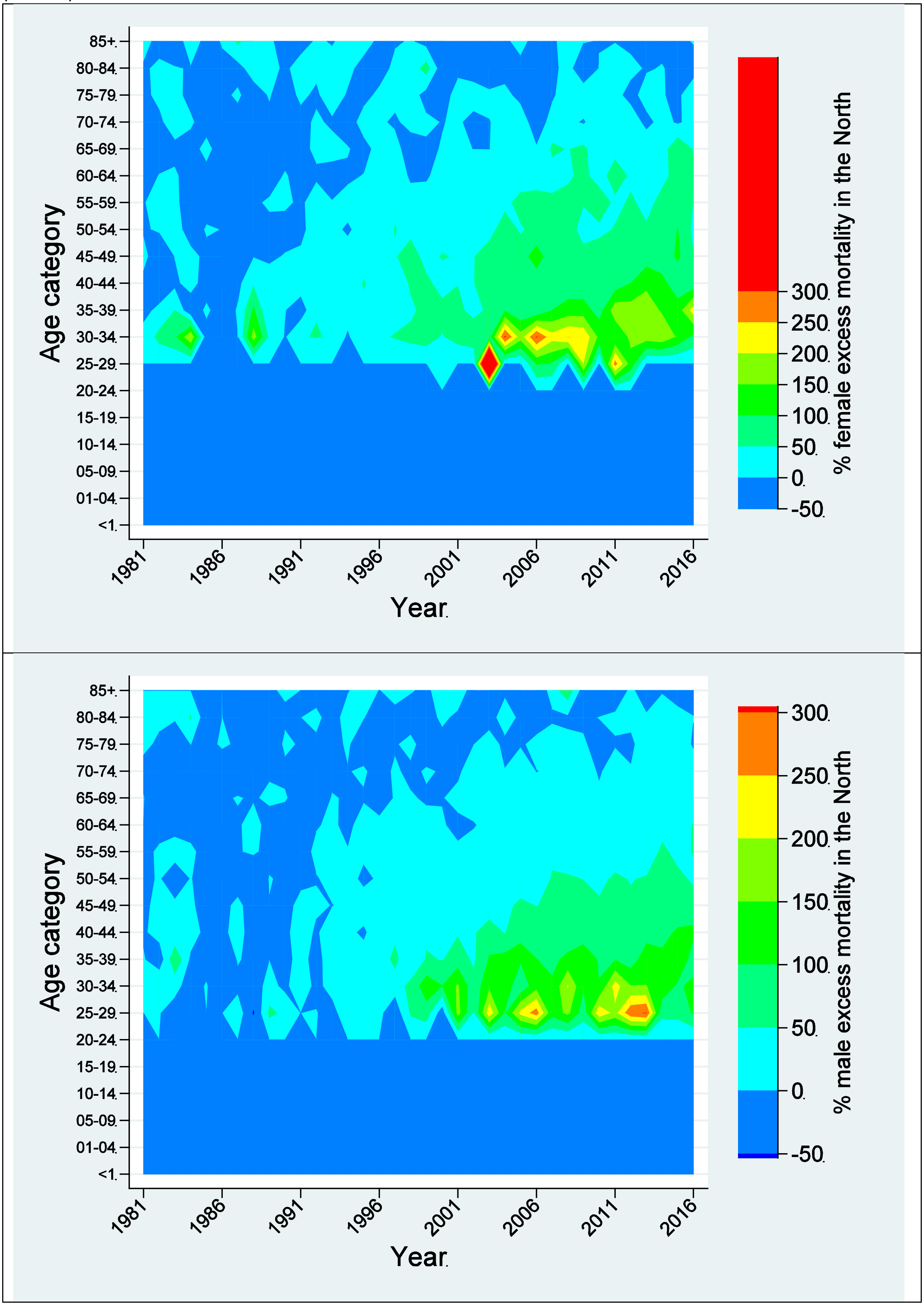

Figure S5: Cardiovascular related excess mortality in the North compared to the South, females (top) and males (bottom)*

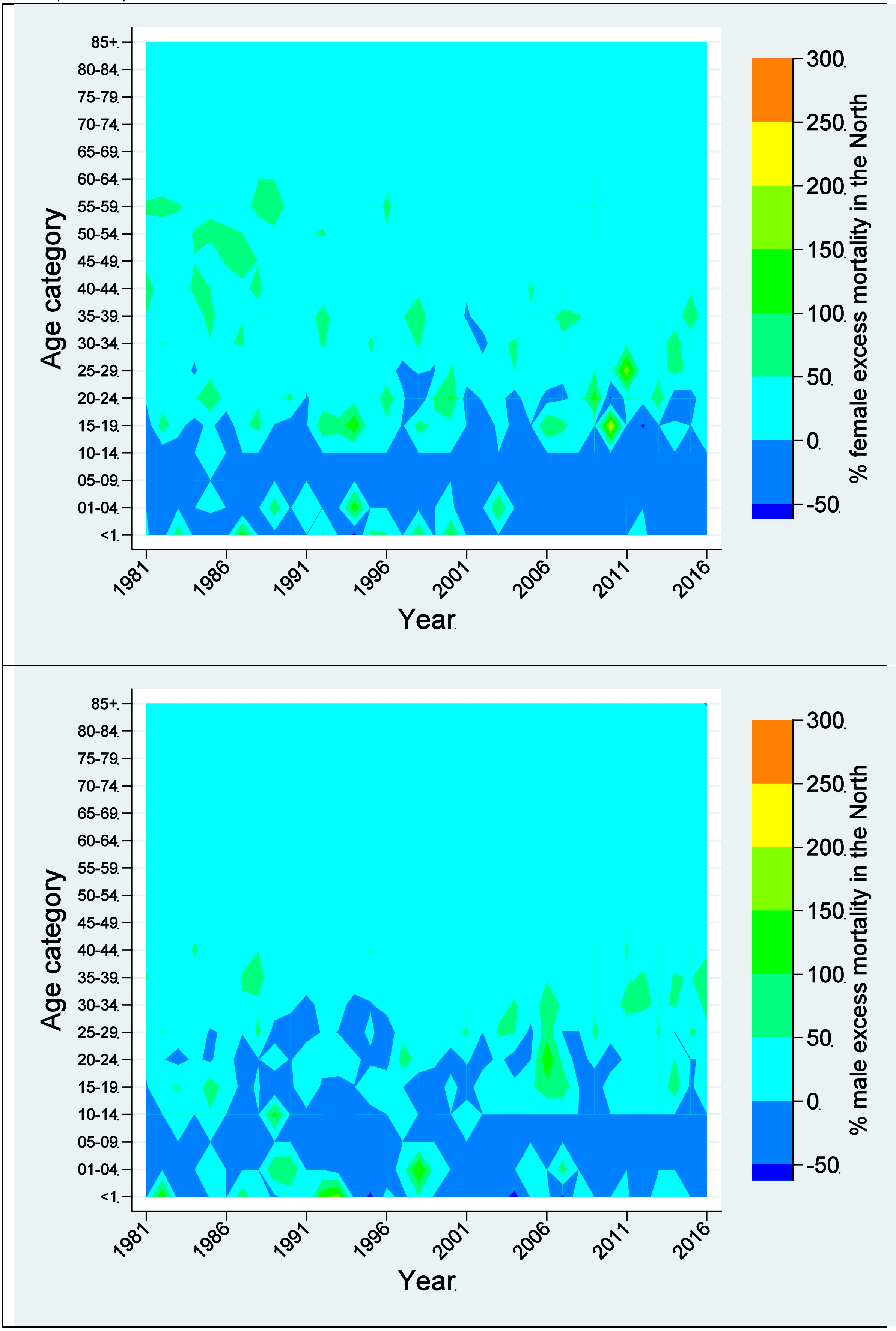


*Cardiovascular includes diabetes and obesity, with the overwhelming majority of deaths attributed to cardiovascular

Figure S6: Suicide related excess mortality in the North compared to the South, females (top) and males (bottom)

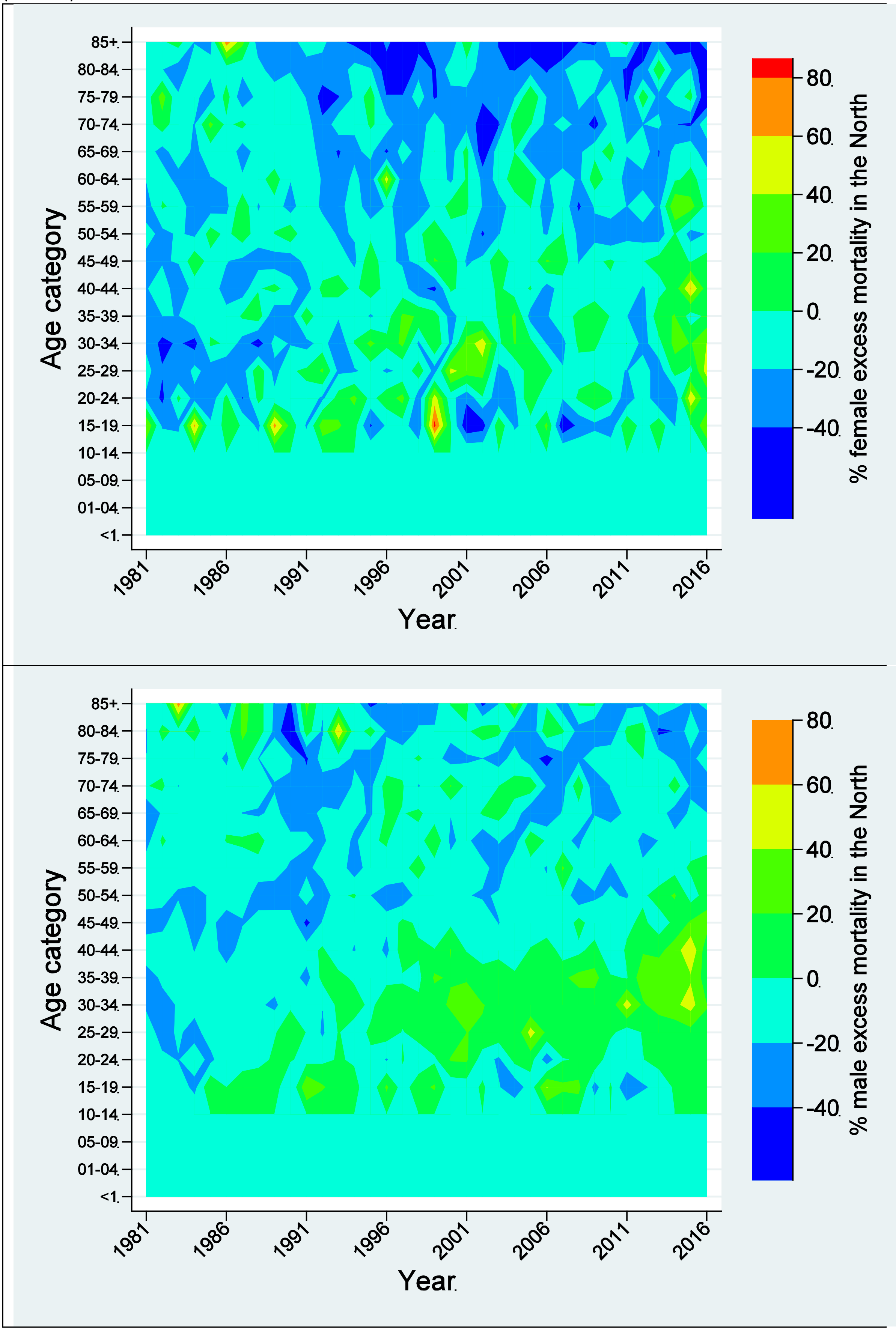

Figure S7: Drug related excess mortality in the North compared to the South, females (top) and males (bottom)

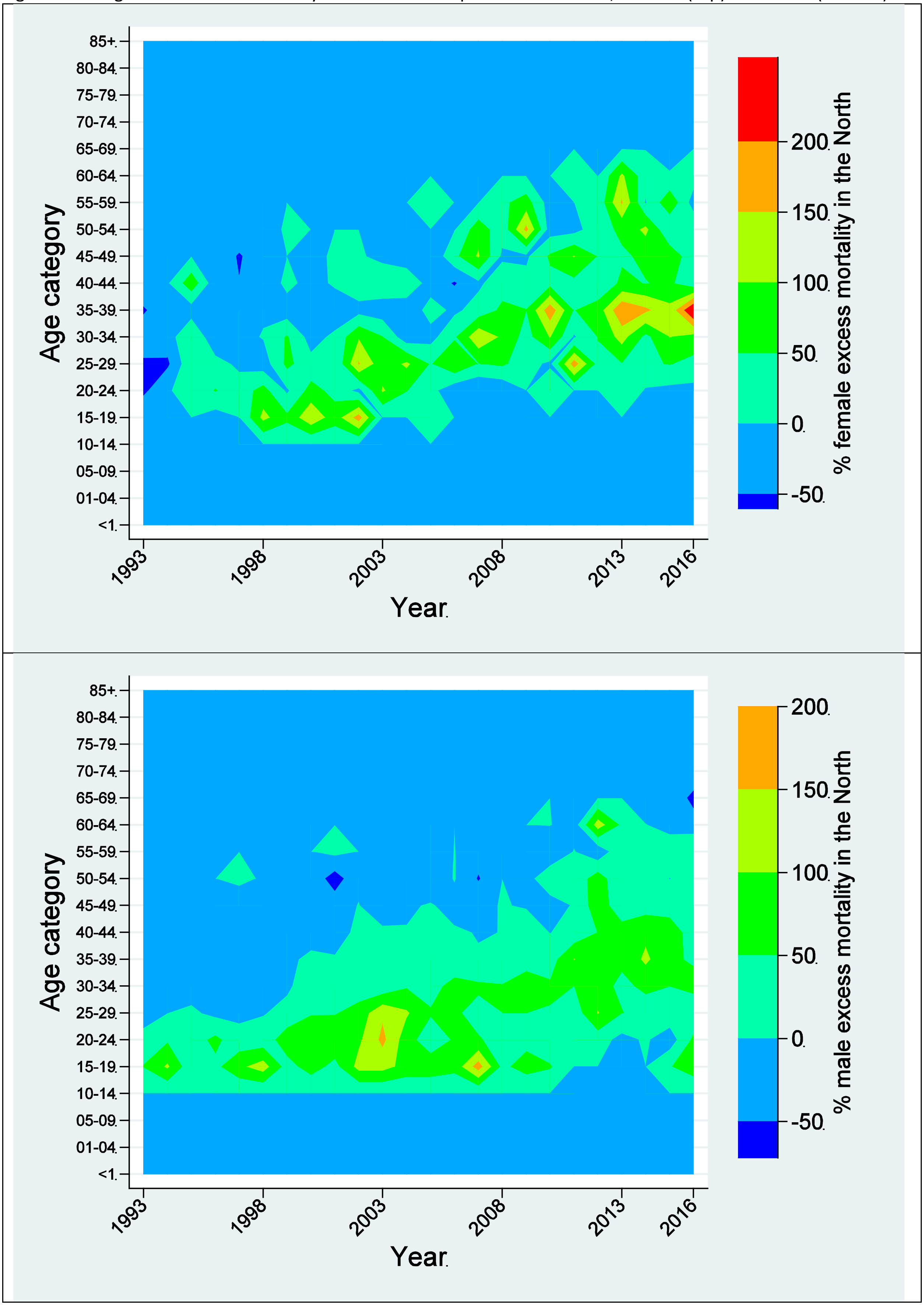

Figure S8: Cancer (excluding breast) related excess mortality in the North compared to the South, females (top) and males (bottom)

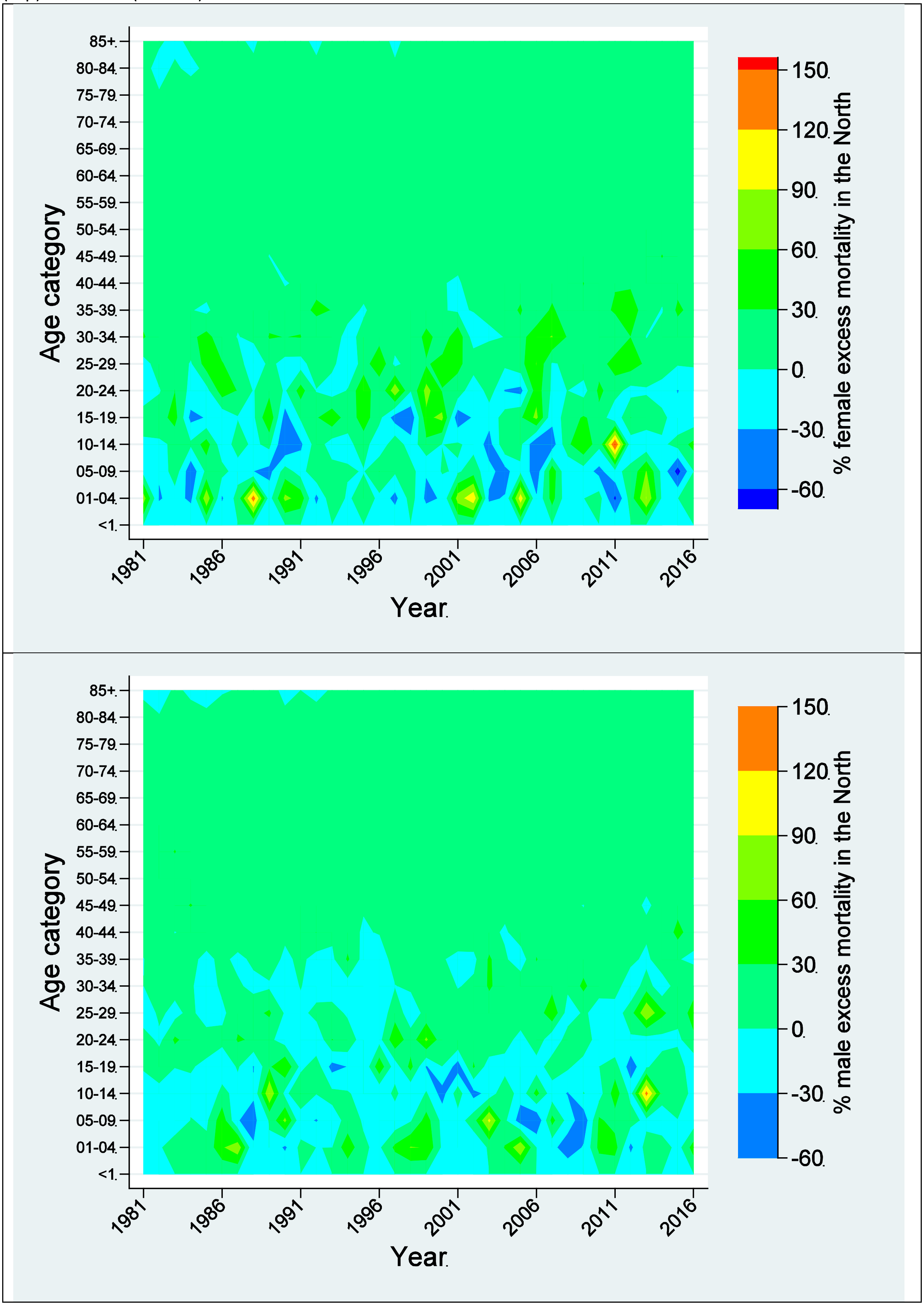

Figure S9: Breast cancer related excess mortality in the North compared to the South, females

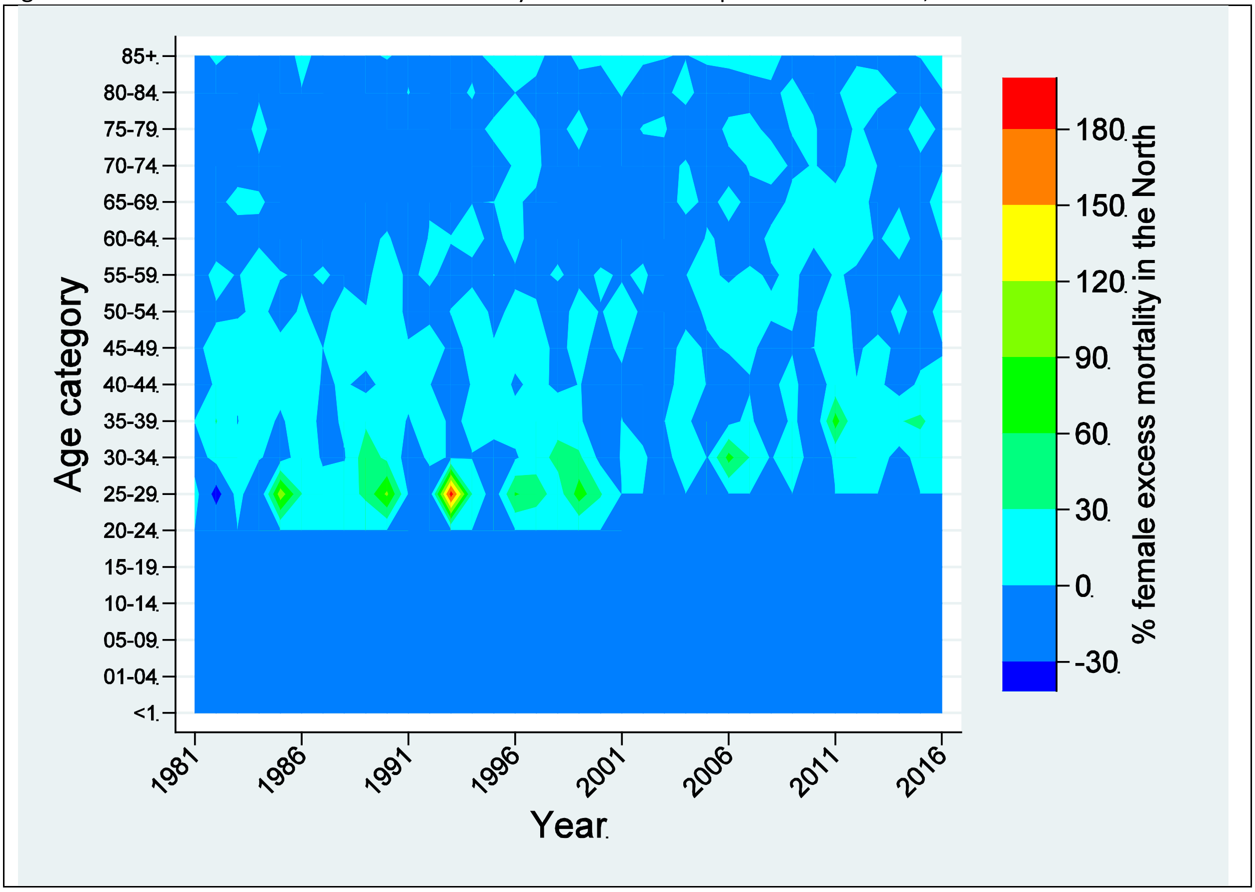

Figure S10: Other (not in the included underlying causes) excess mortality in the North compared to the South, females (top) and males (bottom)

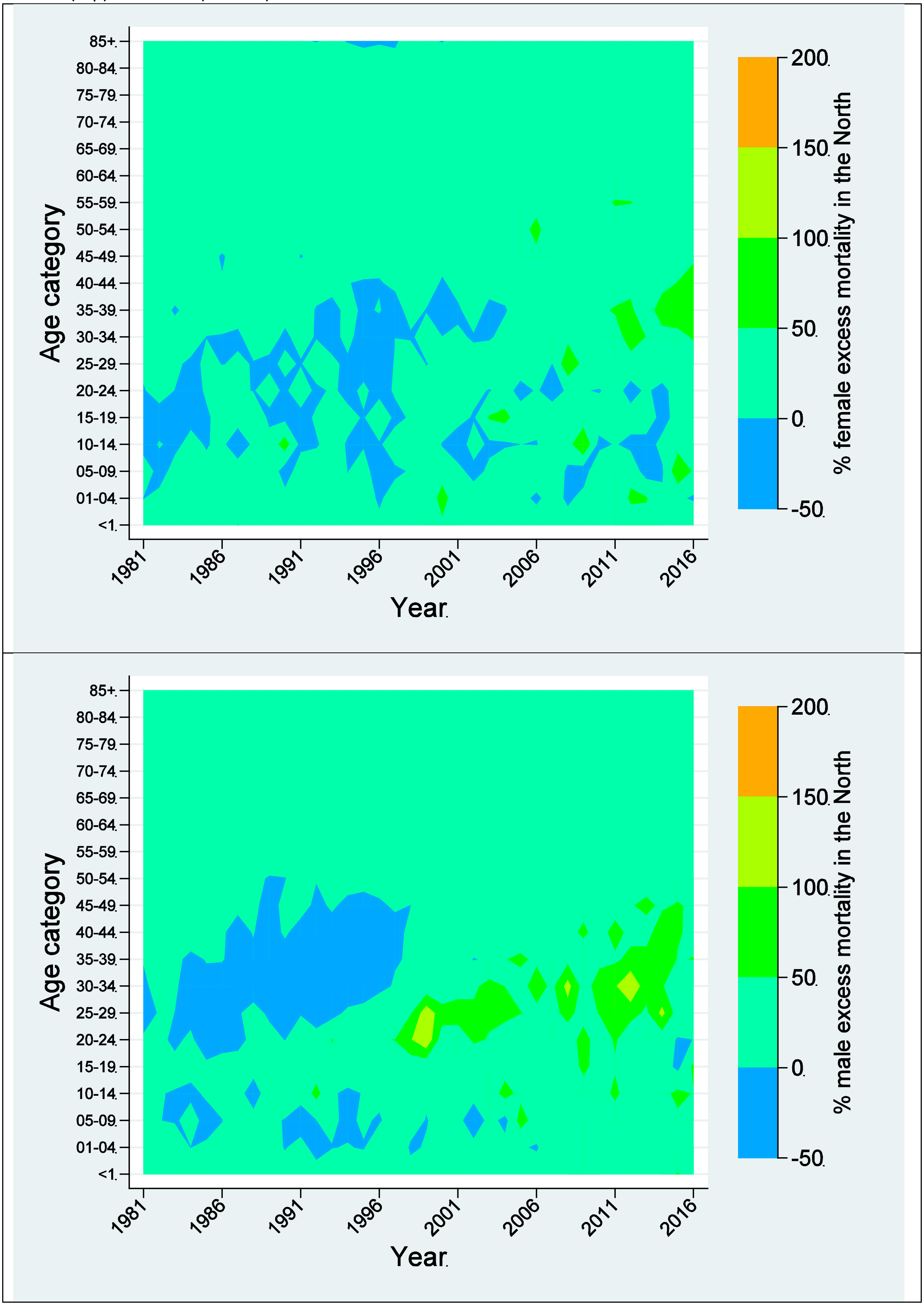

Figure S11: Percentage of excess deaths in the North attributed to each of the investigated underlying causes between 2014 and 2016, for females (top) and males (bottom)*

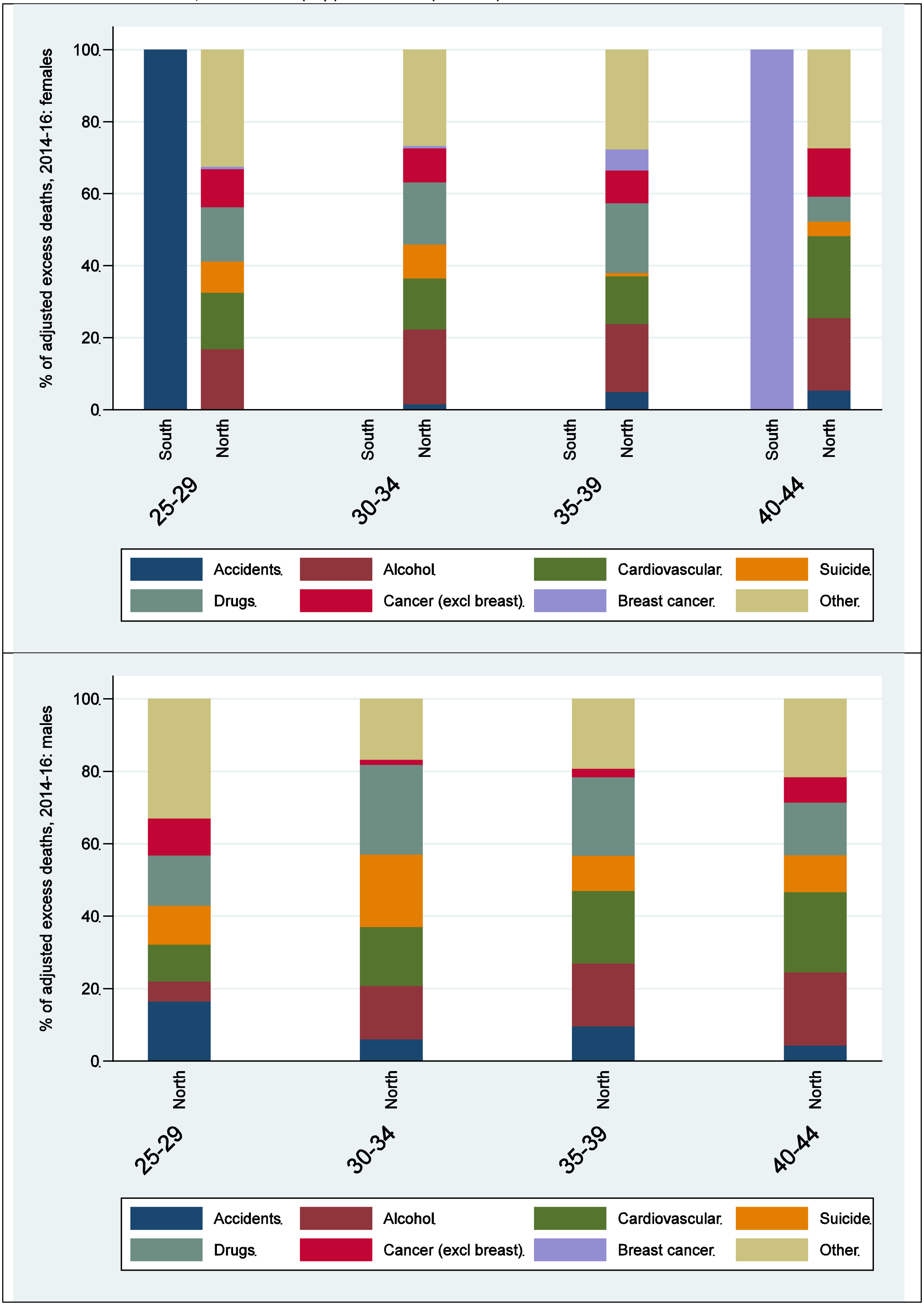


*Cardiovascular includes diabetes and obesity, with the overwhelming majority of deaths attributed to cardiovascular

Figure S12: Directly age-standardised accident-related mortality rates for those aged 25 to 44 in the North and the South of England from 1981 to 2016, females (top) and males (bottom)

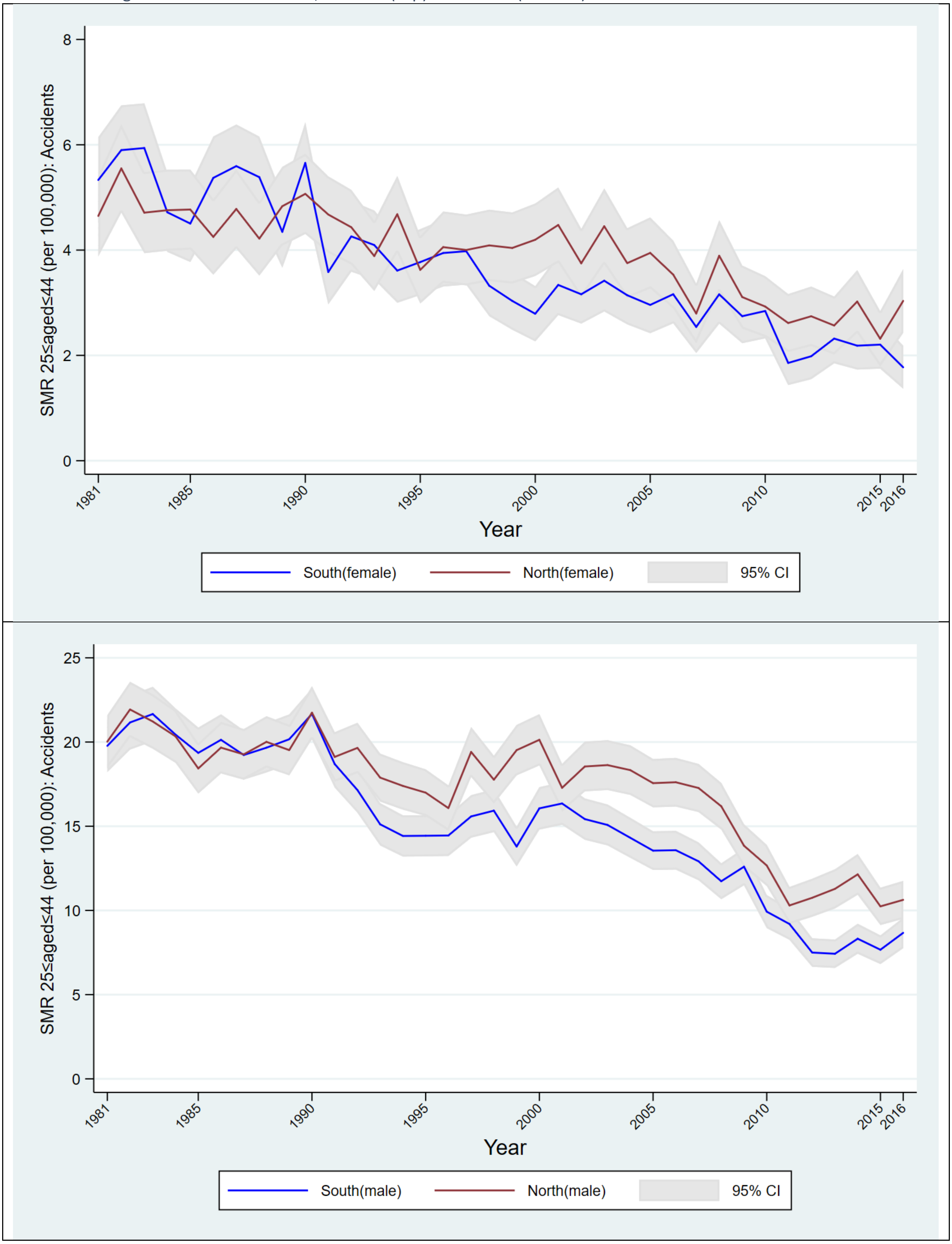

Figure S13: Directly age-standardised alcohol-related mortality rates for those aged 25 to 44 in the North and the South of England from 1981 to 2016, females (top) and males (bottom)

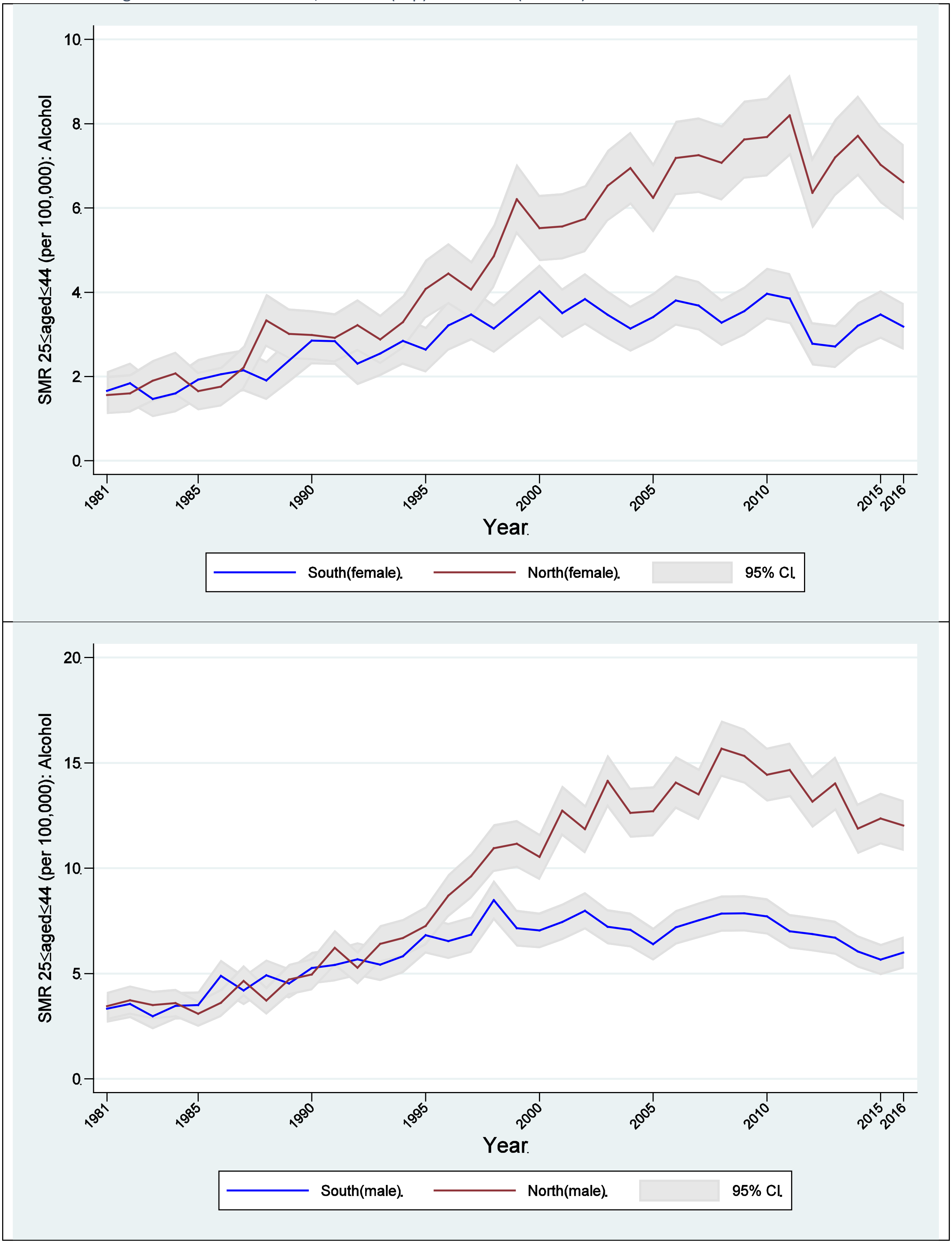

Figure S14: Directly age-standardised cardiovascular-related mortality rates for those aged 25 to 44 in the North and the South of England from 1981 to 2016, females (top) and males (bottom)*

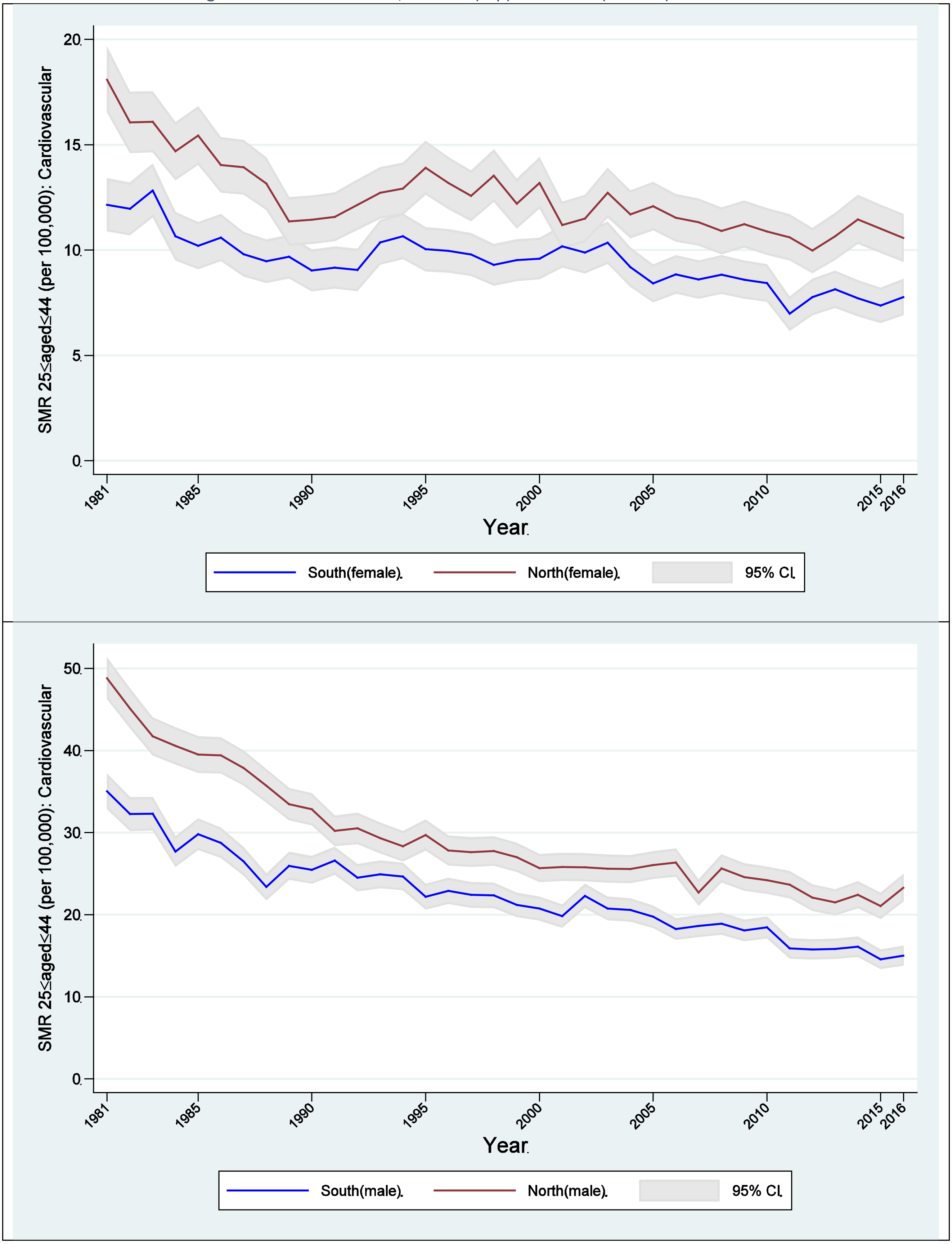


*Cardiovascular includes diabetes and obesity, with the overwhelming majority of deaths attributed to cardiovascular

Figure S15: Directly age-standardised suicide-related mortality rates for those aged 25 to 44 in the North and the South of England from 1981 to 2016, females (top) and males (bottom)

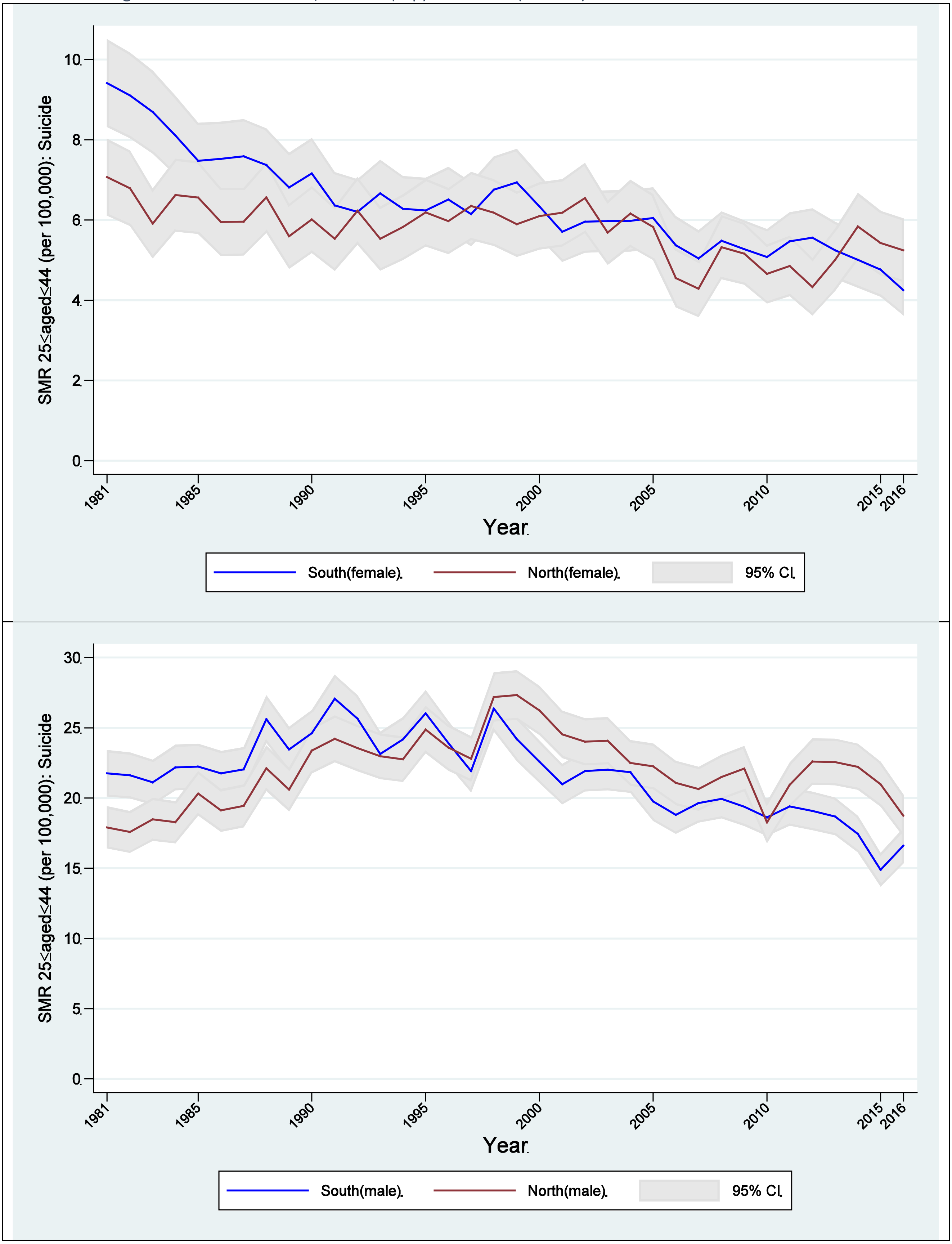

Figure S16: Directly age-standardised drug-related mortality rates for those aged 25 to 44 in the North and the South of England from 1993 to 2016, females (top) and males (bottom)

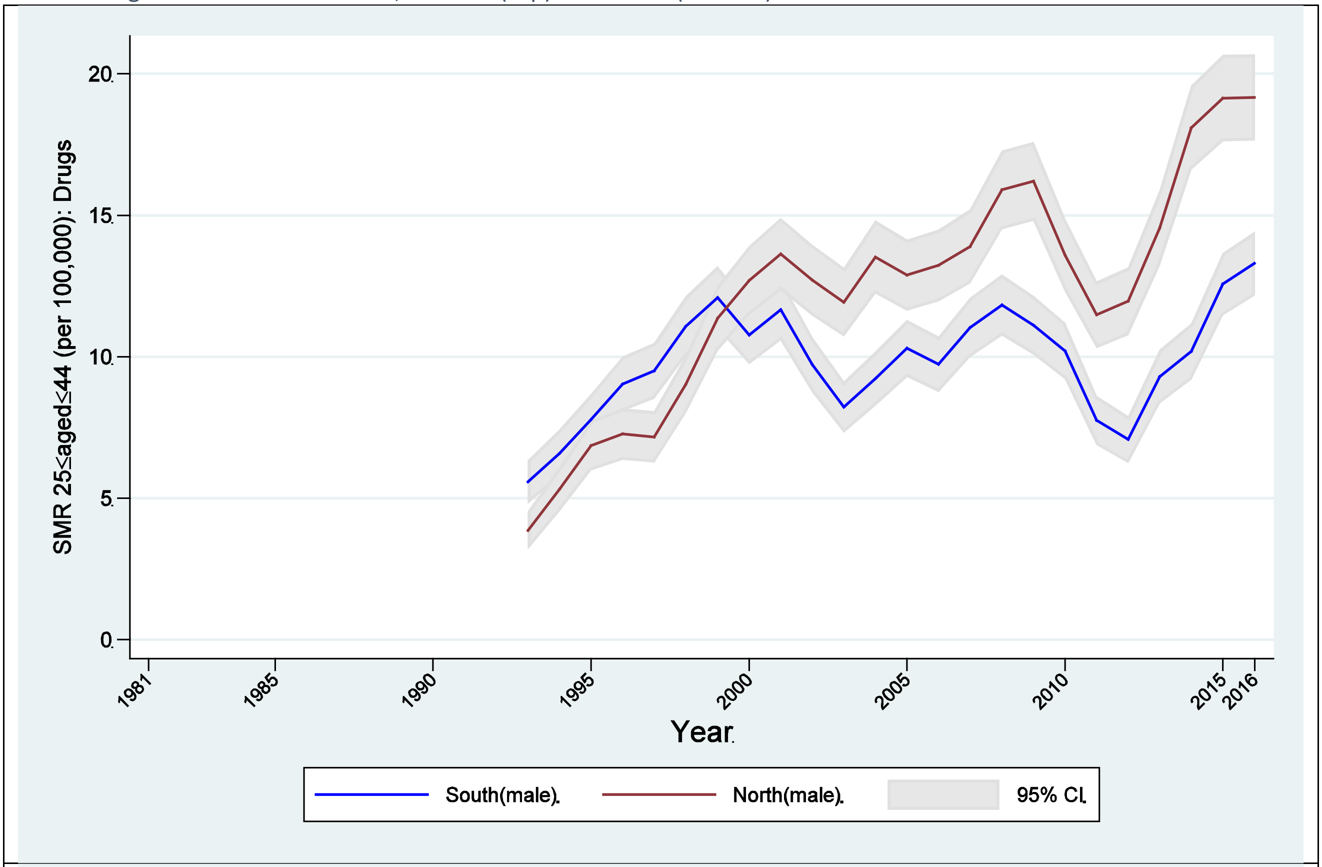


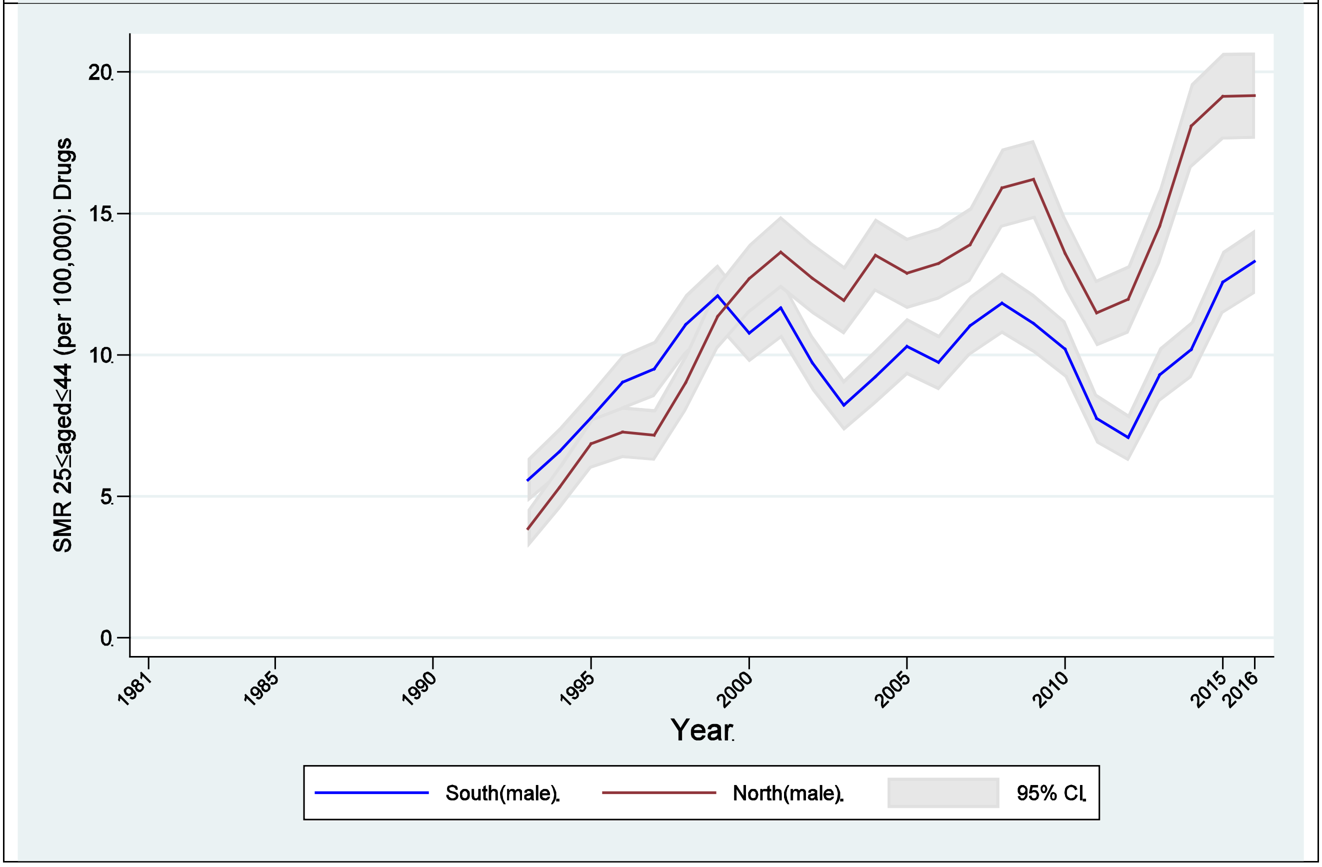

Figure S17: Directly age-standardised cancer-related (excluding breast cancer) mortality rates for those aged 25 to 44 in the North and the South of England from 1981 to 2016, females (top) and males (bottom)

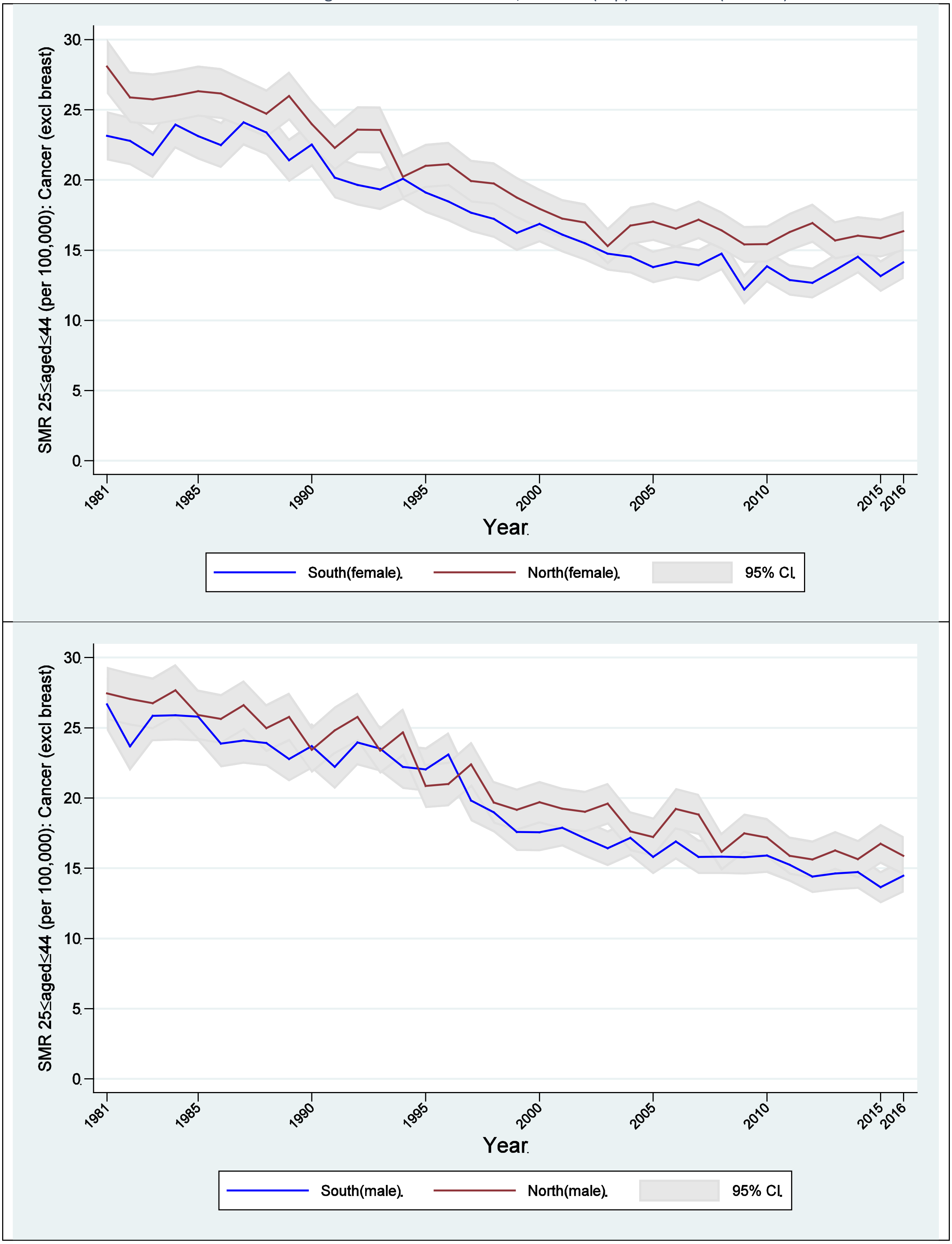

Figure S18: Directly age-standardised breast cancer-related mortality rates for those aged 25 to 44 in the North and the South of England from 1981 to 2016, females

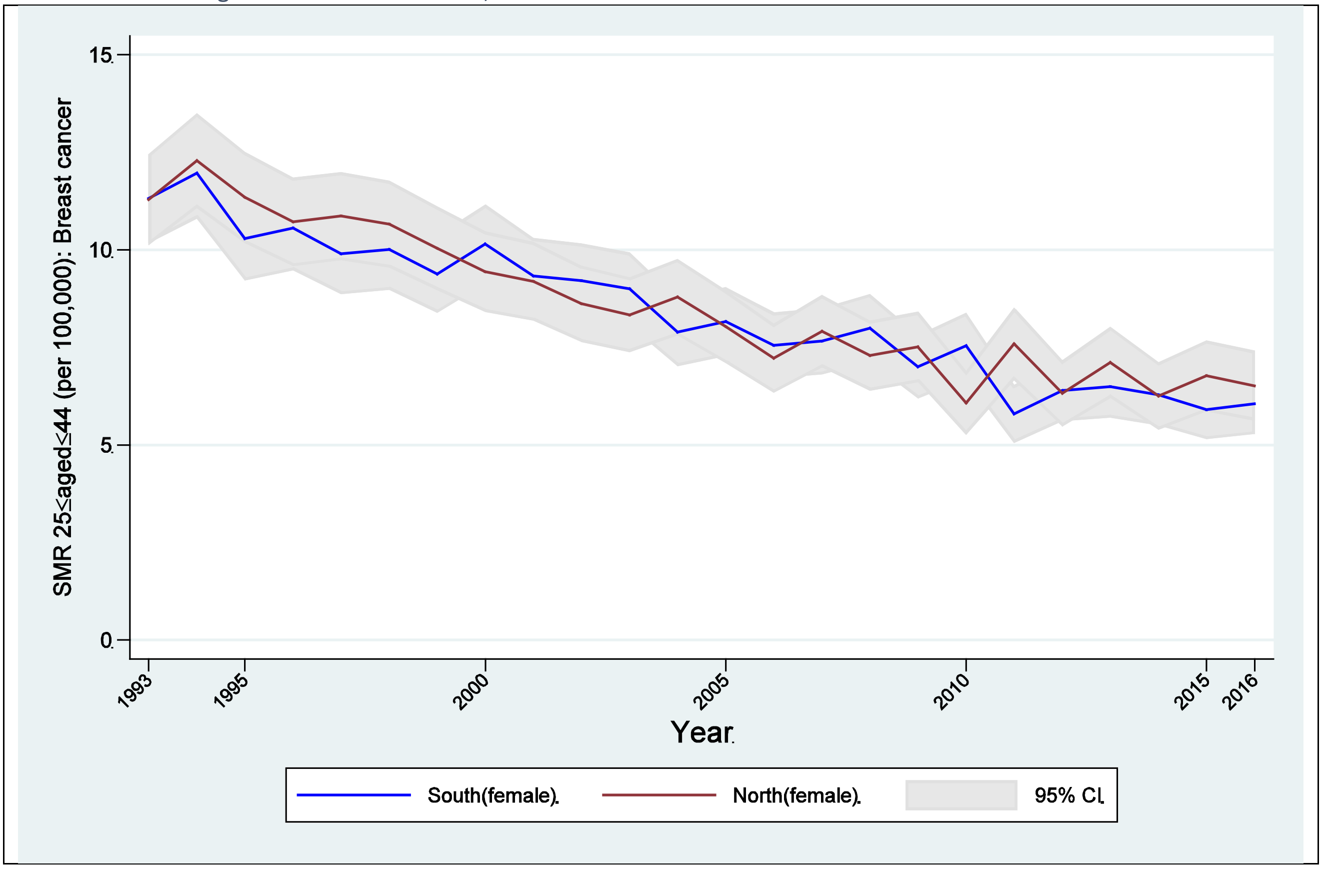

Figure S19: Directly age-standardised other (not in the included underlying causes) mortality rates for those aged 25 to 44 in the North and the South of England from 1981 to 2016, females (top) and males (bottom)

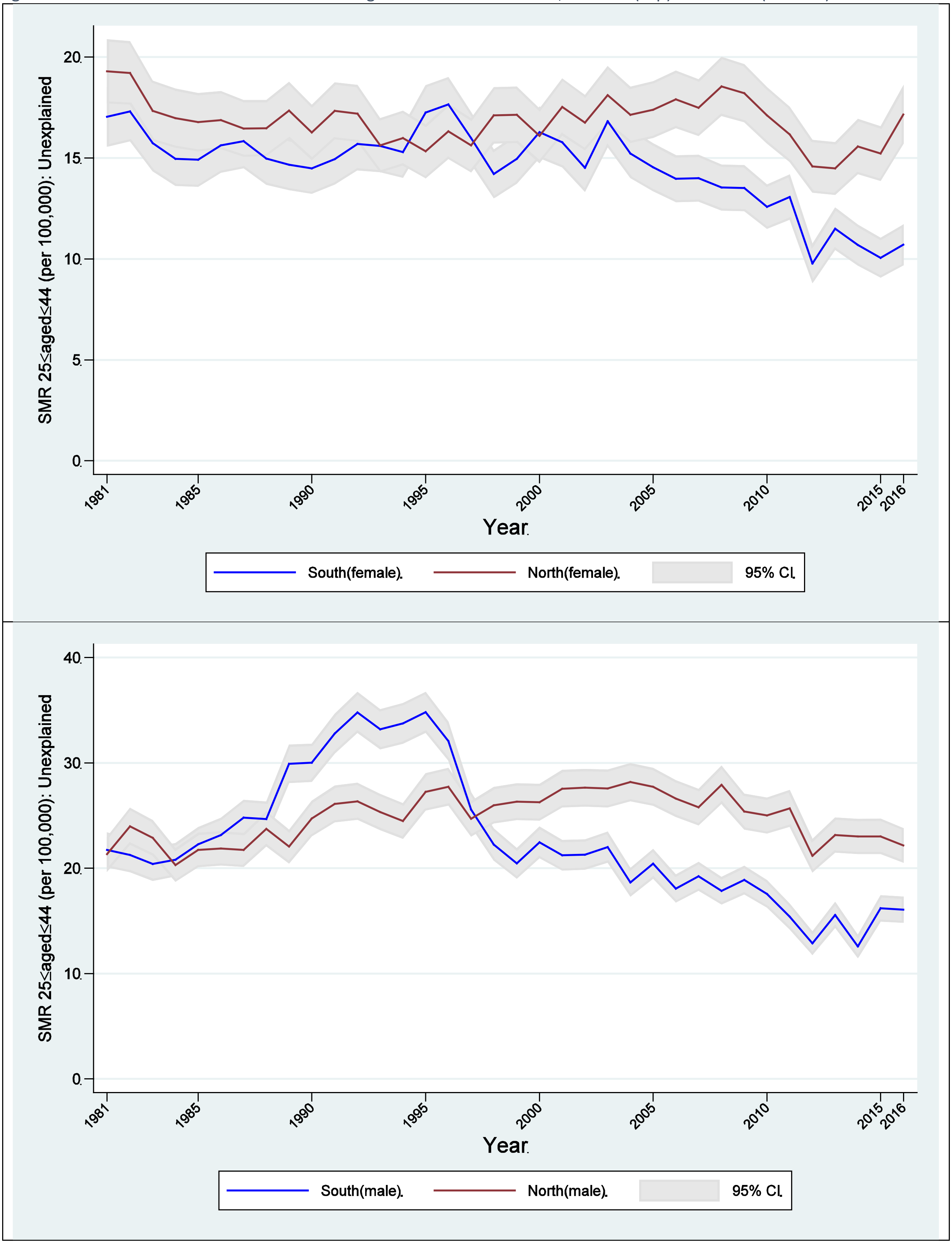

Figure S20: Directly age-standardised all-cause mortality rates for females aged 25 to 44 in 2016, at a low geographical level (LSOA) for the whole of England*

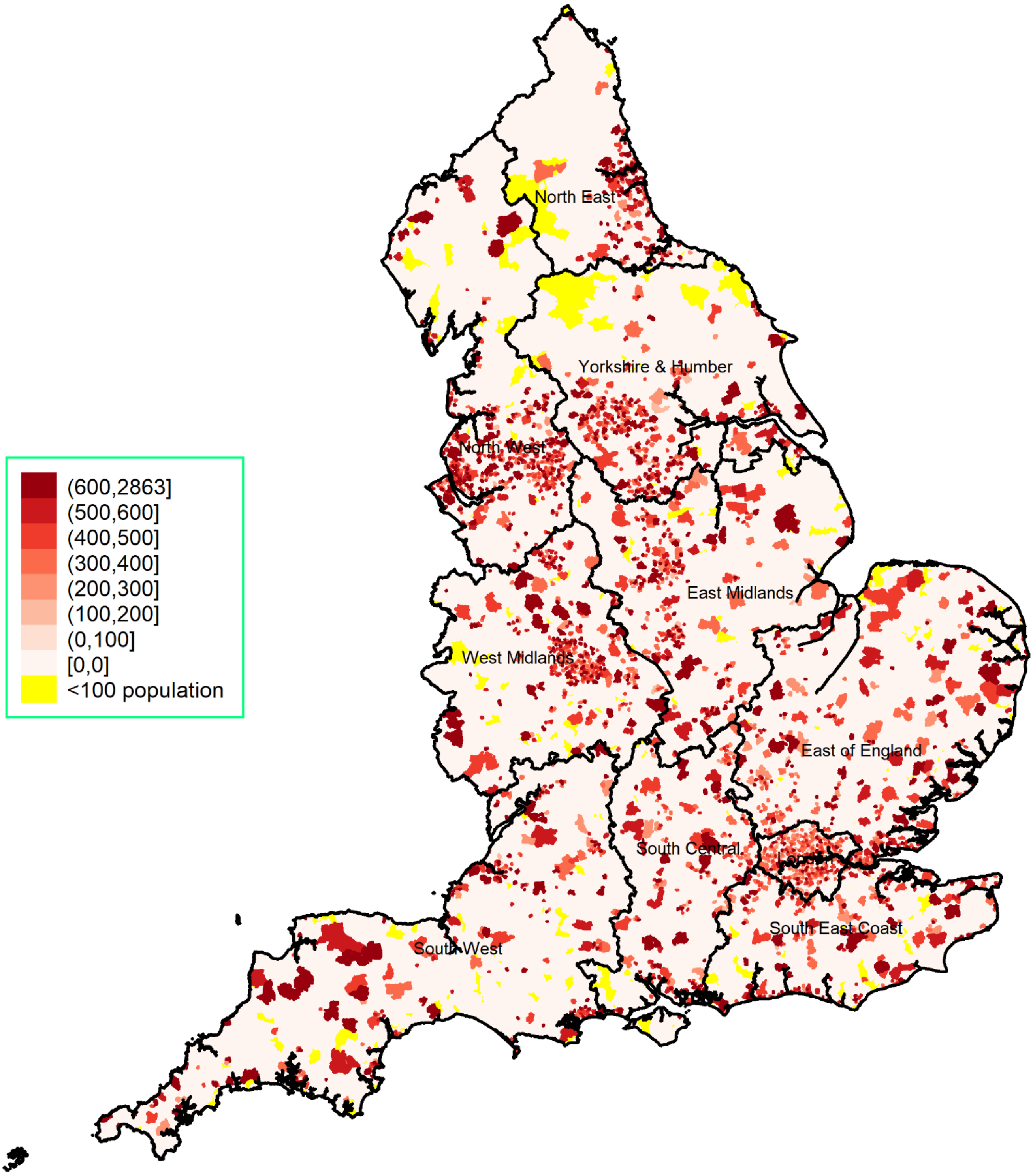


* LSOAs with fewer than 100 people aged 25-44 (i.e. "at risk") are supressed

Figure S21: Directly age-standardised all-cause mortality rates for males aged 25 to 44 in 2016, at a low geographical level (LSOA) for the whole of England*

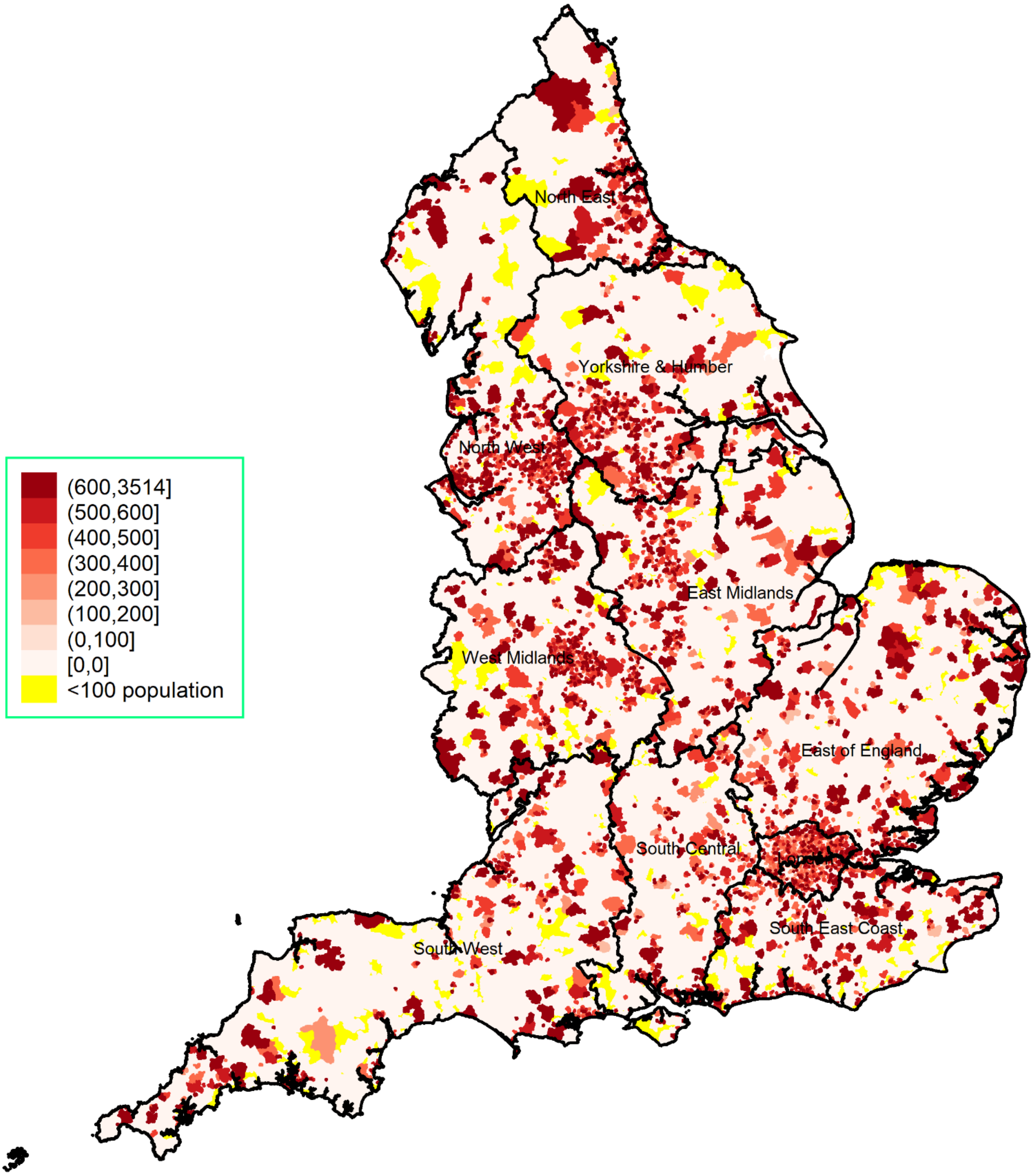


* LSOAs with fewer than 100 people aged 25-44 (i.e. "at risk") are supressed

Figure S22: Directly age-standardised all-cause mortality rates for people aged 25 to 44 in 2016, at a low geographical level (LSOA) for Greater London*

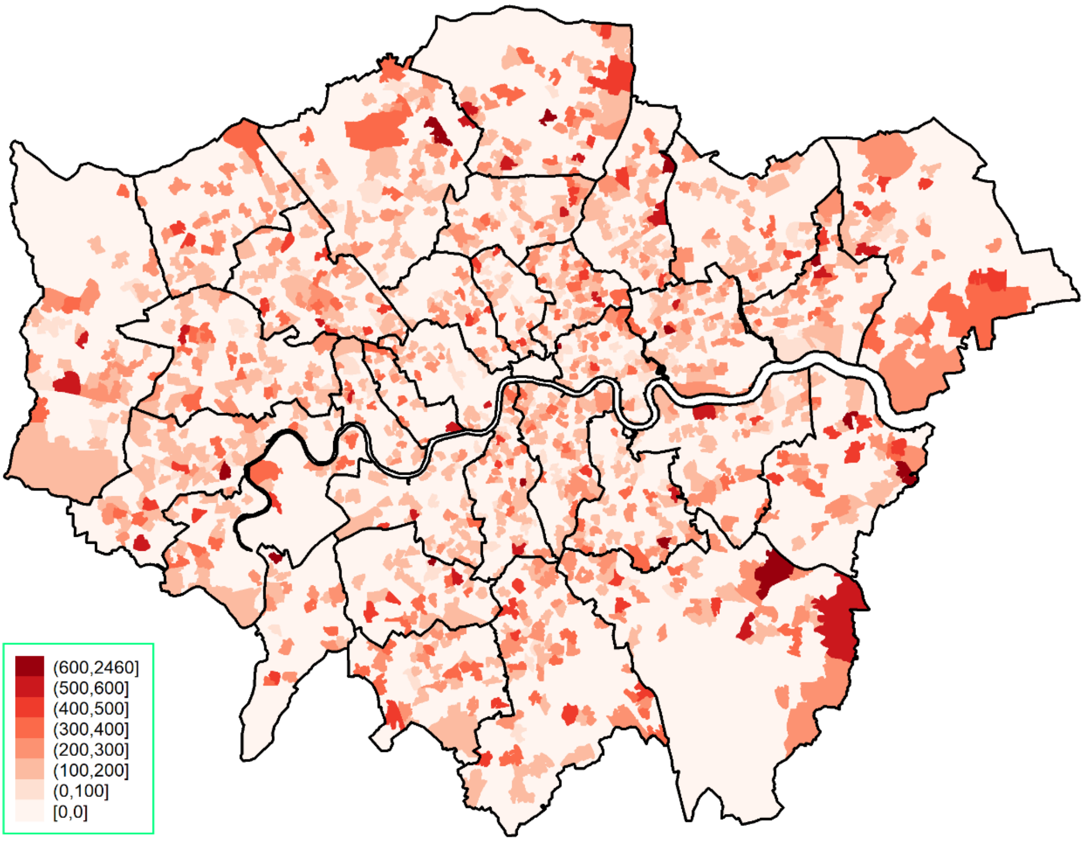


* LSOAs with fewer than 100 people aged 25-44 (i.e. “at risk”) are supressed

Figure S23: Directly age-standardised all-cause mortality rates for females aged 25 to 44 in 2016, at a low geographical level (LSOA) for Greater London*

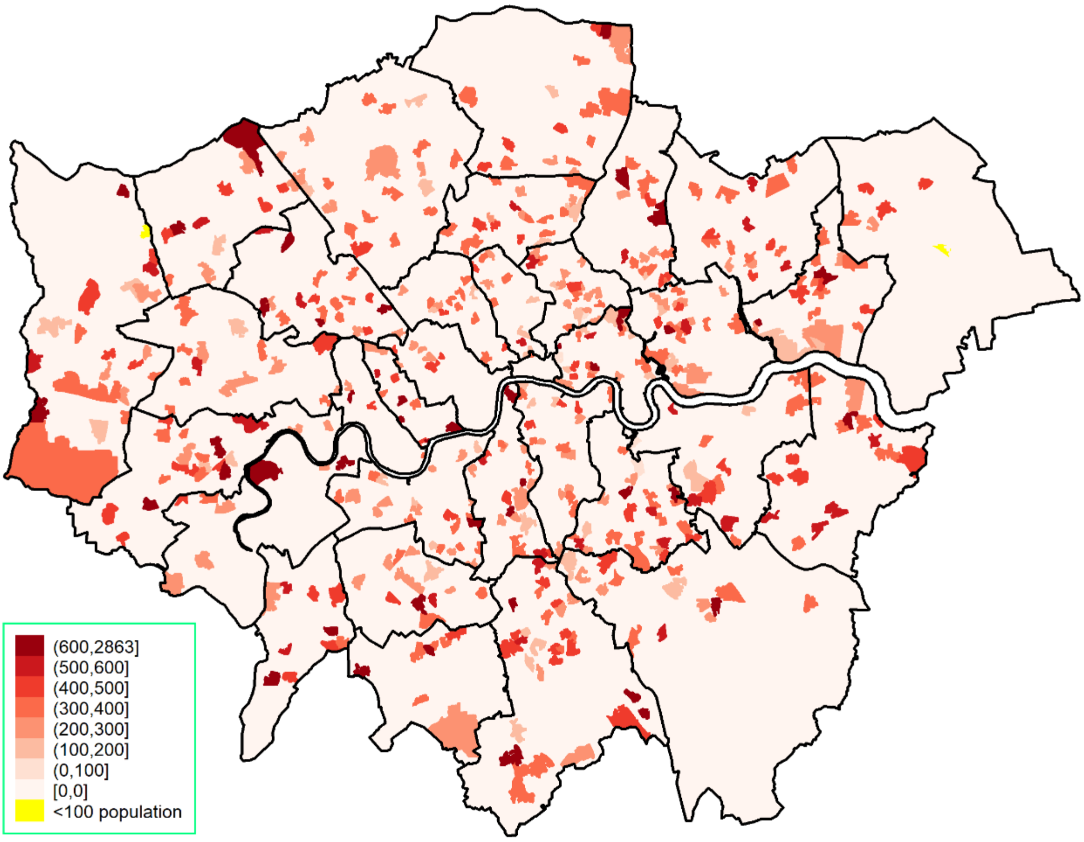


* LSOAs with fewer than 100 people aged 25-44 (i.e. “at risk”) are supressed

Figure S24: Directly age-standardised all-cause mortality rates for males aged 25 to 44 in 2016, at a low geographical level (LSOA) for Greater London*

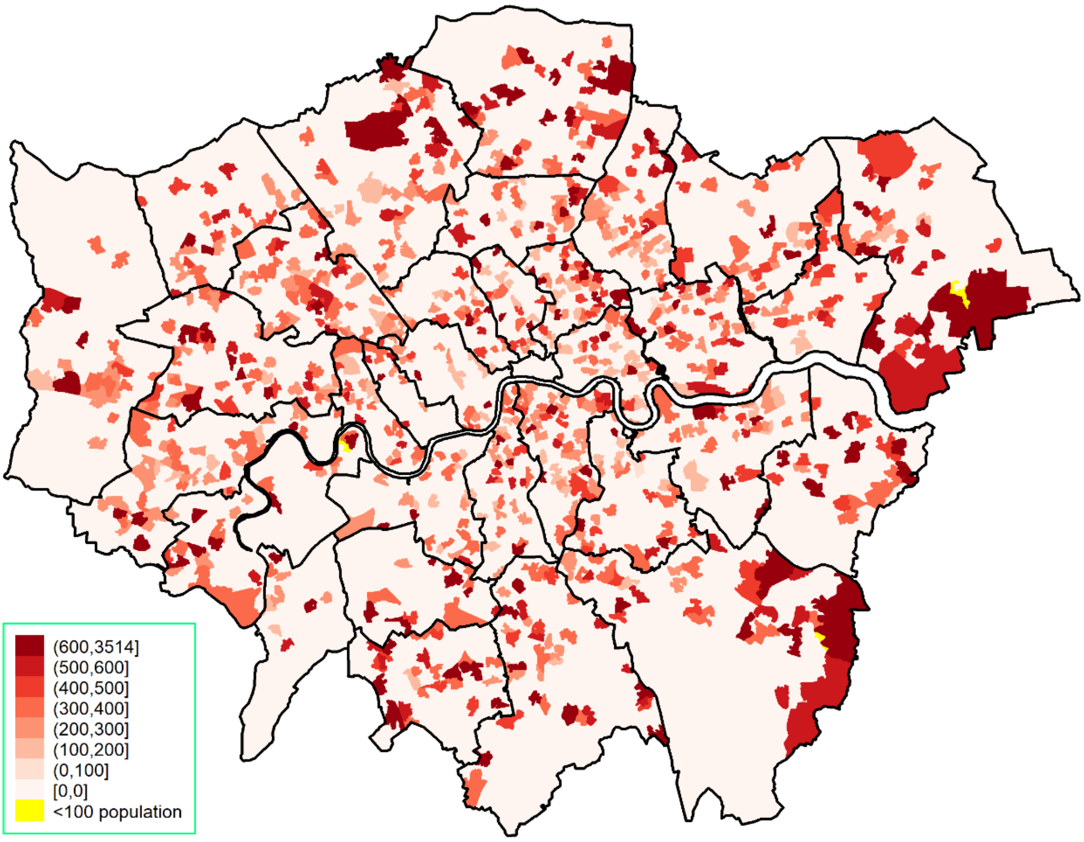


* LSOAs with fewer than 100 people aged 25-44 (i.e. "at risk") are supressed

Figure S25: Directly age-standardised all-cause mortality rates for people aged 25 to 44 in 2016, at a low geographical level (LSOA) for Greater Manchester*

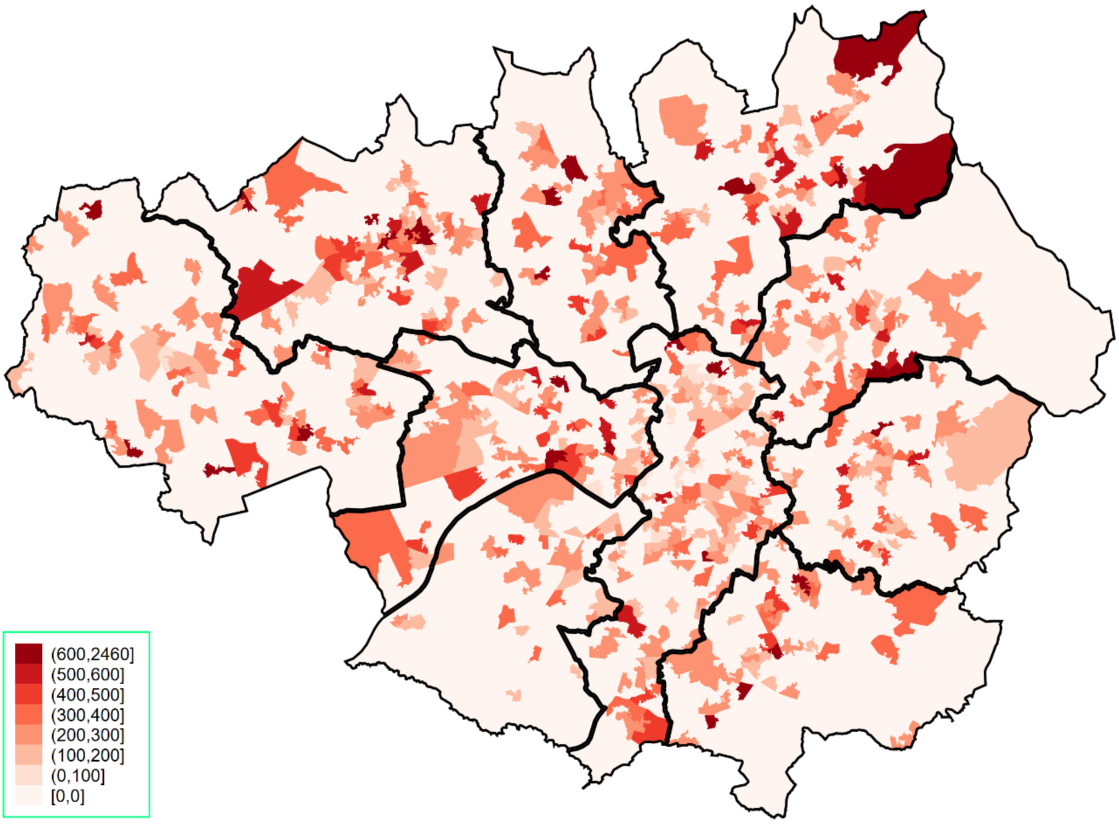


* LSOAs with fewer than 100 people aged 25-44 (i.e. "at risk") are supressed

Figure S26: Directly age-standardised all-cause mortality rates for females aged 25 to 44 in 2016, at a low geographical level (LSOA) for Greater Manchester*

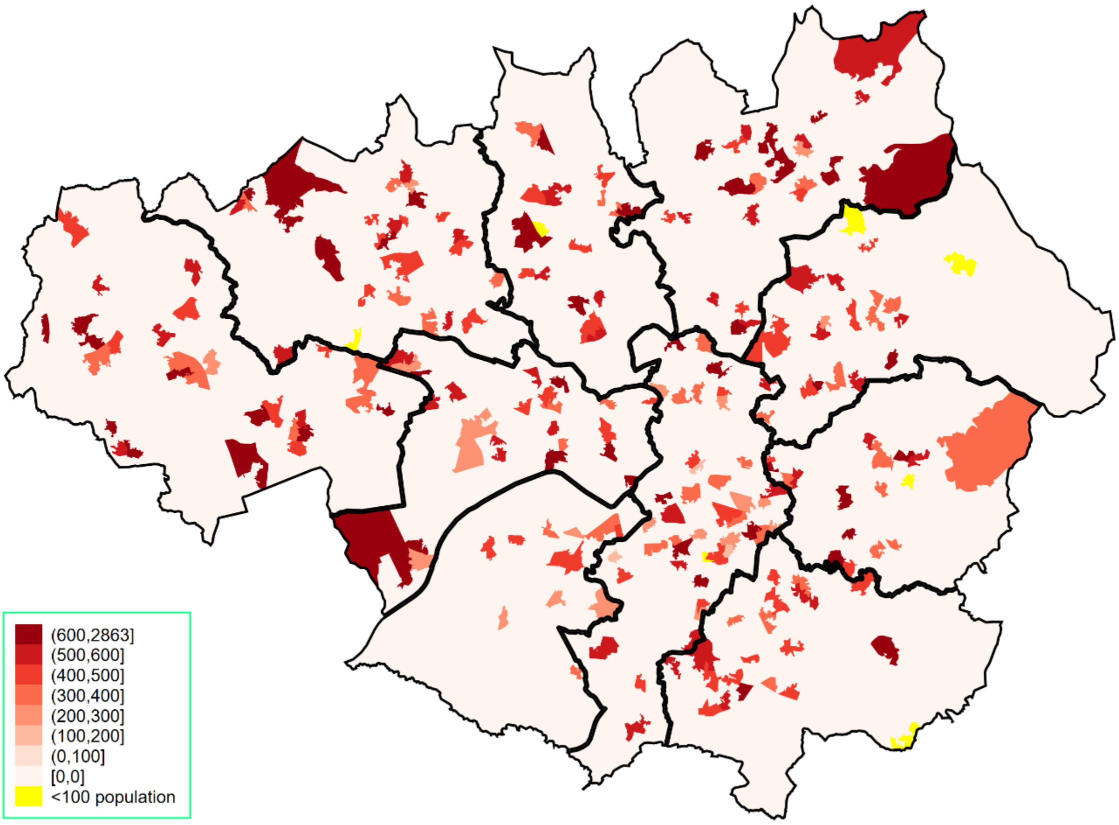


* LSOAs with fewer than 100 people aged 25-44 (i.e. "at risk") are supressed

Figure S27: Directly age-standardised all-cause mortality rates for males aged 25 to 44 in 2016, at a low geographical level (LSOA) for Greater Manchester*

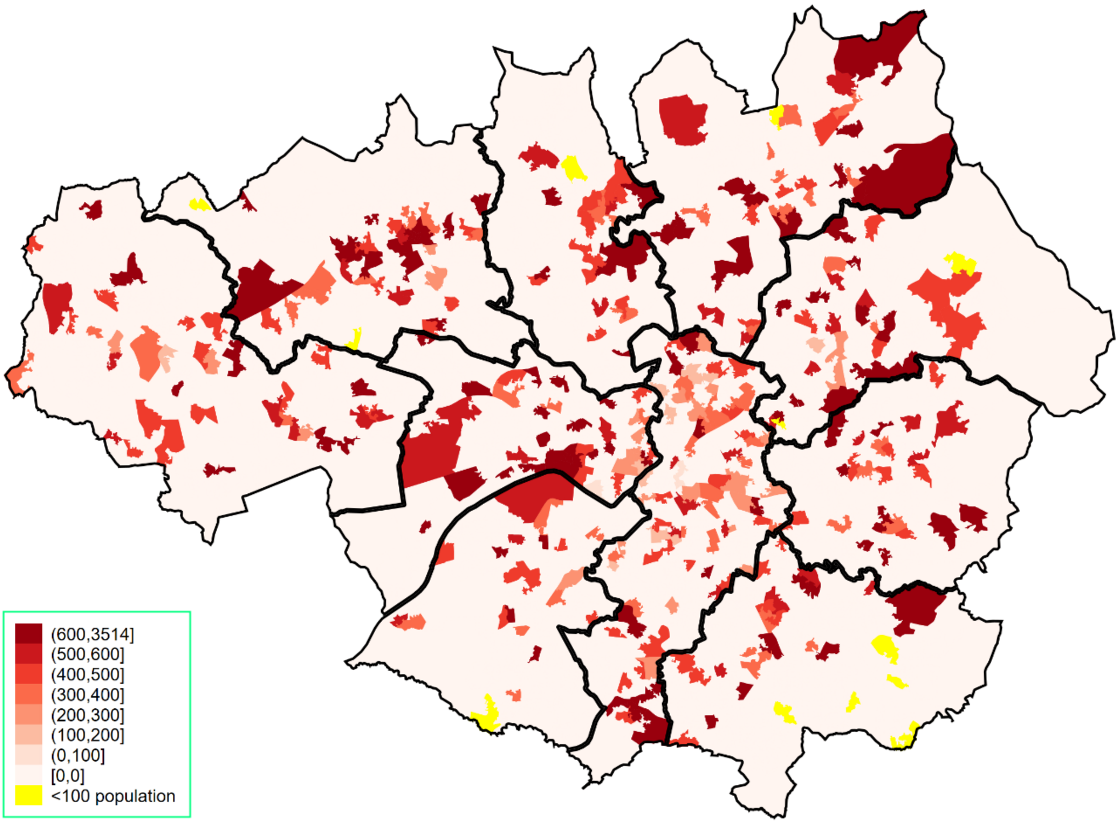


* LSOAs with fewer than 100 people aged 25-44 (i.e. “at risk”) are supressed

Figure S28: Directly age-standardised all-cause mortality rates for people aged 25 to 44 in 2016, at a low geographical level (LSOA) for the West Midlands conurbation (Birmingham)*

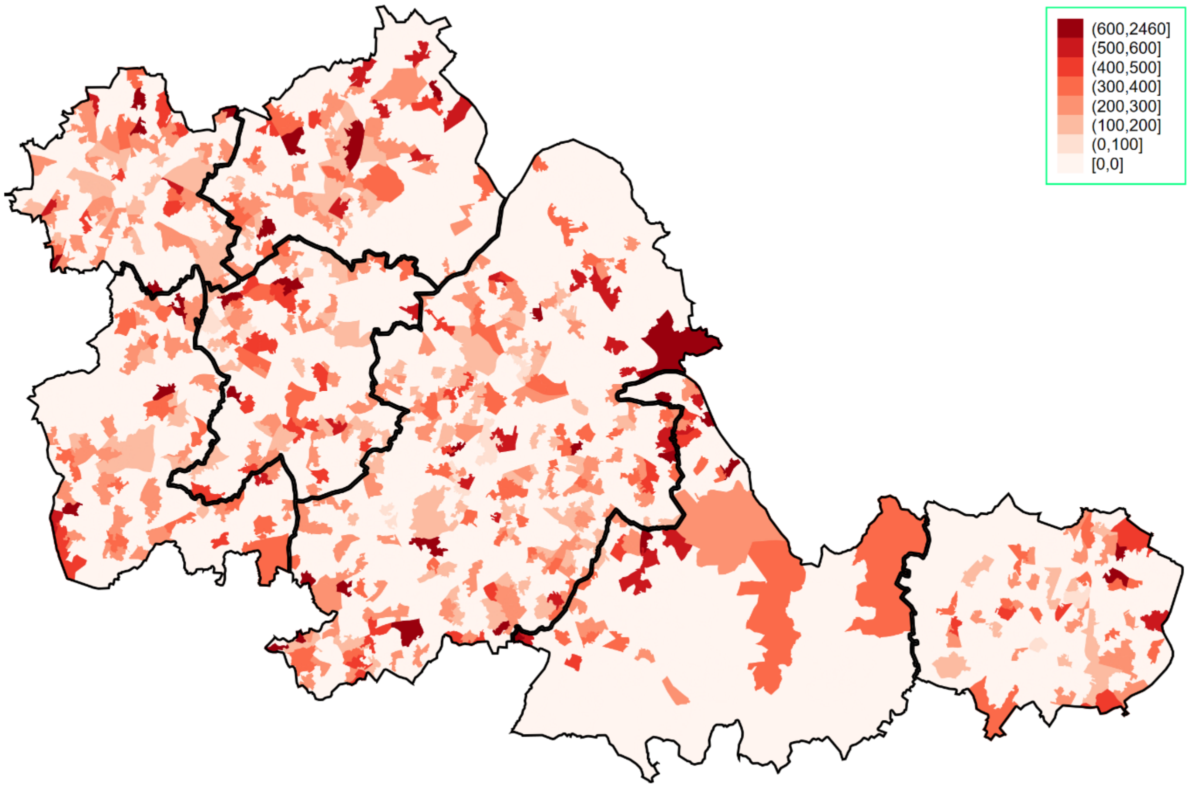


* LSOAs with fewer than 100 people aged 25-44 (i.e. "at risk") are supressed

Figure S29: Directly age-standardised all-cause mortality rates for females aged 25 to 44 in 2016, at a low geographical level (LSOA) for the West Midlands conurbation (Birmingham)*

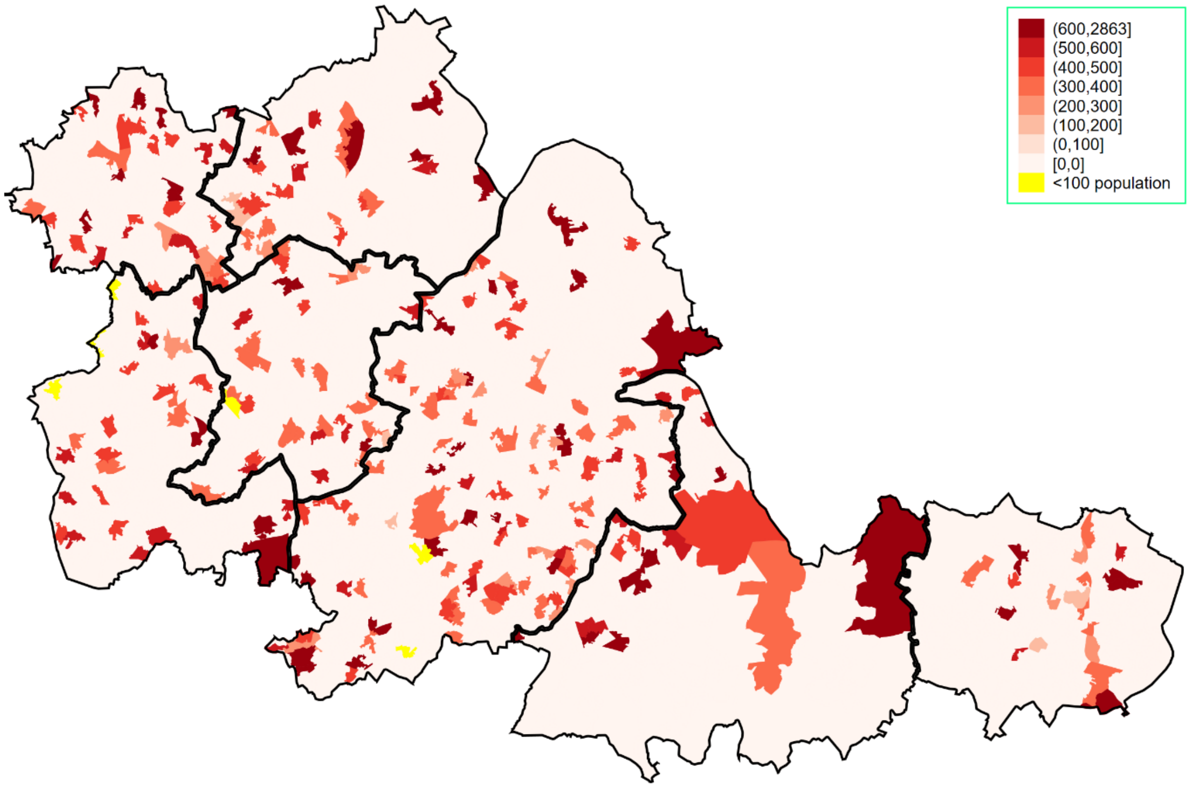


* LSOAs with fewer than 100 people aged 25-44 (i.e. "at risk") are supressed

Figure S30: Directly age-standardised all-cause mortality rates for males aged 25 to 44 in 2016, at a low geographical level (LSOA) for the West Midlands conurbation (Birmingham)*

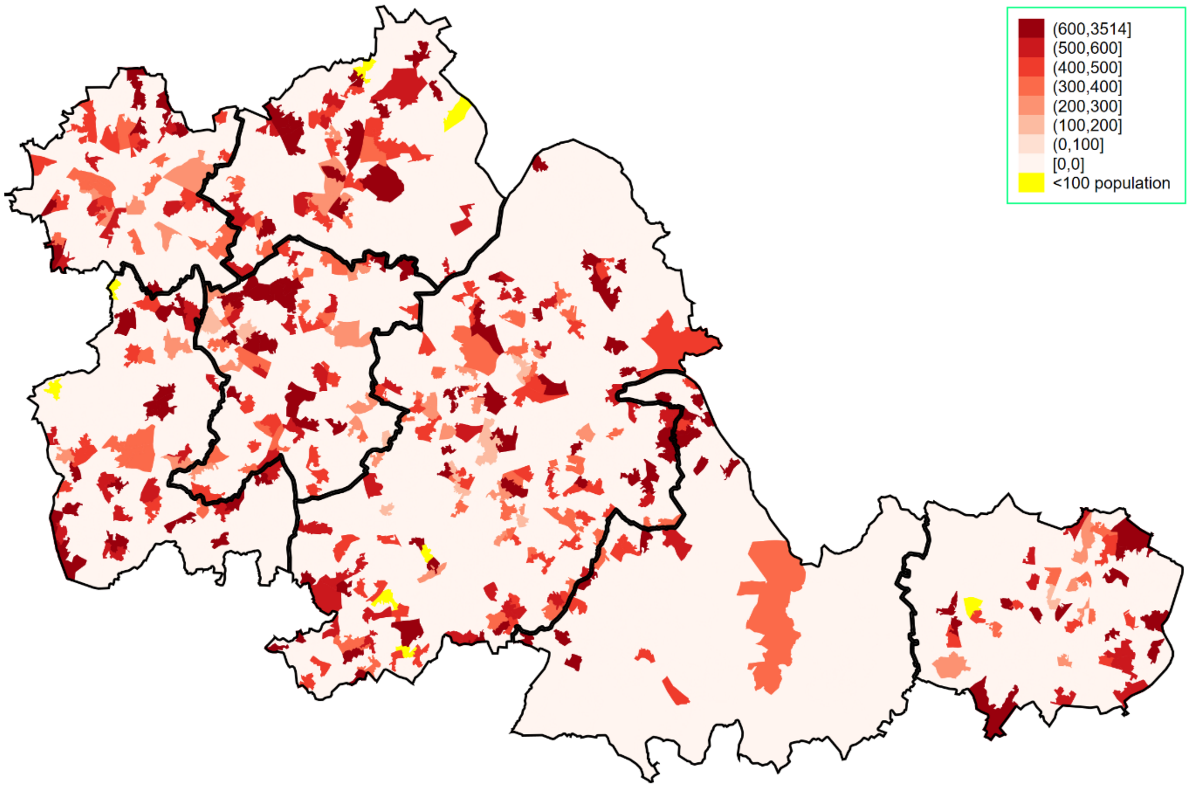


* LSOAs with fewer than 100 people aged 25-44 (i.e. “at risk”) are supressed

Figure S31: Directly age-standardised all-cause mortality rates for people aged 25 to 44 in 2016, at a low geographical level (LSOA) for East Midlands*

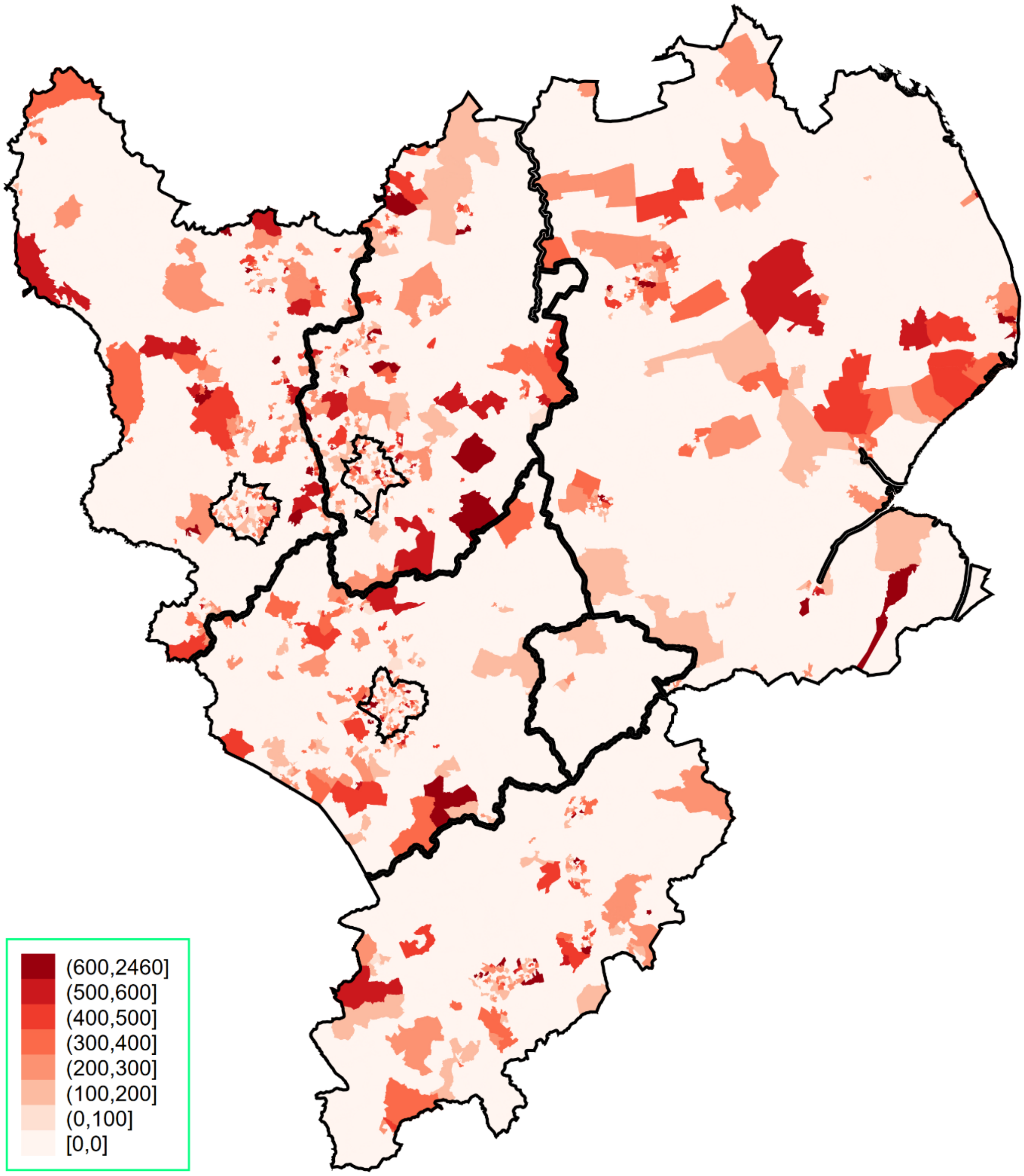


* LSOAs with fewer than 100 people aged 25-44 (i.e. "at risk") are supressed

Figure S32: Directly age-standardised all-cause mortality rates for females aged 25 to 44 in 2016, at a low geographical level (LSOA) for East Midlands*

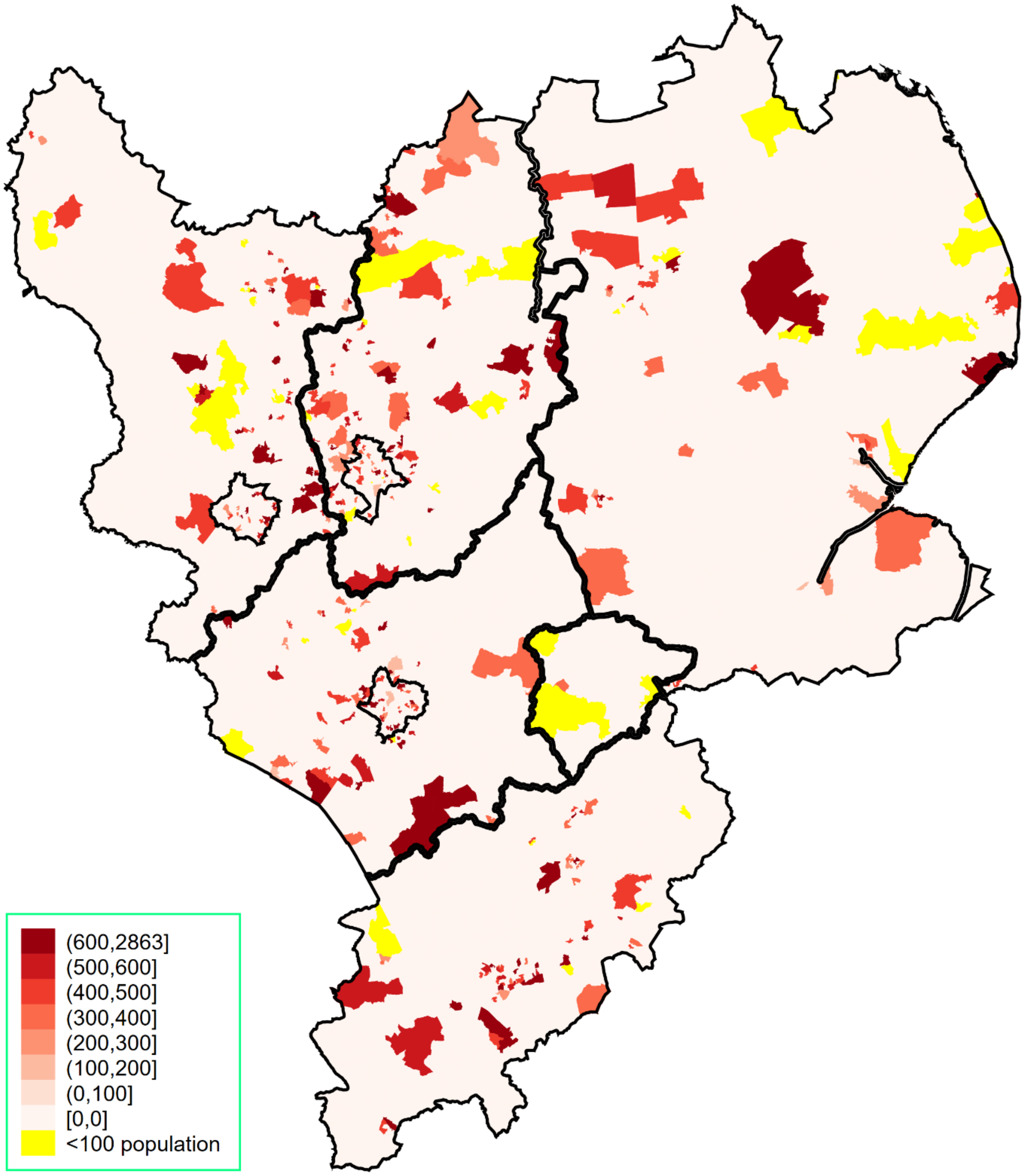


* LSOAs with fewer than 100 people aged 25-44 (i.e. "at risk") are supressed

Figure S33: Directly age-standardised all-cause mortality rates for males aged 25 to 44 in 2016, at a low geographical level (LSOA) for East Midlands*

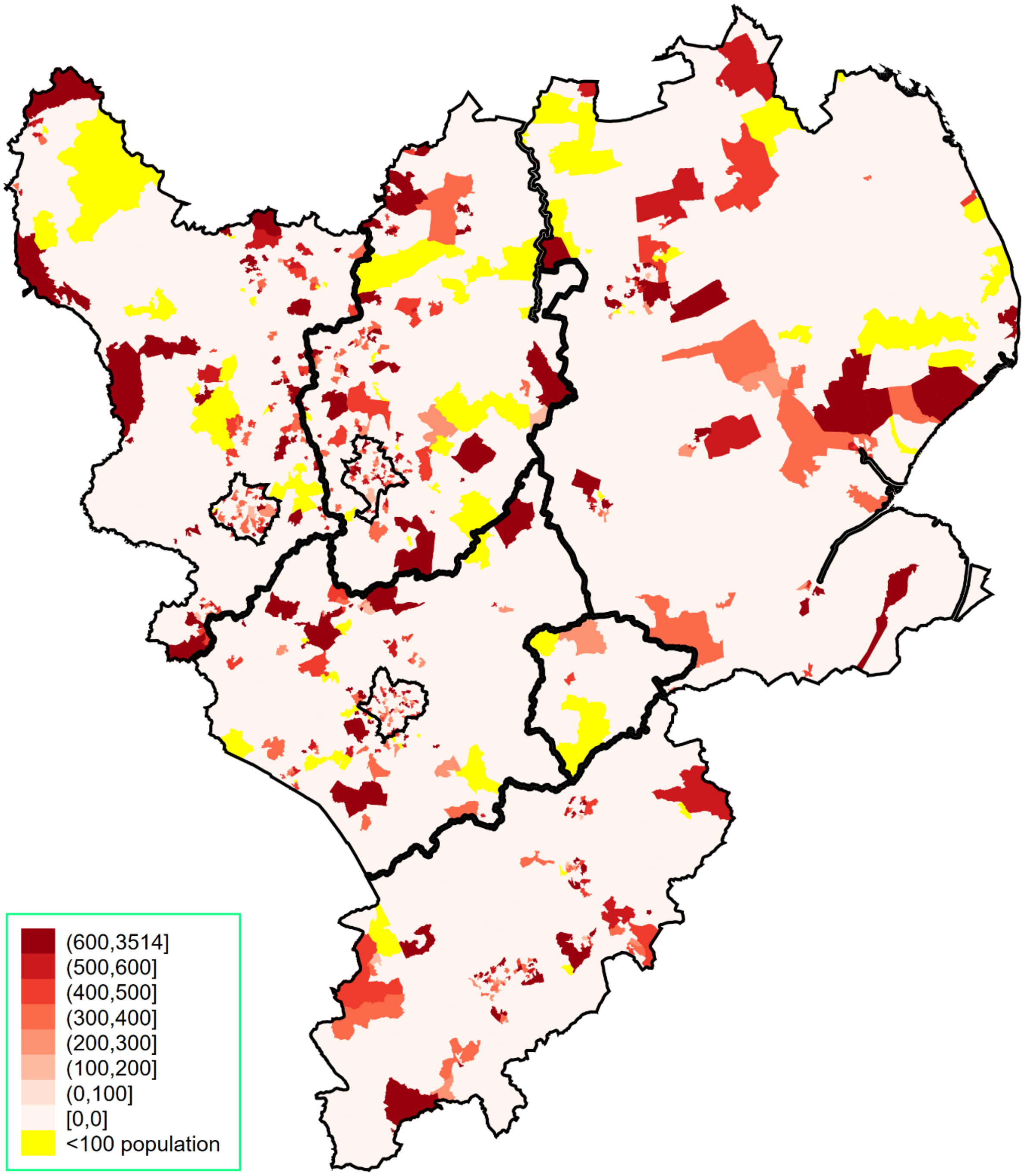


* LSOAs with fewer than 100 people aged 25-44 (i.e. “at risk”) are supressed

Figure S34: Directly age-standardised all-cause mortality rates for people aged 25 to 44 in 2016, at a low geographical level (LSOA) for East England*

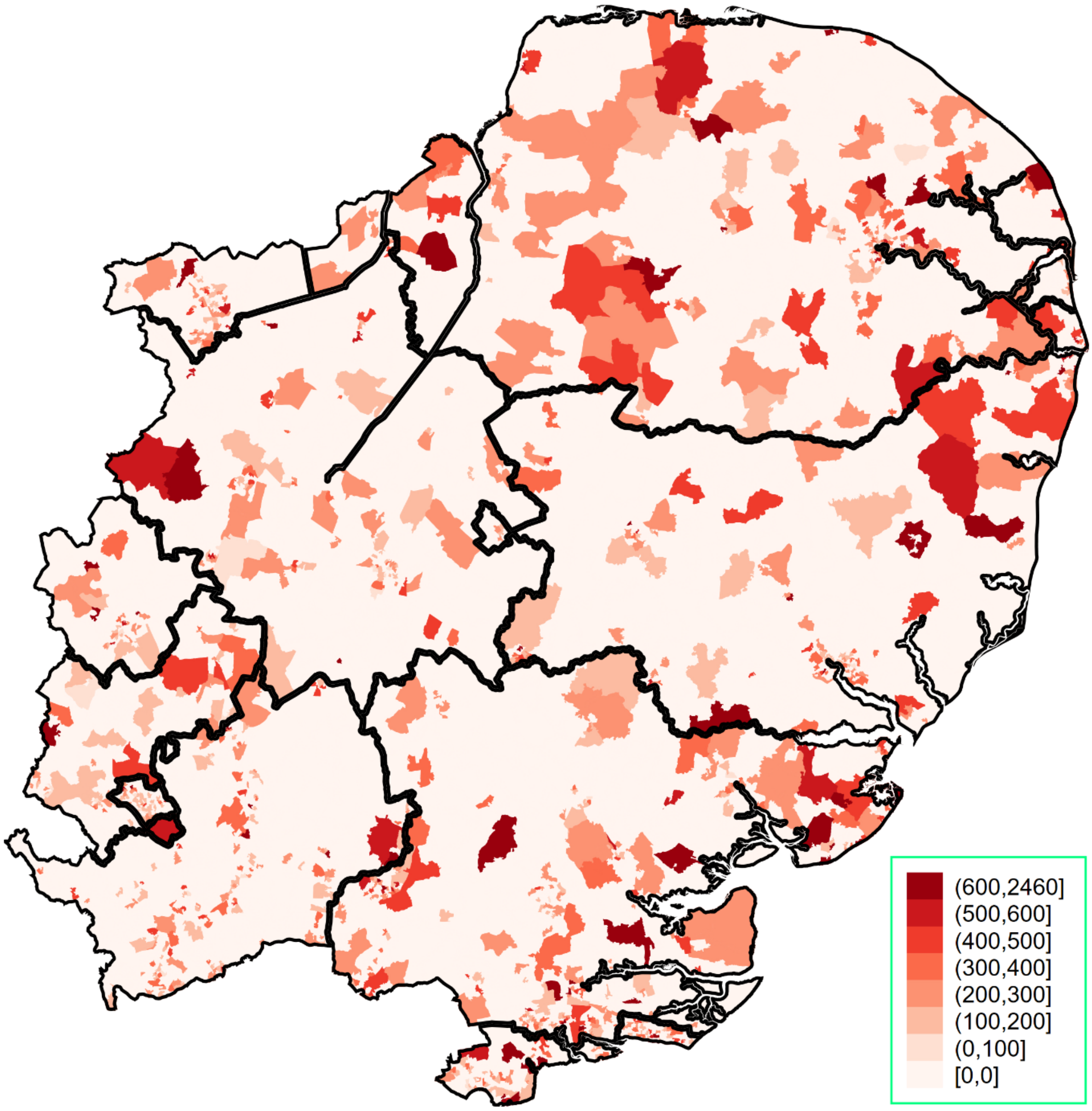


* LSOAs with fewer than 100 people aged 25-44 (i.e. "at risk") are supressed

Figure S35: Directly age-standardised all-cause mortality rates for females aged 25 to 44 in 2016, at a low geographical level (LSOA) for East England*

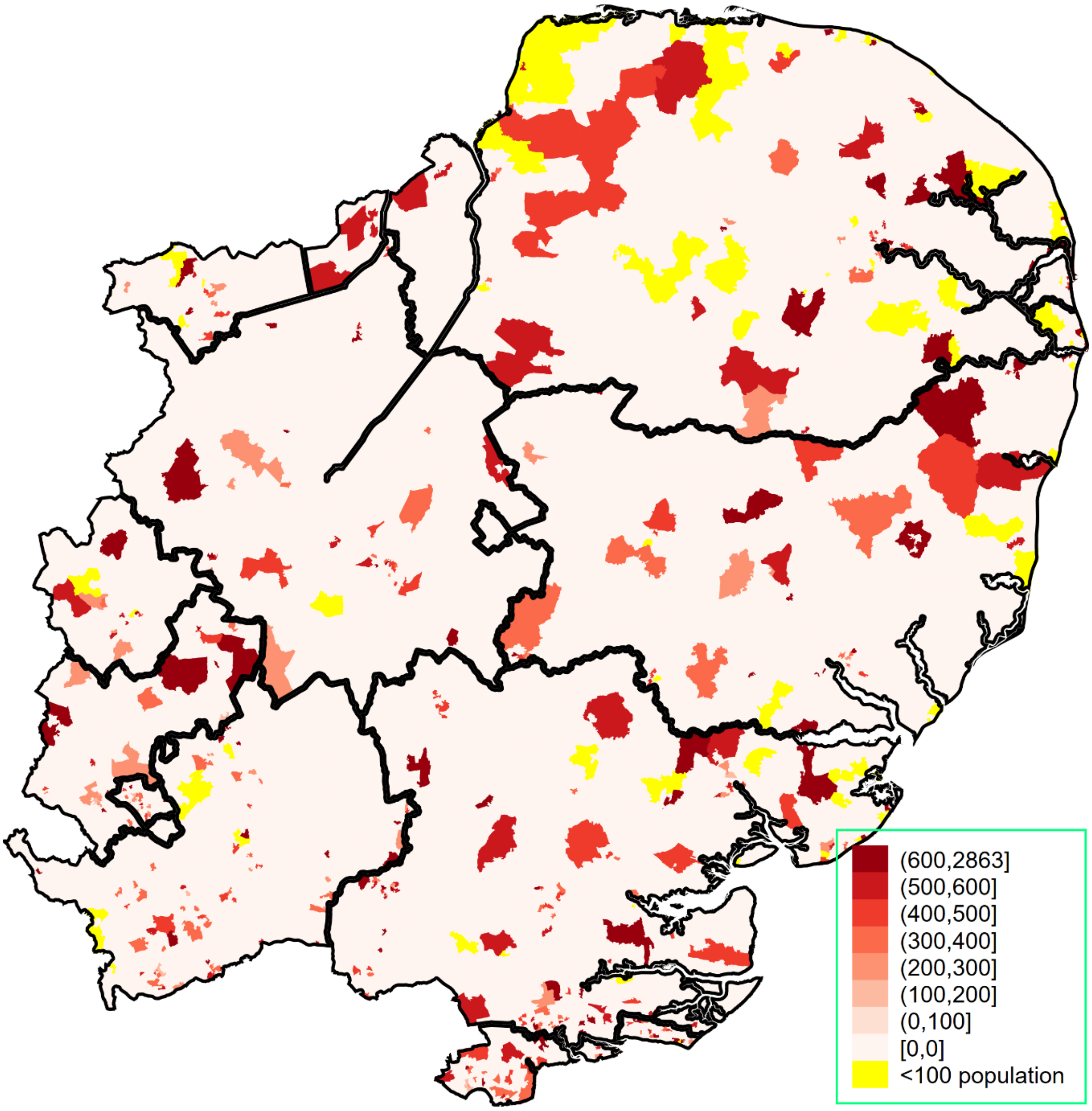


* LSOAs with fewer than 100 people aged 25-44 (i.e. "at risk") are supressed

Figure S36: Directly age-standardised all-cause mortality rates for males aged 25 to 44 in 2016, at a low geographical level (LSOA) for East England*

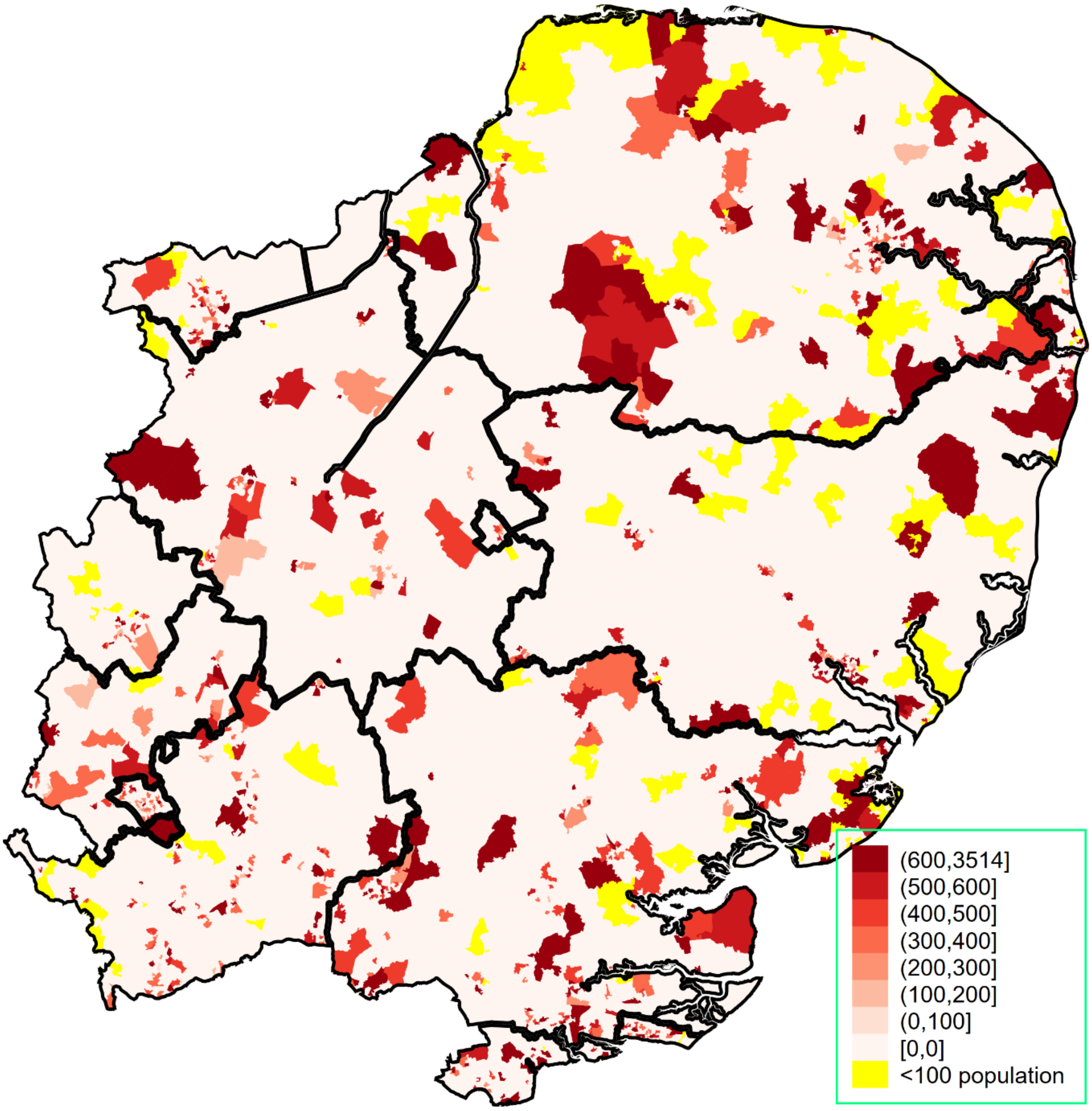


* LSOAs with fewer than 100 people aged 25-44 (i.e. "at risk") are supressed

Figure S37: Directly age-standardised all-cause mortality rates for people aged 25 to 44 in 2016, at a low geographical level (LSOA) for the North East*

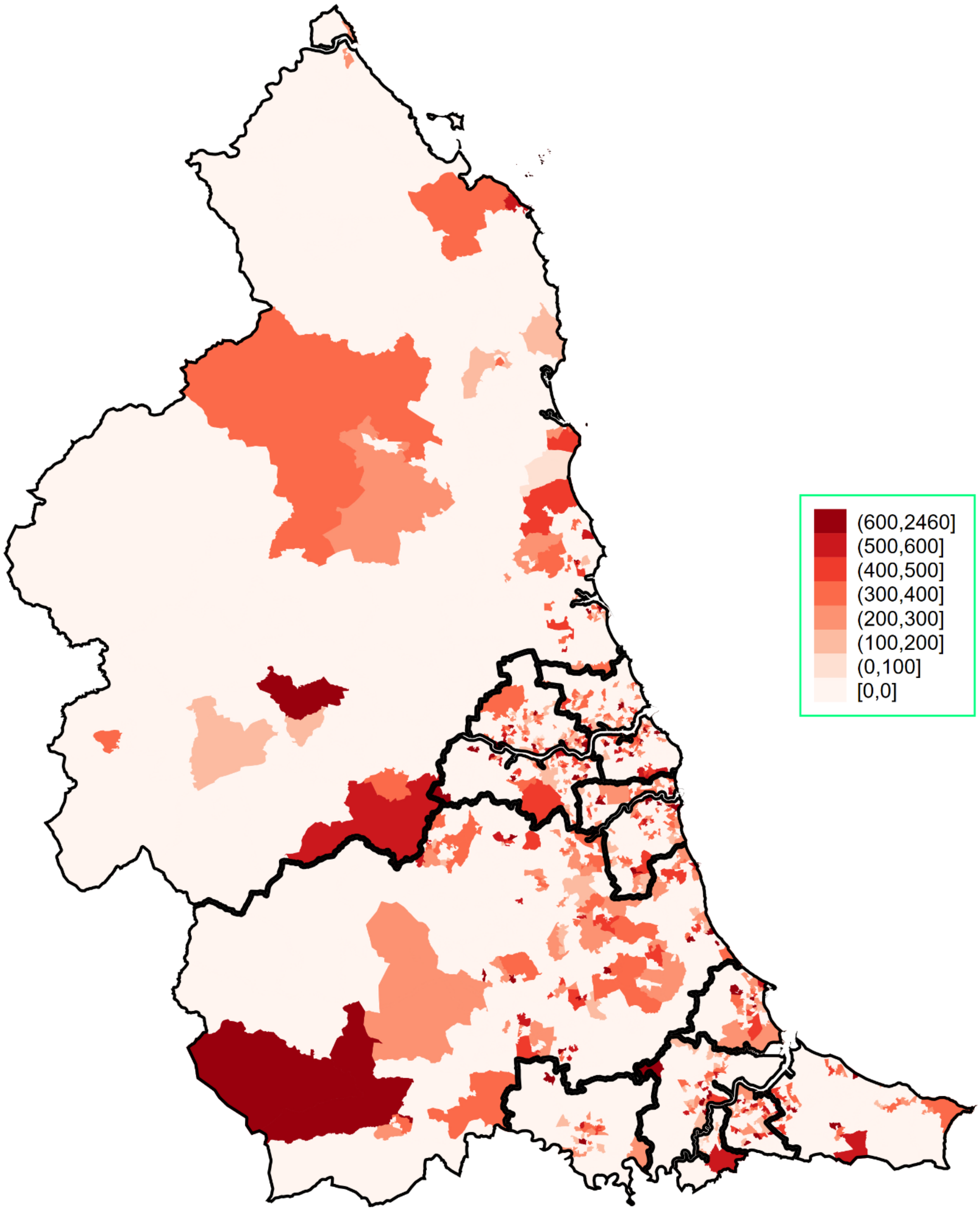


* LSOAs with fewer than 100 people aged 25-44 (i.e. "at risk") are supressed

Figure S38: Directly age-standardised all-cause mortality rates for females aged 25 to 44 in 2016, at a low geographical level (LSOA) for the North East*

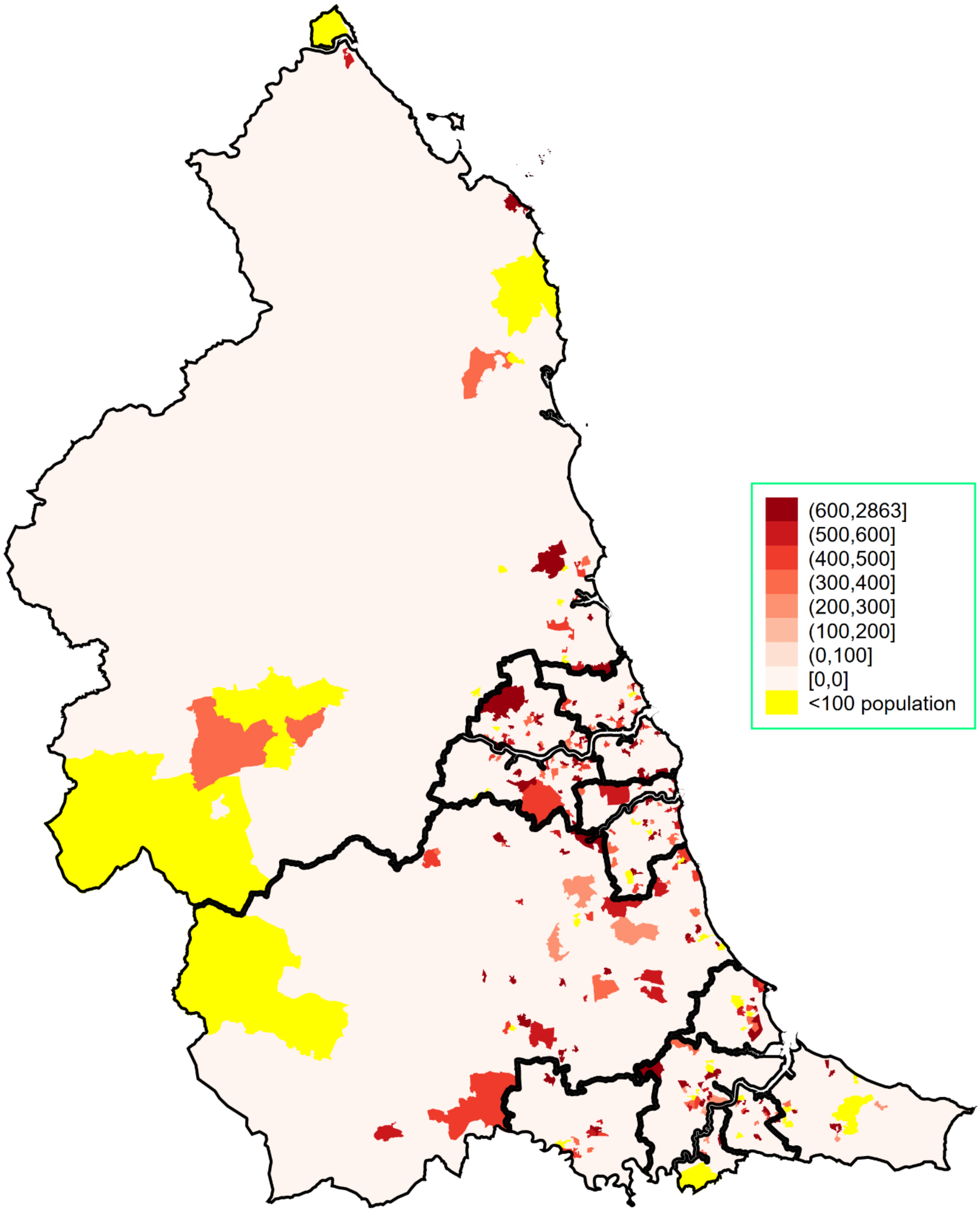


* LSOAs with fewer than 100 people aged 25-44 (i.e. “at risk”) are supressed

Figure S39: Directly age-standardised all-cause mortality rates for males aged 25 to 44 in 2016, at a low geographical level (LSOA) for the North East*

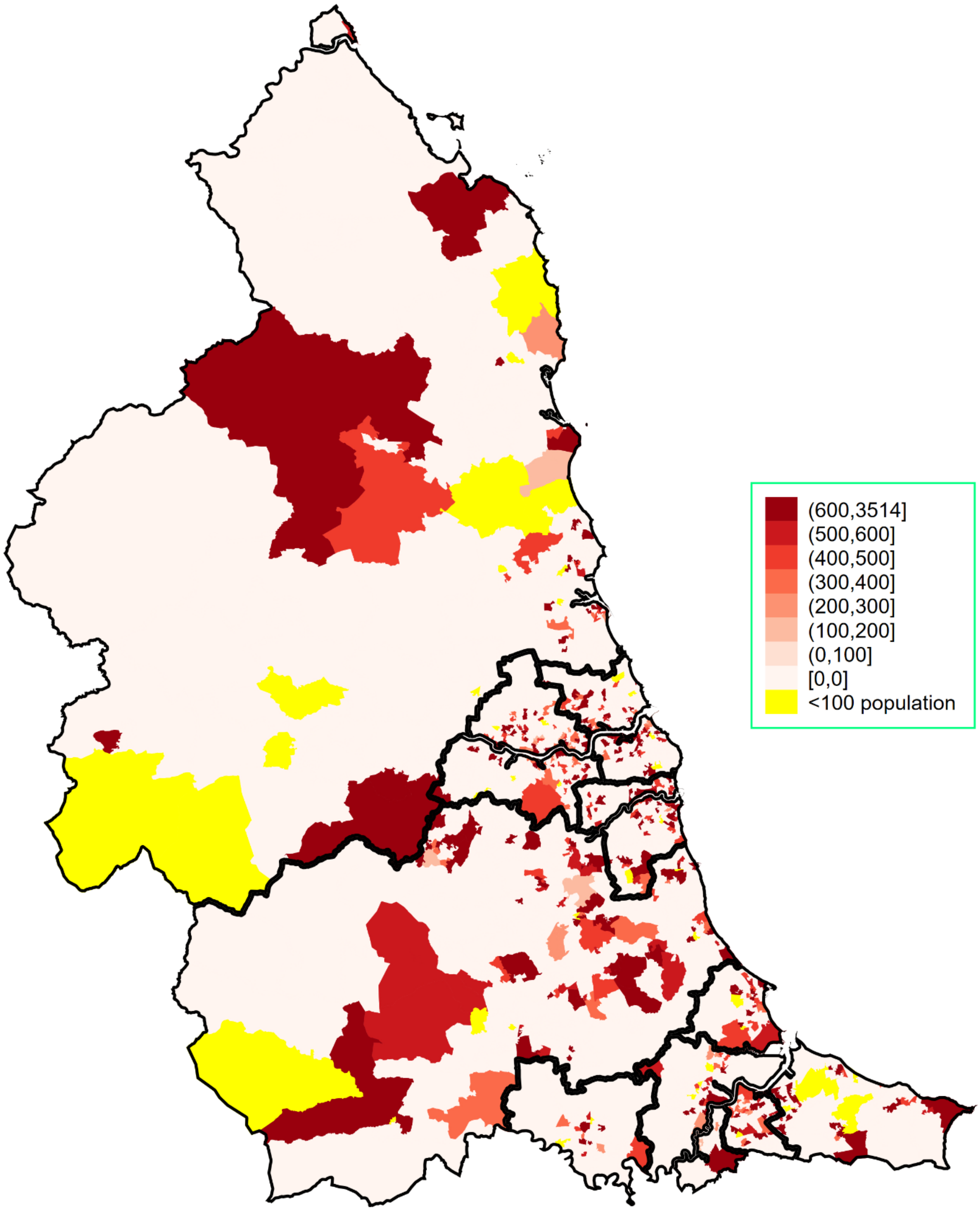


* LSOAs with fewer than 100 people aged 25-44 (i.e. “at risk”) are supressed

Figure S40: Directly age-standardised all-cause mortality rates for people aged 25 to 44 in 2016, at a low geographical level (LSOA) for the North West*

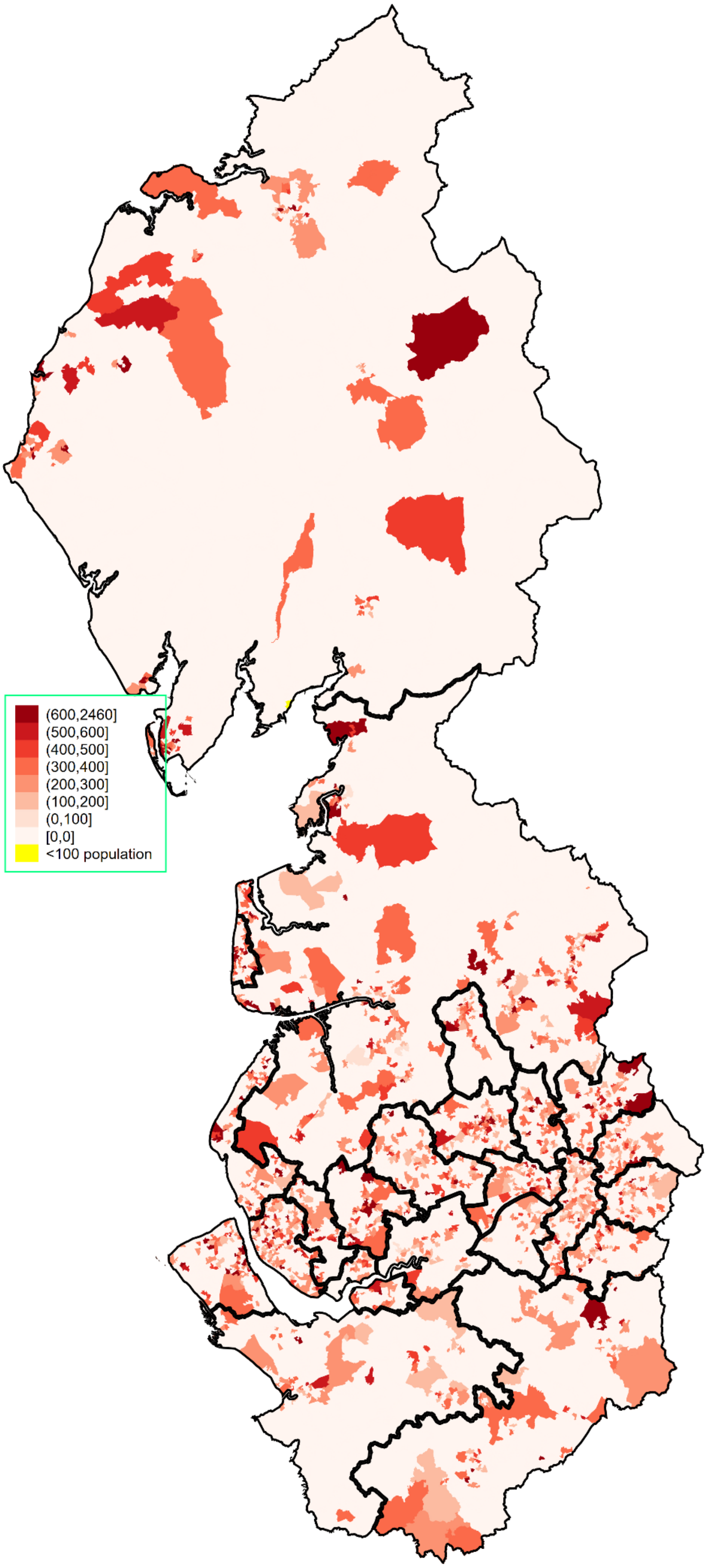


* LSOAs with fewer than 100 people aged 25-44 (i.e. "at risk") are supressed

Figure S41: Directly age-standardised all-cause mortality rates for females aged 25 to 44 in 2016, at a low geographical level (LSOA) for the North West*

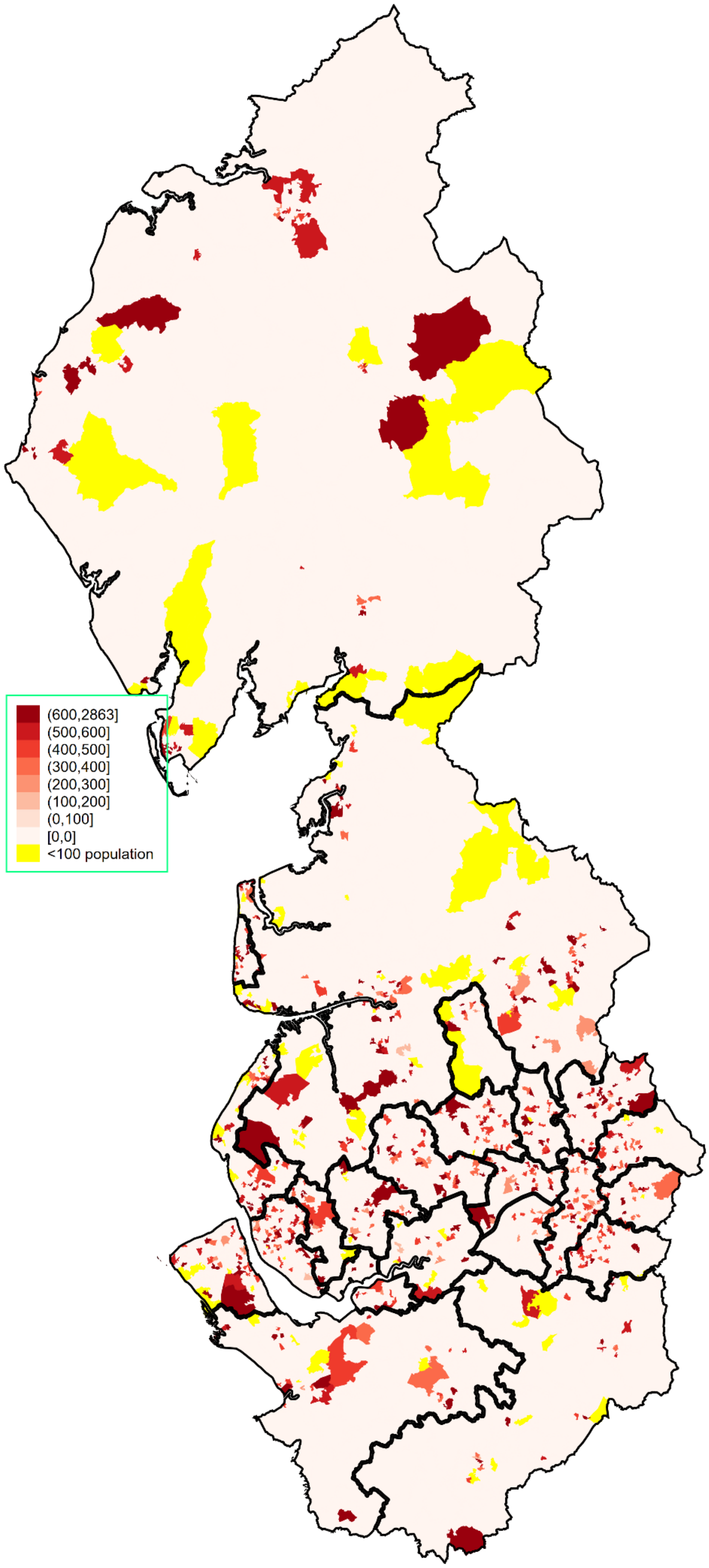


* LSOAs with fewer than 100 people aged 25-44 (i.e. "at risk") are supressed

Figure S42: Directly age-standardised all-cause mortality rates for males aged 25 to 44 in 2016, at a low geographical level (LSOA) for the North West*

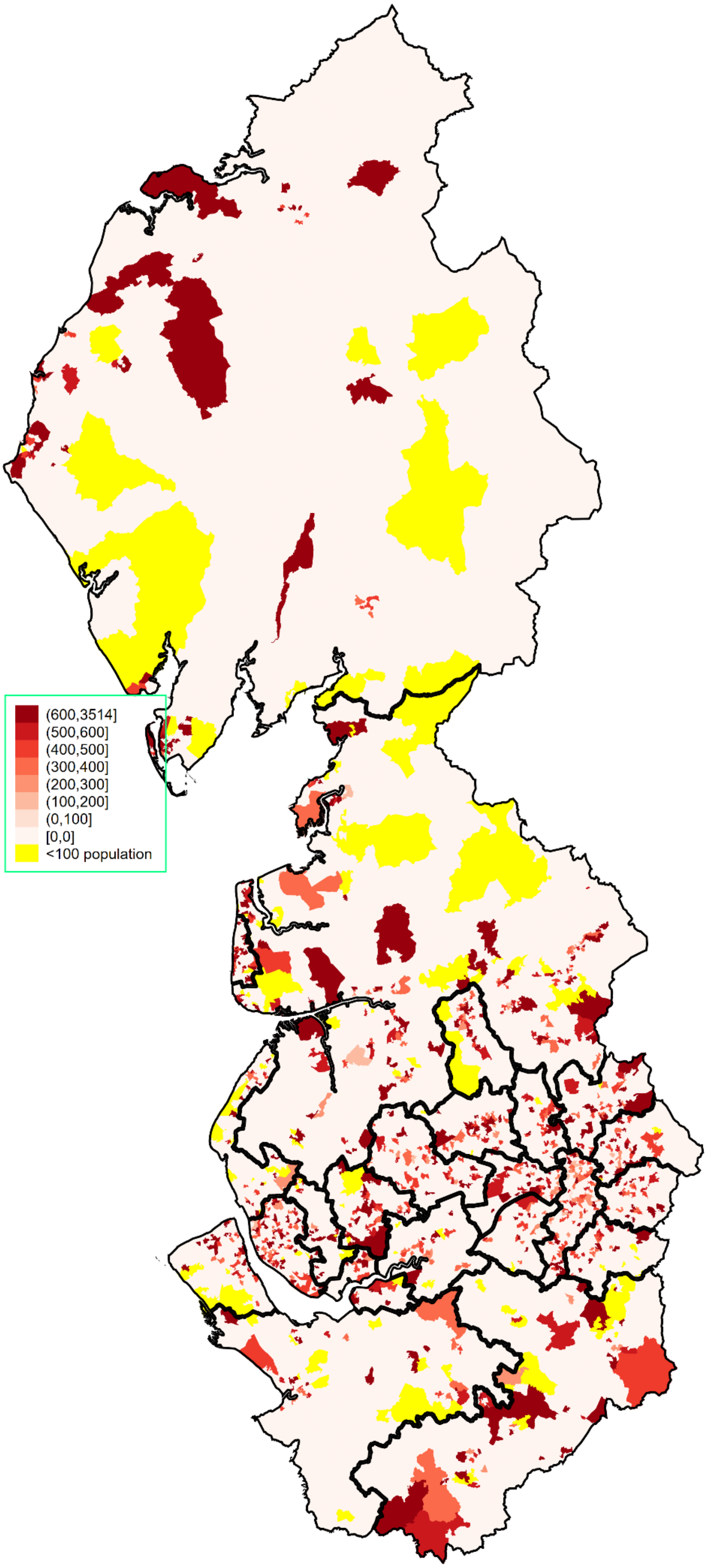


* LSOAs with fewer than 100 people aged 25-44 (i.e. "at risk") are supressed

Figure S43: Directly age-standardised all-cause mortality rates for people aged 25 to 44 in 2016, at a low geographical level (LSOA) for the South Central*

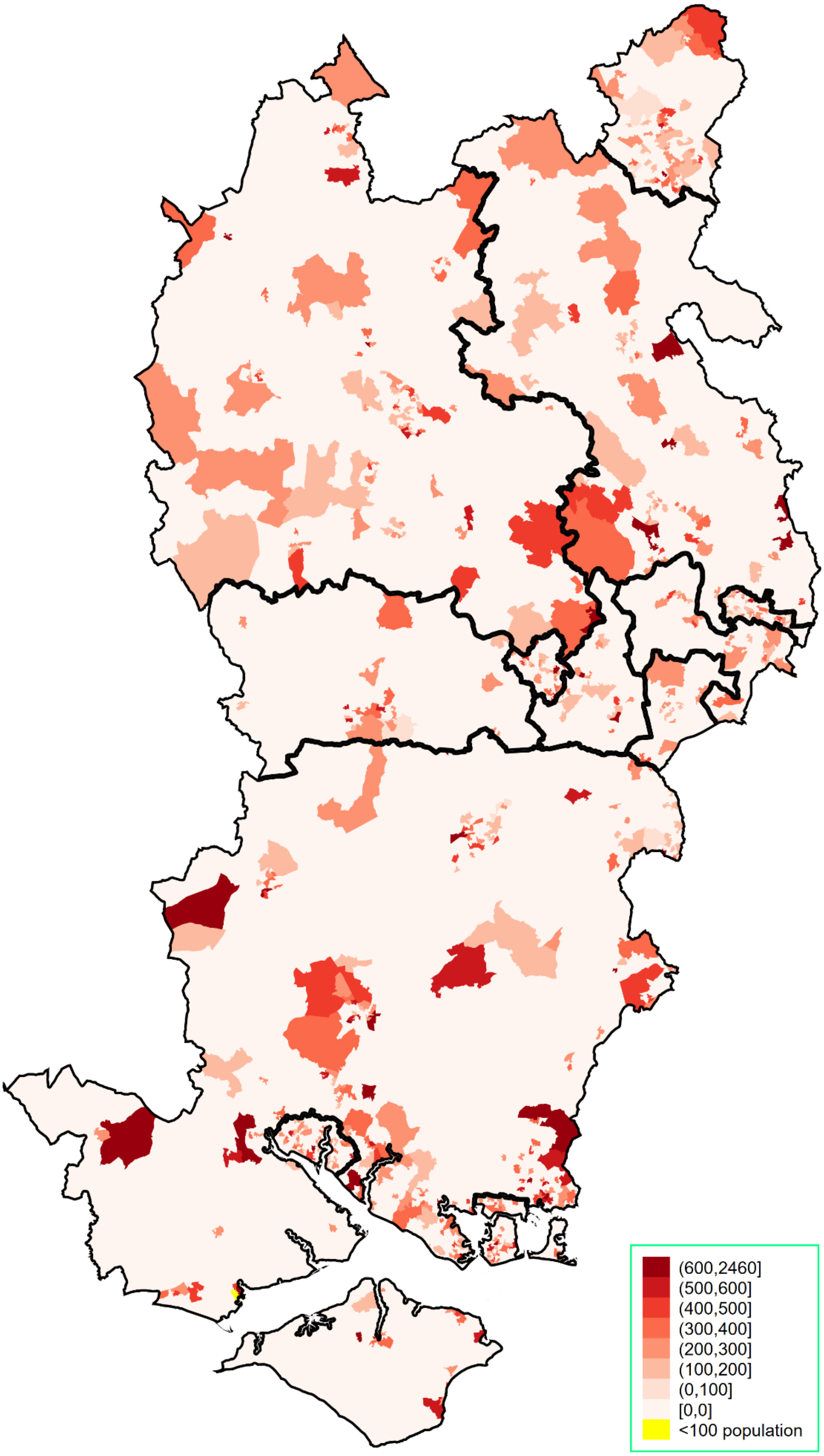


* LSOAs with fewer than 100 people aged 25-44 (i.e. "at risk") are supressed

Figure S44: Directly age-standardised all-cause mortality rates for females aged 25 to 44 in 2016, at a low geographical level (LSOA) for the South Central*

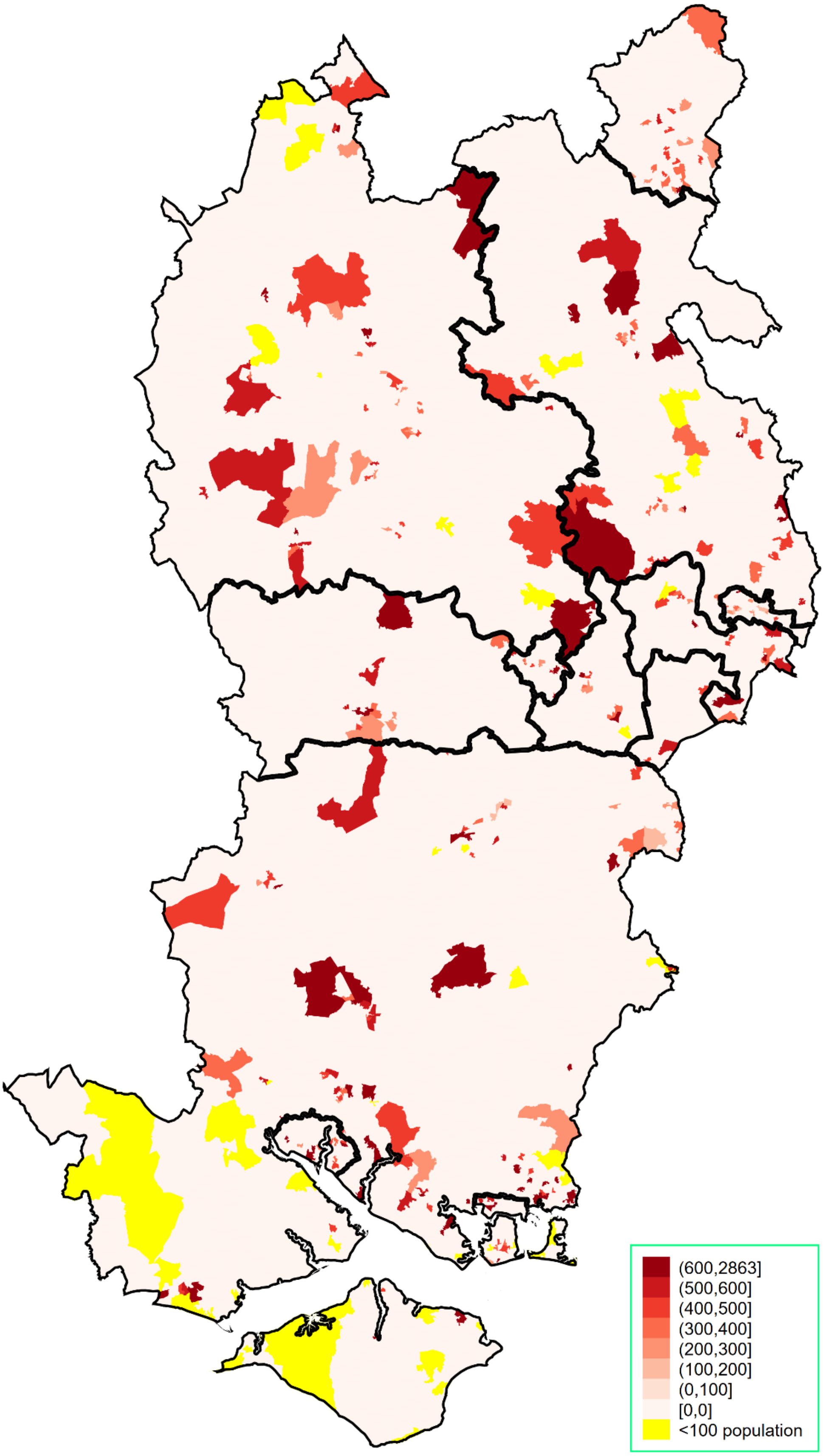


* LSOAs with fewer than 100 people aged 25-44 (i.e. “at risk”) are supressed

Figure S45: Directly age-standardised all-cause mortality rates for males aged 25 to 44 in 2016, at a low geographical level (LSOA) for the South Central*

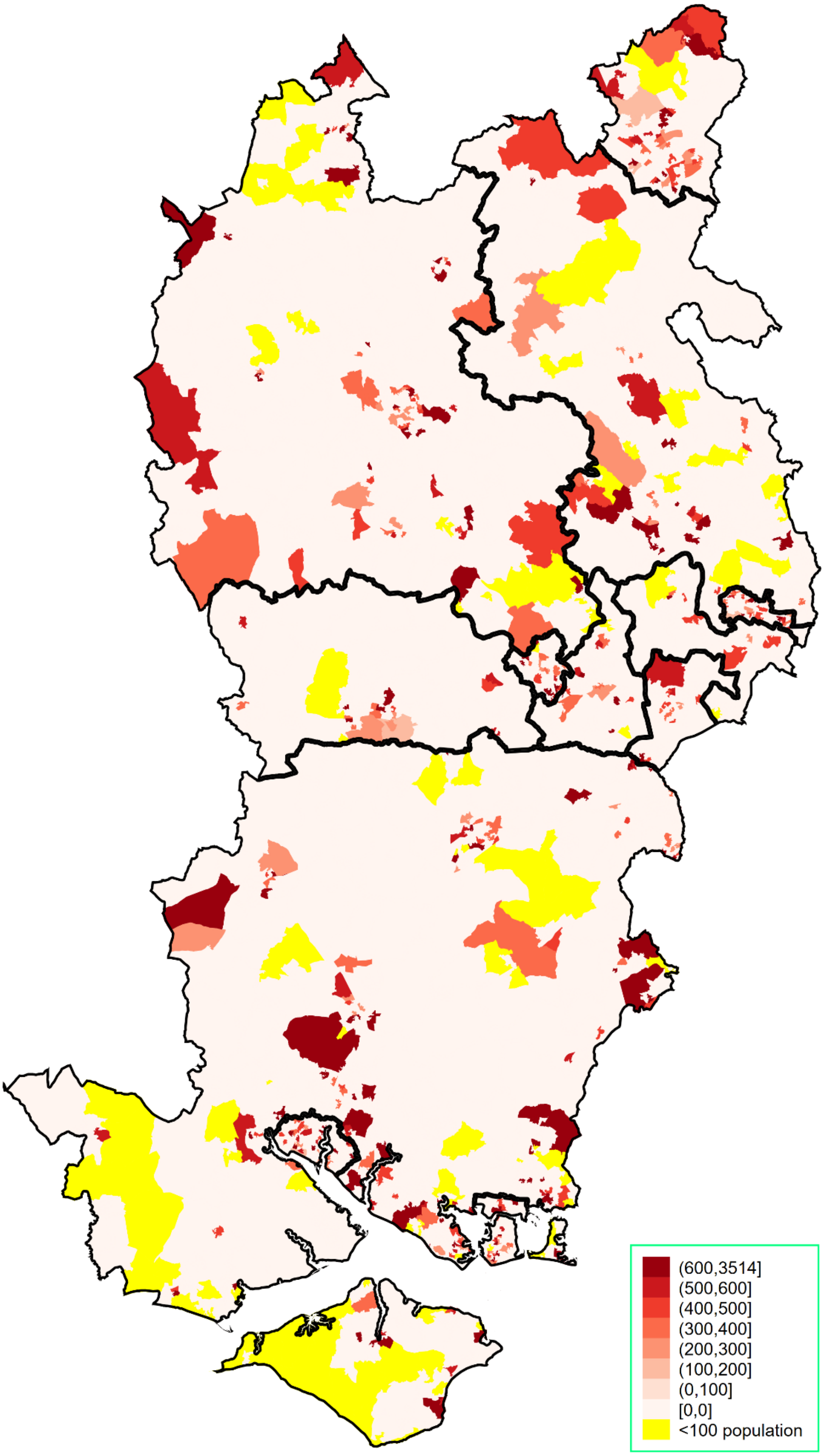


* LSOAs with fewer than 100 people aged 25-44 (i.e. "at risk") are supressed

Figure S46: Directly age-standardised all-cause mortality rates for people aged 25 to 44 in 2016, at a low geographical level (LSOA) for the South East Coast*

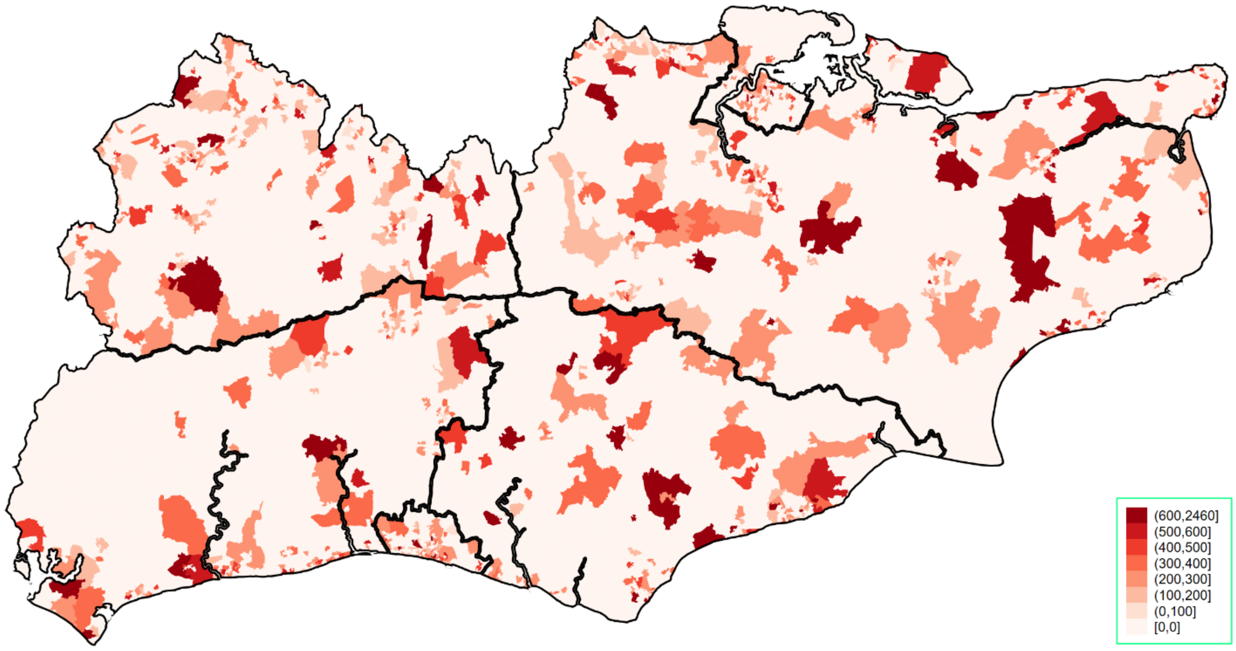


* LSOAs with fewer than 100 people aged 25-44 (i.e. "at risk") are supressed

Figure S47: Directly age-standardised all-cause mortality rates for females aged 25 to 44 in 2016, at a low geographical level (LSOA) for the South East Coast*

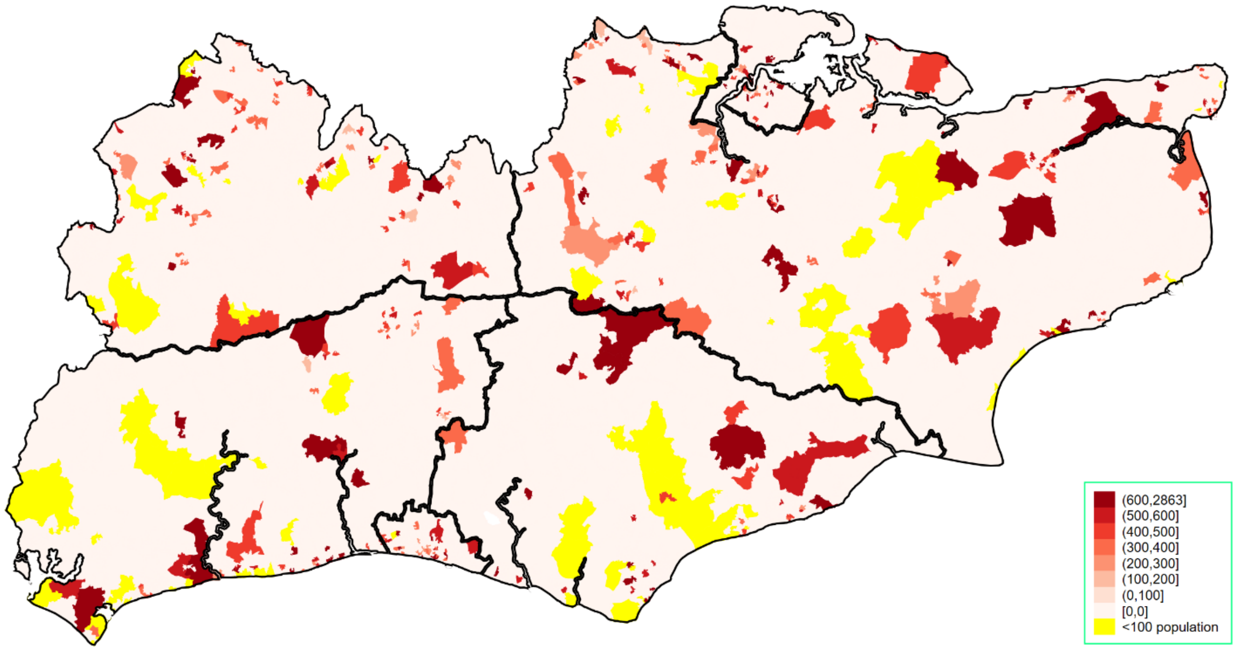


* LSOAs with fewer than 100 people aged 25-44 (i.e. "at risk") are supressed

Figure S48: Directly age-standardised all-cause mortality rates for males aged 25 to 44 in 2016, at a low geographical level (LSOA) for the South East Coast*

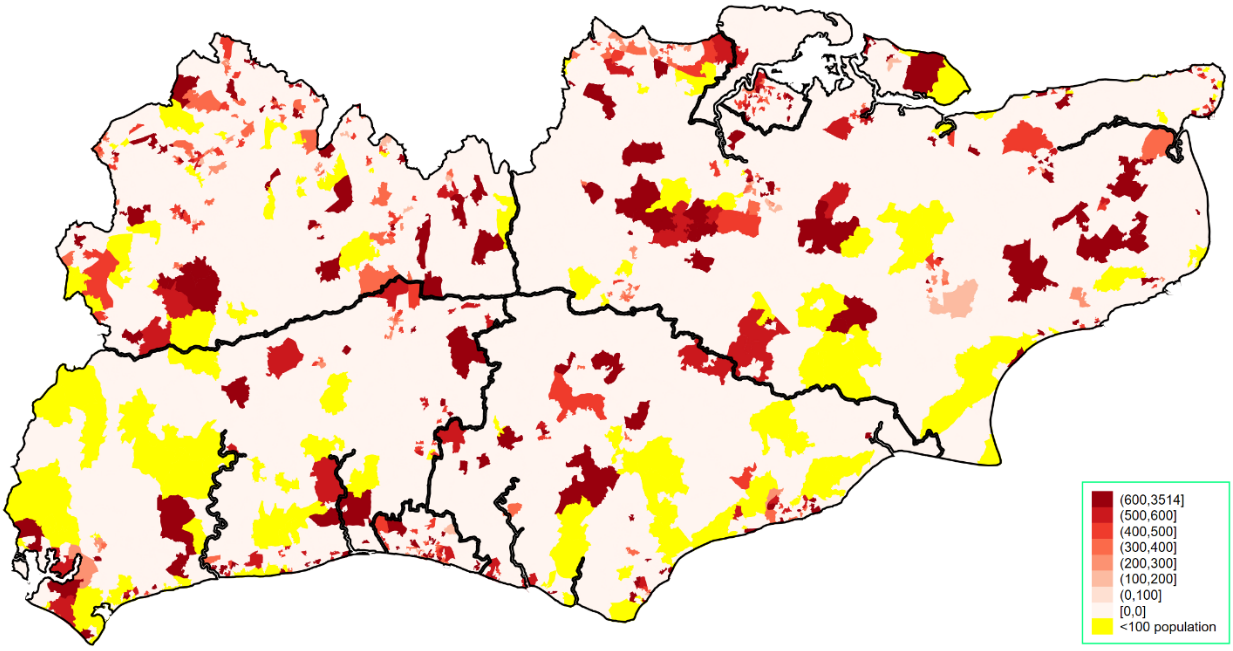


* LSOAs with fewer than 100 people aged 25-44 (i.e. “at risk”) are supressed

Figure S49: Directly age-standardised all-cause mortality rates for people aged 25 to 44 in 2016, at a low geographical level (LSOA) for the South West*

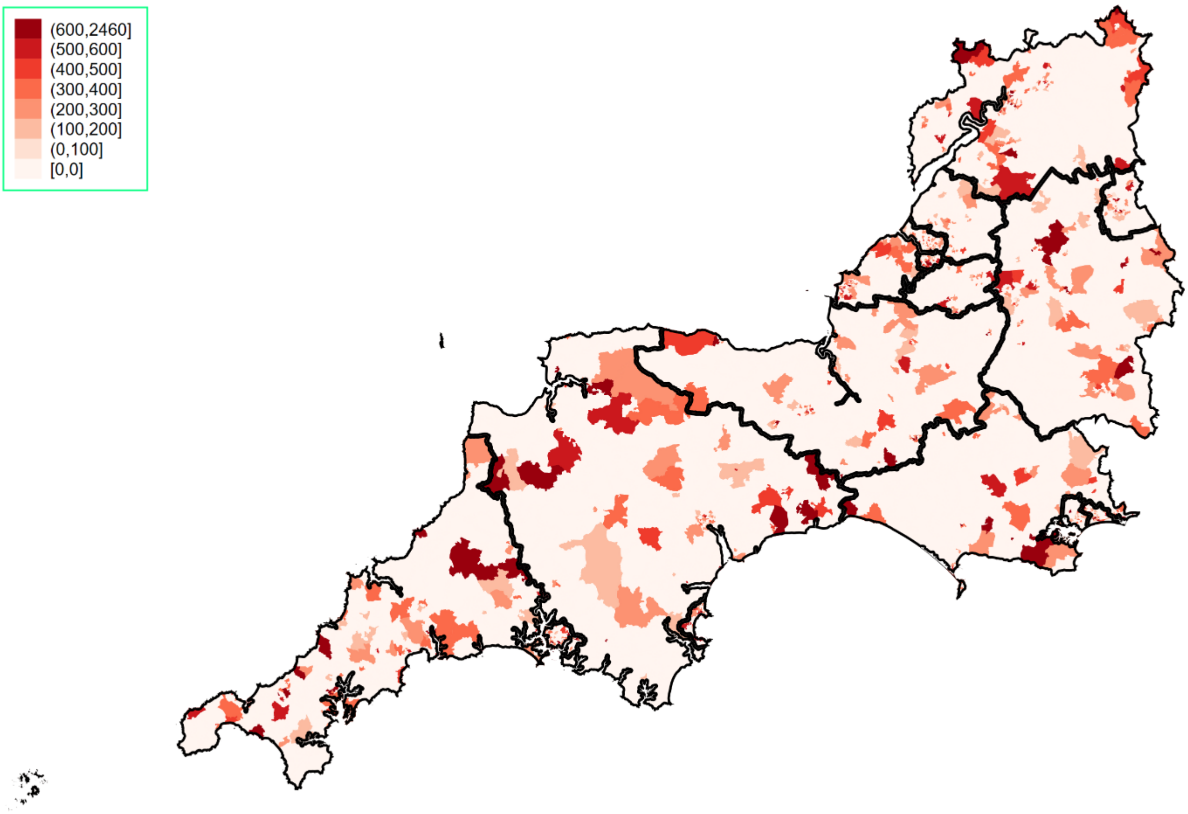


* LSOAs with fewer than 100 people aged 25-44 (i.e. "at risk") are supressed

Figure S50: Directly age-standardised all-cause mortality rates for females aged 25 to 44 in 2016, at a low geographical level (LSOA) for the South West*

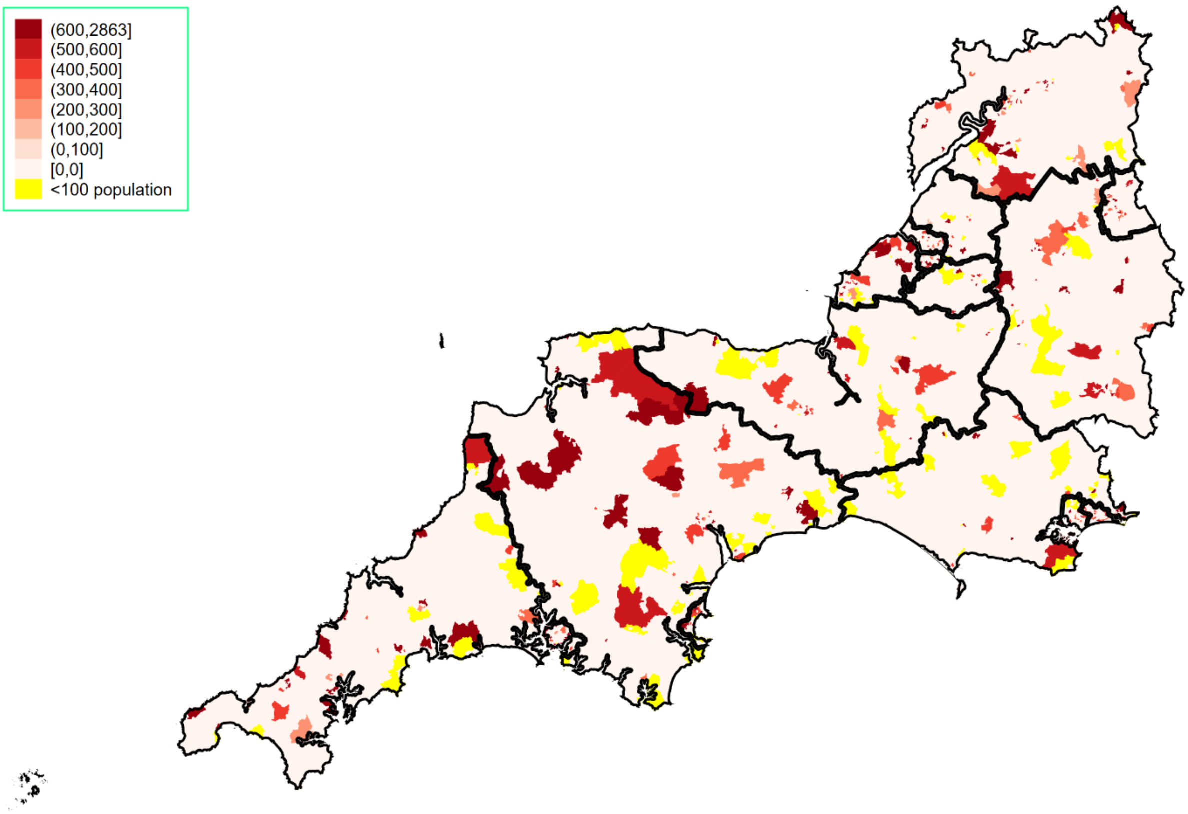


* LSOAs with fewer than 100 people aged 25-44 (i.e. "at risk") are supressed

Figure S51: Directly age-standardised all-cause mortality rates for males aged 25 to 44 in 2016, at a low geographical level (LSOA) for the South West*

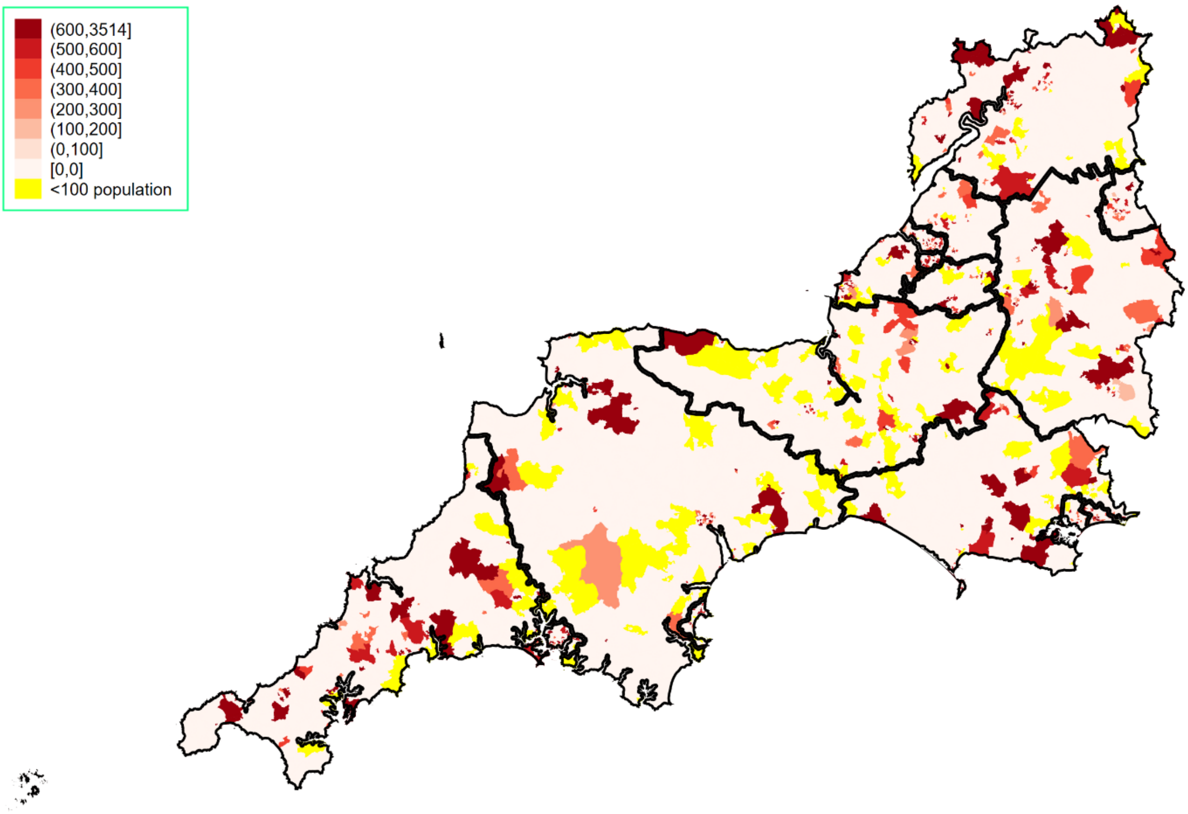


* LSOAs with fewer than 100 people aged 25-44 (i.e. “at risk”) are supressed

Figure S52: Directly age-standardised all-cause mortality rates for people aged 25 to 44 in 2016, at a low geographical level (LSOA) for the West Midlands*

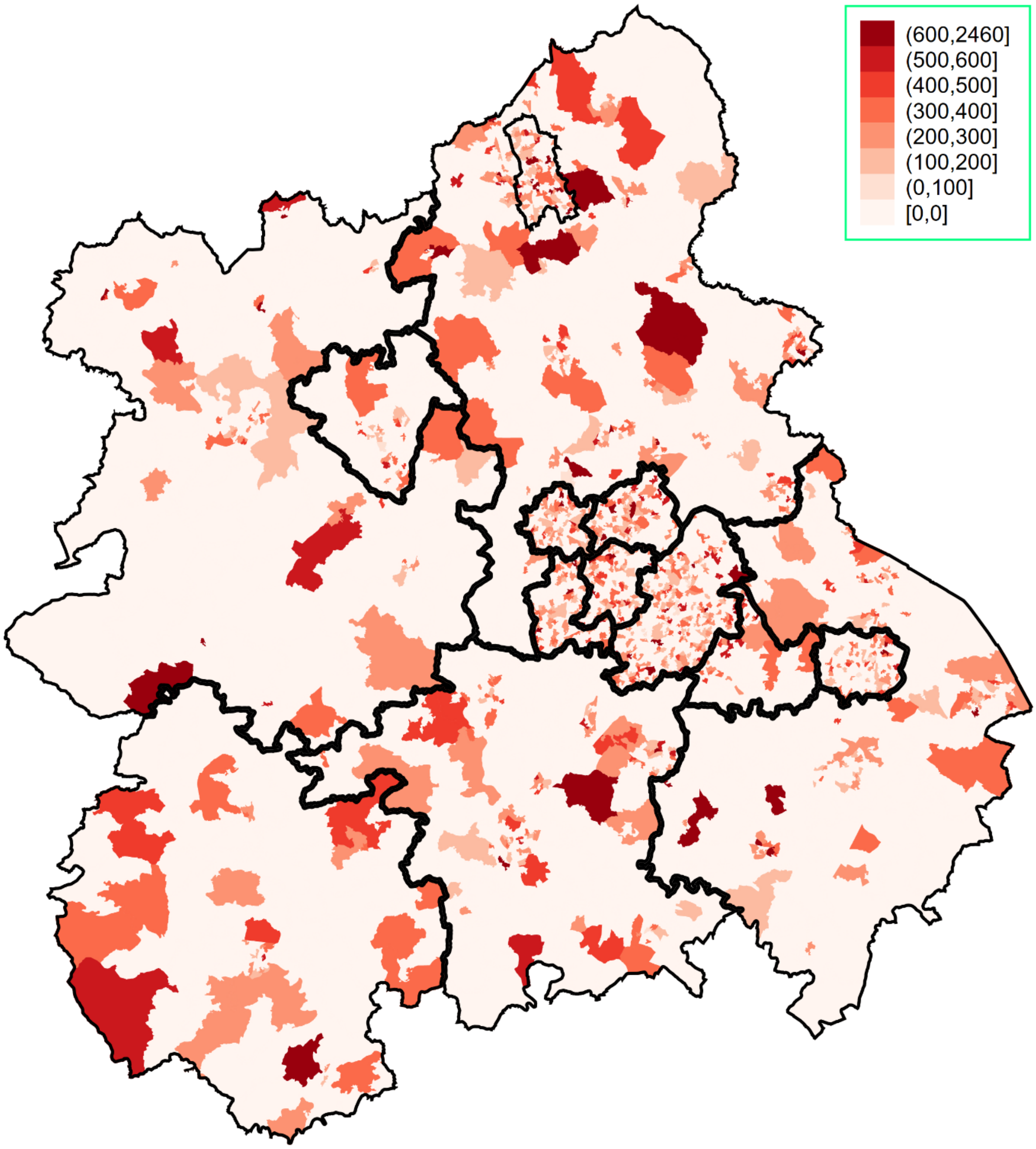


* LSOAs with fewer than 100 people aged 25-44 (i.e. “at risk”) are supressed

Figure S53: Directly age-standardised all-cause mortality rates for females aged 25 to 44 in 2016, at a low geographical level (LSOA) for the West Midlands*

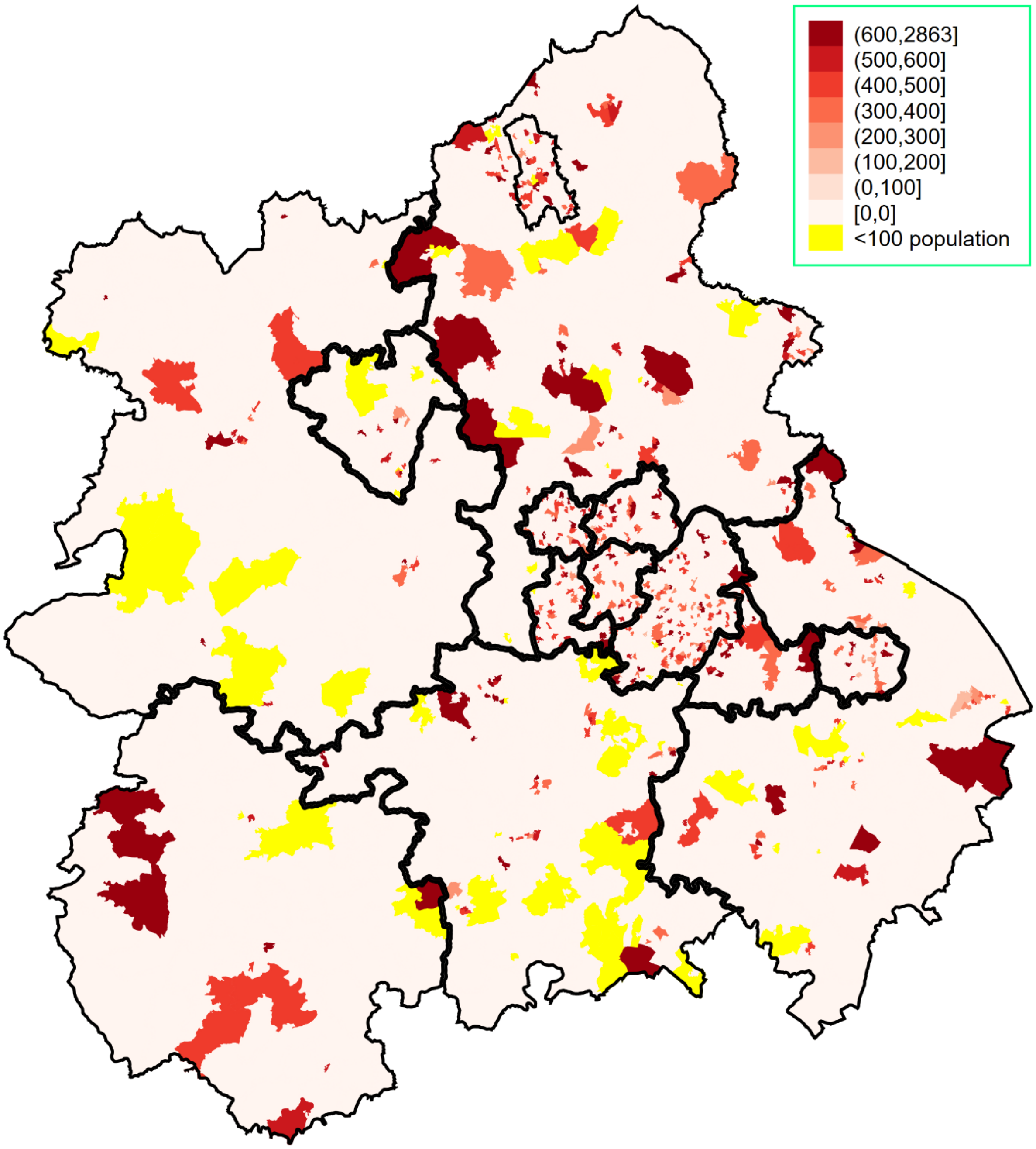


* LSOAs with fewer than 100 people aged 25-44 (i.e. “at risk”) are supressed

Figure S54: Directly age-standardised all-cause mortality rates for males aged 25 to 44 in 2016, at a low geographical level (LSOA) for the West Midlands*

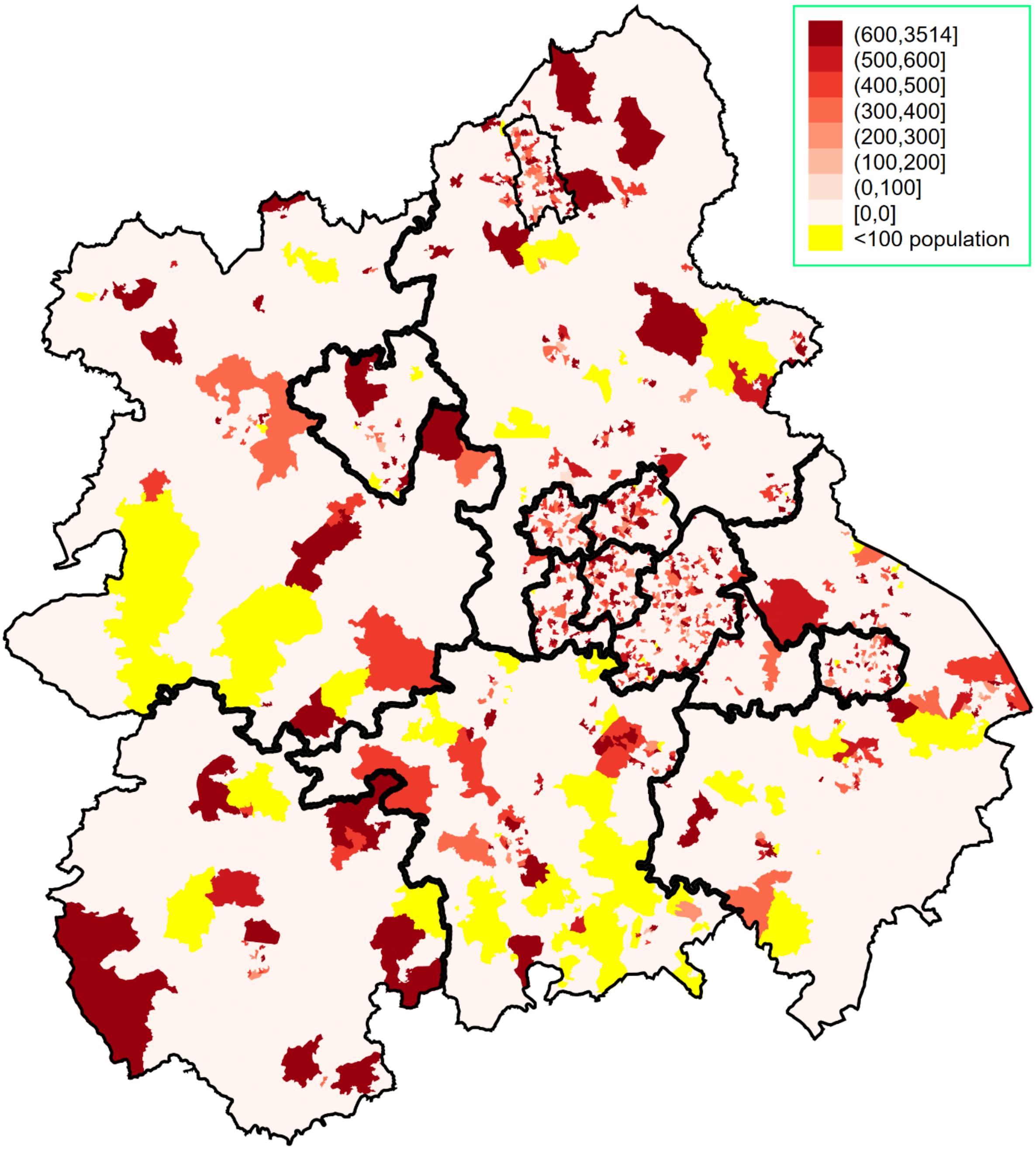


* LSOAs with fewer than 100 people aged 25-44 (i.e. "at risk") are supressed

Figure S55: Directly age-standardised all-cause mortality rates for people aged 25 to 44 in 2016, at a low geographical level (LSOA) for Yorkshire and Humber*

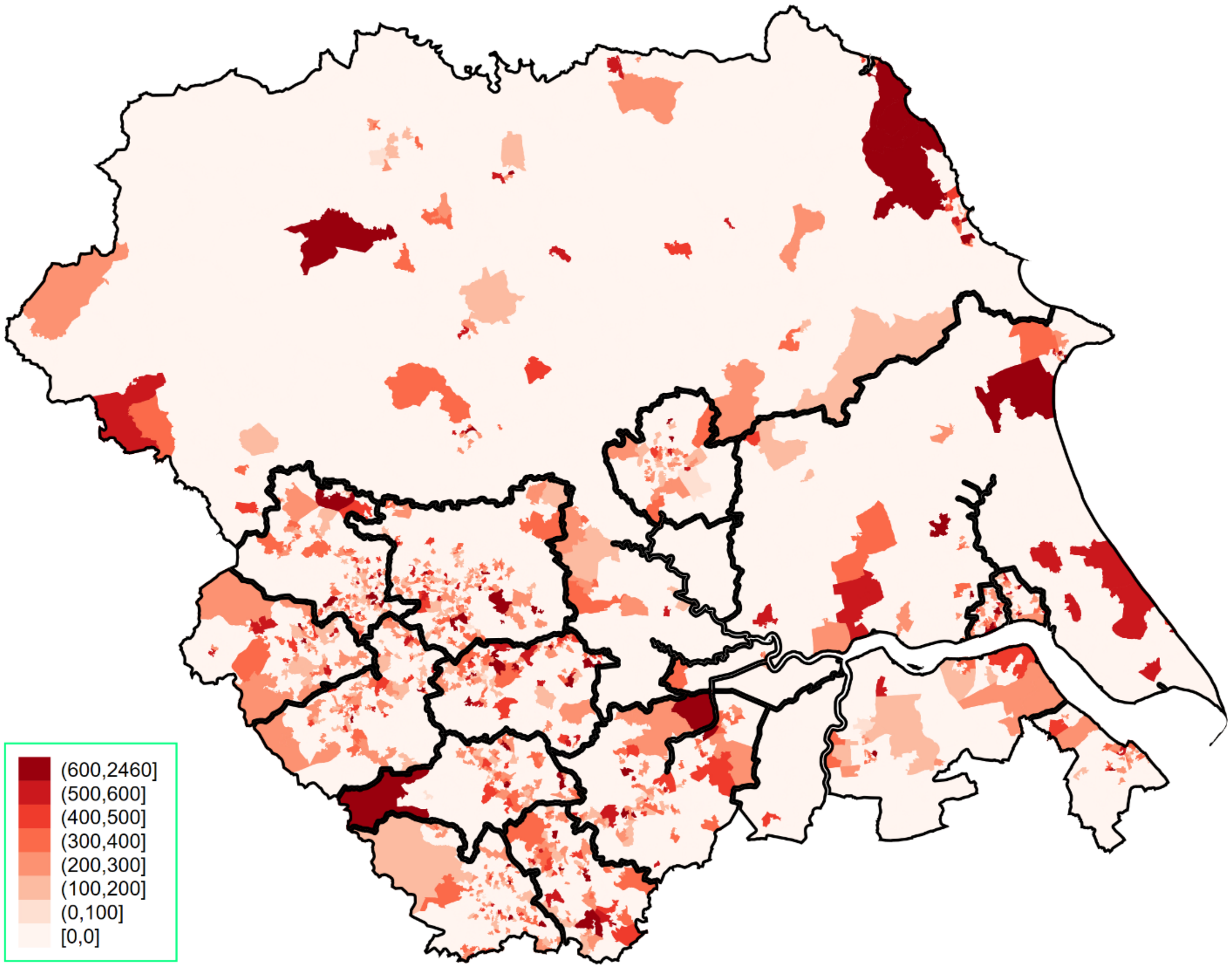


* LSOAs with fewer than 100 people aged 25-44 (i.e. "at risk") are supressed

Figure S56: Directly age-standardised all-cause mortality rates for females aged 25 to 44 in 2016, at a low geographical level (LSOA) for the Yorkshire and Humber*

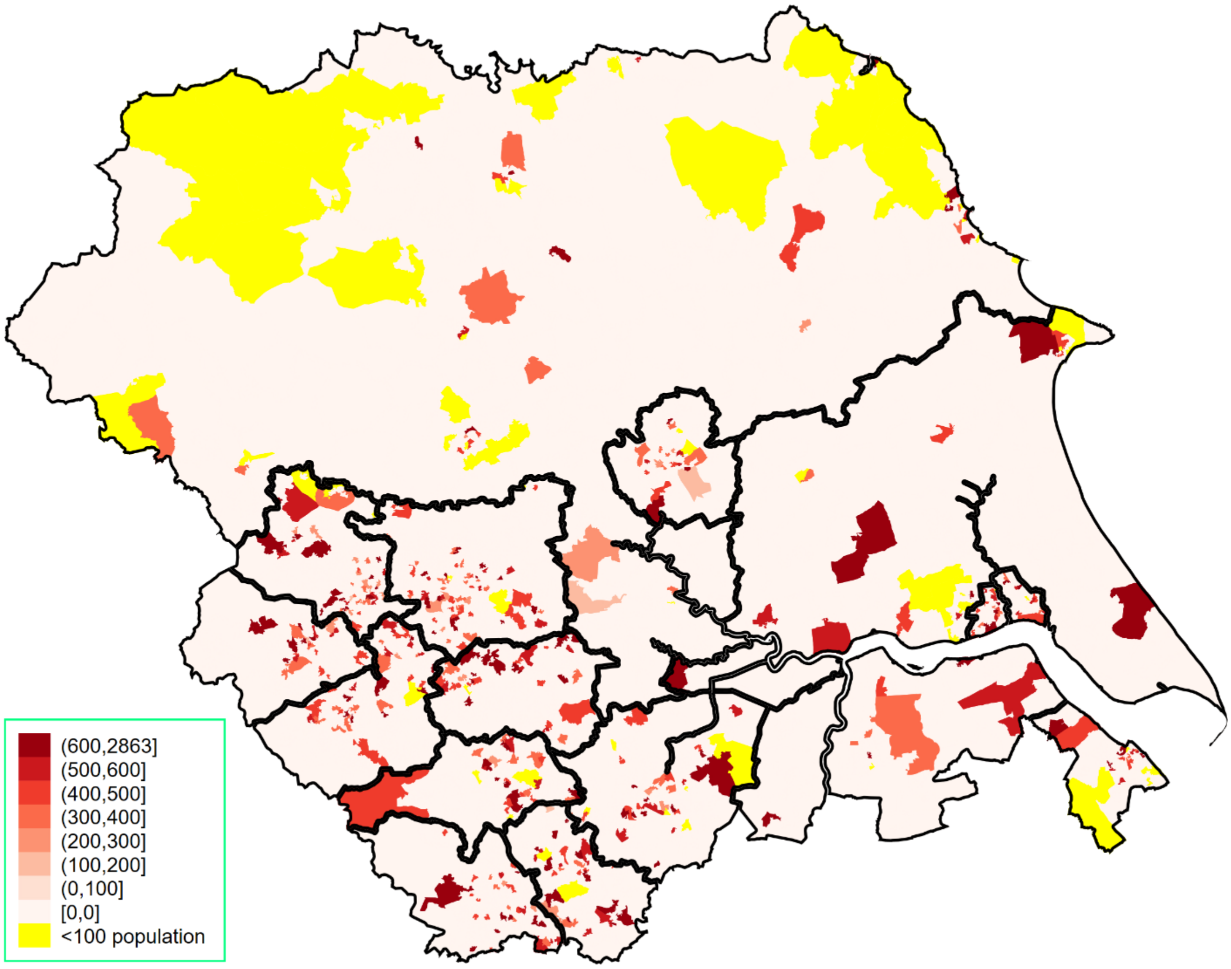


* LSOAs with fewer than 100 people aged 25-44 (i.e. "at risk") are supressed

Figure S57: Directly age-standardised all-cause mortality rates for males aged 25 to 44 in 2016, at a low geographical level (LSOA) for the Yorkshire and Humber*

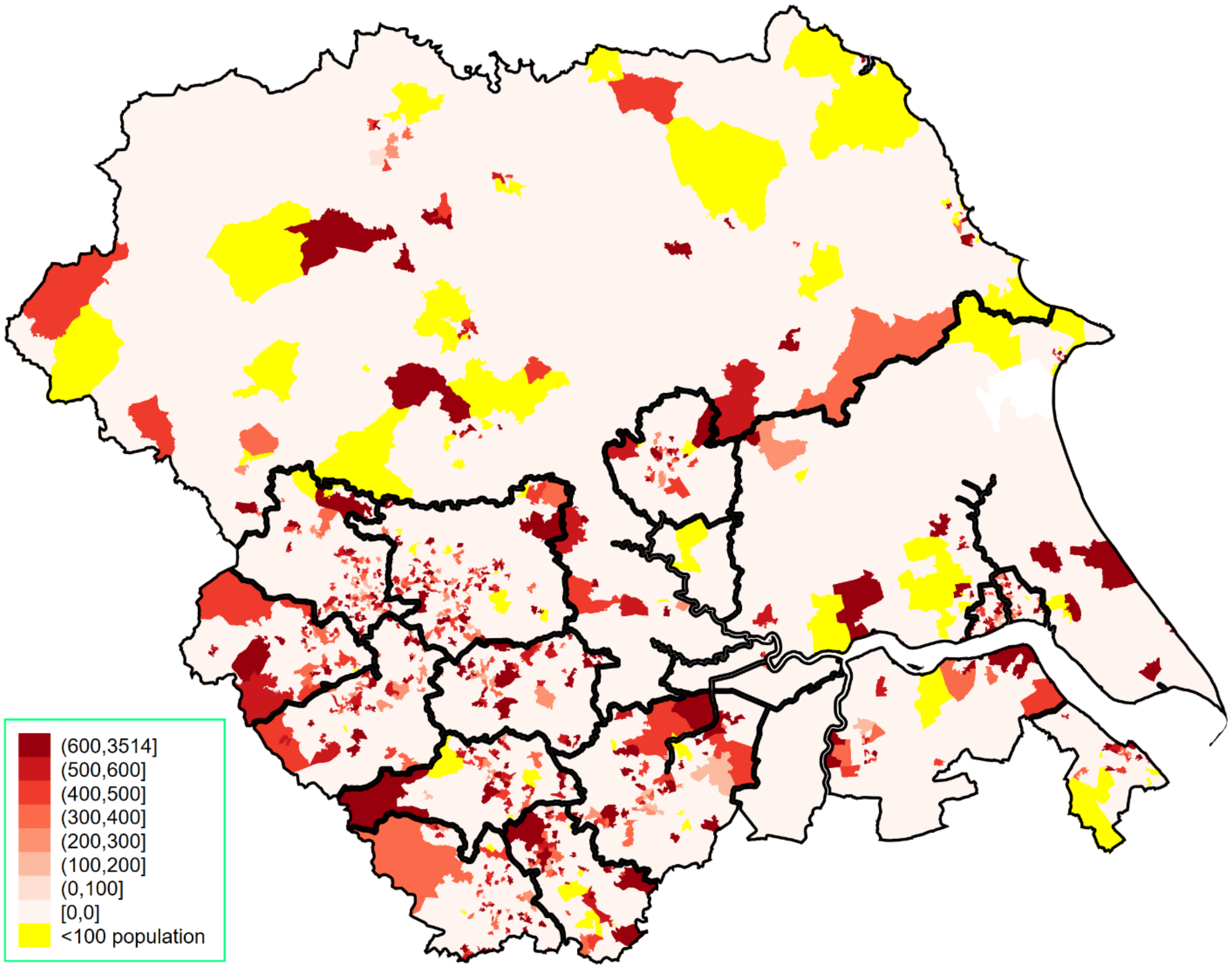


* LSOAs with fewer than 100 people aged 25-44 (i.e. "at risk") are supressed

## Get data

```
local dir1 "P:/Evan/Informatics/North-South divide/specific-causes/"

local dir2 "`dir1'/original_data/"

local dir3 "P:/Evan/Informatics/North-South divide/all-causes/"

cd "`dir1'"

tempfile tempe tempf

set more off


//get denominators

use "`dir3'dataset", clear

qui keep if year>=1981

save `tempe', replace


//get cause-specific from 1981

local cntr=0

foreach cau in "Accidents" "Alcohol" "Cardiovascular" "Diabetes" "Obesity" "Suicide"
"Cancer (exc breast)" "Breast cancer" {

*local cau "Accidents"

        local cntr=`cntr'+1

        import excel "`dir2'deathsnorthsouthdivideengland1981to2016.xlsx", sheet("`cau'")
firstrow cellrange(A4:BW43) clear

        drop AM

        drop in 20

        replace Area="North" in 1/19

        replace Area="South" in 20/38

        local yr=1980
```

```
foreach x of varlist C-AL {
	local yr=`yr'+1
	rename `x' males`yr'
}
local yr=1980
foreach x of varlist AN-BW {
	local yr=`yr'+1
	rename `x' females`yr'
}
qui encode Area, gen(north)
label drop north
qui replace north=0 if north==2
capture rename Agegroup agegroup
rename agegroup ageband
keep north ageband males* females*
reshape long males females, i(ageband north) j(year)
rename males deaths1
rename females deaths0
reshape long deaths, i(ageband north year) j(male)
replace ageband=trim(ageband)
label define agelbl 0 "<1"
encode ageband, gen(agecat) label(agelbl)
label define agelbl 0 "<01", modify
qui replace ageband="<01" if inlist(ageband,"01","<1")
rename deaths deaths`cntr'
label var deaths`cntr' "Num of deaths: `cau'"
```

```
        //save
        capture save `tempf'
        if _rc!=0 {
                merge 1:1 agecat male year north using `tempf', nogen
                save `tempf', replace
        }
}

//get cause-specific from 1993
foreach cau in "Drug" {
*local cau "Accidents"
        local cntr=`cntr'+1
        import excel "`dir2'deathsnorthsouthdivideengland1981to2016.xlsx", sheet("`cau'")
firstrow cellrange(A4:AY43) clear
        drop AA
        drop in 20
        replace Area="North" in 1/19
        replace Area="South" in 20/38
        local yr=1992
        foreach x of varlist C-Z {
                local yr=`yr'+1
                rename `x' males`yr'
        }
        local yr=1992
        foreach x of varlist AB-AY {
                local yr=`yr'+1
```

```
		rename `x' females`yr'
	}
	qui encode Area, gen(north)
	label drop north
	qui replace north=0 if north==2
	capture rename Agegroup agegroup
	rename agegroup ageband
	keep north ageband males* females*
	reshape long males females, i(ageband north) j(year)
	rename males deaths1
	rename females deaths0
	reshape long deaths, i(ageband north year) j(male)
	replace ageband=trim(ageband)
	label define agelbl 0 "<1"
	encode ageband, gen(agecat) label(agelbl)
	label define agelbl 0 "<01", modify
	qui replace ageband="<01" if inlist(ageband,"01","<1")
	rename deaths deaths`cntr'
	label var deaths`cntr' "Num of deaths: `cau'"
	//save
	merge 1:1 agecat male year north using `tempf', nogen
	save `tempf', replace
}


//add population data
```

```
merge 1:1 agecat male year north using `tempe', nogen
sort north male agecat
rename deaths deaths0

//aggregate cario/diabetes/obesity
qui replace deaths3=deaths3+deaths4+deaths5
label var deaths3 "Num of deaths: Cardiovascular, diabetes and obesity"
qui drop deaths4
qui drop deaths5
rename deaths6 deaths4
rename deaths9 deaths5
rename deaths7 deaths6
rename deaths8 deaths7
//add "unexplained"
qui egen temp=rowtotal(deaths1 deaths2 deaths3 deaths4 deaths5 deaths6 deaths7)
qui gen deaths8=deaths0-temp
qui drop temp
label var deaths8 "Number of deaths not in main categories"
*qui gen midage=0.5
*replace midage=3 if agecat==1
*replace midage=agecat*5-2.5 if agecat>1
label val agecat agelbl
order ageband agecat midage year population north male deaths0 deaths1 deaths2 /*
*/ deaths3 deaths4 deaths5 deaths6 deaths7 deaths8
label var agecat "Age categories"
label var midage "Mid age"
```

```
label var year "Year"
label var deaths0 "Number of deaths"
label var population "Population denominator"
label var north "North"
label var male "Male"
sort north male year agecat
compress
save "dataset.dta", replace
```

## 02- Contour plots

```
capture program drop getall
program getall
        contplot 1981 deaths0 "All" 20
        contplot 1981 deaths1 "Accidents" 20
        contplot 1981 deaths2 "Alcohol" 20
        contplot 1981 deaths3 "Cardiovascular" 20
        contplot 1981 deaths4 "Suicide" 20
        contplot 1993 deaths5 "Drugs" 20
        contplot 1981 deaths6 "Cancer (excl breast)" 20
        contplot 1981 deaths7 "Breast cancer" 20
        contplot 1981 deaths8 "Unexplained" 20
        ***
        *contplotX 1981 deaths8 "Unexplained"


end


capture program drop contplot
```

```
program contplot
	//inputs
	local yrstr `1'
	local dvar "`2'"
	local cause "`3'"
	/*threshold of total deaths within year above which models are run*/
	local lmt `4'


	set maxiter 50
	set more off
	tempfile tempf
	local dir1 "P:\Evan\Informatics\North-South divide\specific-causes\"
	local dir2 "`dir1'\graphs\"
	cd "`dir2'"
	local midagelst="0.5 3 7.5 12.5 17.5 22.5 27.5 32.5 37.5 42.5 47.5 52.5 57.5 62.5 67.5
72.5 77.5 82.5 87.5"
	matrix mat1 = J(19,2,.)
	matrix colnames mat1 = IRRpeople SEpeople
	matrix mat2 = J(19,2,.)
	matrix colnames mat2 = IRRmale SEmale
	matrix mat3 = J(19,2,.)
	matrix colnames mat3 = IRRfemale SEfemale
	//get data
	forvalues yr=`yrstr'(1)2016 {
		qui use "`dir1'dataset.dta",clear
		forvalues y=1(1)`=wordcount("`midagelst'")' {
```

```
local lmage = word("`midagelst'",`y')
//both sexes
qui sum `dvar' if midage==`lmage' & year==`yr'
if r(sum)>=`lmt' & r(sum)!=. {
	qui poisson `dvar' north if midage==`lmage' & year==`yr', exposure(population)
	matrix mat1[`y',1]=exp(_b[north])
	matrix mat1[`y',2]=exp(_b[north])*_se[north]
}
else {
	matrix mat1[`y',1]=exp(0)
	matrix mat1[`y',2]=exp(0)
}
//male
qui sum `dvar' if midage==`lmage' & year==`yr' & male==1
if r(sum)>=`lmt' & r(sum)!=. {
	qui poisson `dvar' north if midage==`lmage' & year==`yr' & male==1, exposure(population)
	matrix mat2[`y',1]=exp(_b[north])
	matrix mat2[`y',2]=exp(_b[north])*_se[north]
}
else {
	matrix mat2[`y',1]=exp(0)
	matrix mat2[`y',2]=exp(0)
}
//female
qui sum `dvar' if midage==`lmage' & year==`yr' & male==0
```

```
			if r(sum)>=`lmt' & r(sum)!=. {
				qui poisson `dvar' north if midage==`lmage' & year==`yr' &
male==0, exposure(population)
				matrix mat3[`y',1]=exp(_b[north])
				matrix mat3[`y',2]=exp(_b[north])*_se[north]
			}
			else {
				matrix mat3[`y',1]=exp(0)
				matrix mat3[`y',2]=exp(0)
			}
		}
		drop _all
		qui svmat mat1, names(col)
		qui svmat mat2, names(col)
		qui svmat mat3, names(col)
		qui egen agecat=seq()
		qui gen year=`yr'
		capture save `tempf'
		if _rc!=0 {
			append using `tempf'
			qui save `tempf', replace
		}
	}
	qui use `tempf', clear

	//editing and labelling
```

```
label var agecat "Age category"
label define agelbl 1 "<1" 2 "01-04" 3 "05-09" 4 "10-14" 5 "15-19" 6 "20-24" 7 "25-29" /*
*/ 8 "30-34" 9 "35-39" 10 "40-44" 11 "45-49" 12 "50-54" 13 "55-59" 14 "60-64" 15 "65-69" /*
*/ 16 "70-74" 17 "75-79" 18 "80-84" 19 "85+"
label val agecat agelbl
foreach x in IRRpeople IRRmale IRRfemale {
	qui replace `x'=100*(`x'-1)
}
label var IRRpeople "% all excess mortality in the North"
label var IRRmale "% male excess mortality in the North"
label var IRRfemale "% female excess mortality in the North"

local ntlbl=""
if "`cause'"=="All" {
	local ccstr "-20(10)70"
}
else if "`cause'"=="Accidents" {
	local ccstr "-50(50)300"
}
else if "`cause'"=="Alcohol" {
	local ccstr "-50(50)300"
}
else if "`cause'"=="Cardiovascular" {
	local ccstr "-50(50)300"
	*local ntlbl="including diabetes mellitus and obesity"
```

```
	}
	else if "`cause'"=="Cancer (excl breast)" {
		local ccstr "-60(30)150"
	}
	else if "`cause'"=="Drugs" {
		local ccstr "-50(50)200"
	}
	else if "`cause'"=="Breast cancer" {
		local ccstr "-30(30)180"
	}
	else if "`cause'"=="Suicide" {
		local ccstr "-40(20)80"
	}
	else {
		local ccstr "-50(50)200"
	}



	//all, males and females
	foreach x in people male female {
		*twoway contour IRR`x' agecat year, zlabel(#10) format(%5.0f)
		twoway contour IRR`x' agecat year, ccuts(`ccstr') format(%5.0f) ///
		xscale(range(`yrstr' 2016)) xlabel(`yrstr'(5)2011 2016,angle(45) labsize(3)) xtitle("Year", size(medlarge)) ///
		yscale(range(1 19)) ylabel(1(1)19,valuelabel angle(0) labsize(2.4)) ytitle("Age category", size(medlarge)) ///
		, legend(off) ///
```

```
		note("`ntlbl'",size(small))
		/*
		|| function y=int((x-`yrstr'+3)/5)+3, range(`yrstr' 2016) color(black) 
lstyle(foreground) clwidth(*0.5) ///
		|| function y=int((x-`yrstr'+3)/5)+5, range(`yrstr' 2016) color(black) 
lstyle(foreground) clwidth(*0.5) ///
		|| function y=int((x-`yrstr'+3)/5)+7, range(`yrstr' 2016) color(black) 
lstyle(foreground) clwidth(*0.5) ///
		|| function y=int((x-`yrstr'+3)/5)+9, range(`yrstr' 2016) color(black) 
lstyle(foreground) clwidth(*0.5) ///
		|| function y=int((x-`yrstr'+3)/5)+11, range(`yrstr' 2016) color(black) 
lstyle(foreground) clwidth(*0.5) ///
		|| function y=int((x-`yrstr'+3)/5)+13, range(`yrstr' 2014) color(black) 
lstyle(foreground) clwidth(*0.5) ///
		|| function y=int((x-`yrstr'+3)/5)+15, range(`yrstr' 2004) color(black) 
lstyle(foreground) clwidth(*0.5) ///
		|| function y=int((x-`yrstr'+3)/5)+17, range(`yrstr' 1994) color(black) 
lstyle(foreground) clwidth(*0.5) ///
		*|| function y=int((x-`yrstr')/5)+1, range(`yrstr' 2016) color(black) 
lstyle(foreground) clwidth(*0.5) ///
		*|| function y=int((x-`yrstr')/5)-1, range(1975 2016) color(black) 
lstyle(foreground) clwidth(*0.5) ///
		*|| function y=int((x-`yrstr')/5)-3, range(1985 2016) color(black) 
lstyle(foreground) clwidth(*0.5) ///
		*|| function y=int((x-`yrstr')/5)-5, range(1995 2016) color(black) 
lstyle(foreground) clwidth(*0.5) ///
		*|| function y=int((x-`yrstr')/5)-7, range(2005 2016) color(black) 
lstyle(foreground) clwidth(*0.5)
		*/
		graph export "2-contour_`cause'_`x'.png", replace
		graph export "2-contour_`cause'_`x'.eps", replace
```

```
	}
end

//contour plot to show "unexplained" across ages, aggregated across north and south
capture program drop contplotX
program contplotX
	//inputs
	local yrstr `1'
	local dvar "`2'"
	local cause "`3'"

	set maxiter 50
	set more off
	tempfile tempf
	local dir1 "P:\Evan\Informatics\North-South divide\specific-causes\"
	local dir2 "`dir1'\graphs\"
	cd "`dir2'"
	local midagelst="0.5 3 7.5 12.5 17.5 22.5 27.5 32.5 37.5 42.5 47.5 52.5 57.5 62.5 67.5
72.5 77.5 82.5 87.5"
	matrix mat1 = J(19,2,.)
	matrix colnames mat1 = PRCpeople SEpeople
	matrix mat2 = J(19,2,.)
	matrix colnames mat2 = PRCmale SEmale
	matrix mat3 = J(19,2,.)
	matrix colnames mat3 = PRCfemale SEfemale
	//get data
```

```
forvalues yr=`yrstr'(1)2016 {
	qui use "`dir1'dataset.dta",clear
	forvalues y=1(1)`=wordcount("`midagelst'")' {
		local lmage = word("`midagelst'",`y')
		//both sexes
		qui sum `dvar' if midage==`lmage' & year==`yr'
		local tnum=r(sum)
		qui sum deaths0 if midage==`lmage' & year==`yr'
		matrix mat1[`y',1]= `tnum'/r(sum)
		matrix mat1[`y',2]=sqrt((mat1[`y',1]*(1-mat1[`y',1]))/r(sum))
		//male
		qui sum `dvar' if midage==`lmage' & year==`yr' & male==1
		local tnum=r(sum)
		qui sum deaths0 if midage==`lmage' & year==`yr' & male==1
		matrix mat2[`y',1]= `tnum'/r(sum)
		matrix mat2[`y',2]=sqrt((mat2[`y',1]*(1-mat2[`y',1]))/r(sum))
		//female
		qui sum `dvar' if midage==`lmage' & year==`yr' & male==0
		local tnum=r(sum)
		qui sum deaths0 if midage==`lmage' & year==`yr' & male==0
		matrix mat3[`y',1]= `tnum'/r(sum)
		matrix mat3[`y',2]=sqrt((mat3[`y',1]*(1-mat3[`y',1]))/r(sum))
	}
	drop _all
	qui svmat mat1, names(col)
	qui svmat mat2, names(col)
```

```
		qui svmat mat3, names(col)
		qui egen agecat=seq()
		qui gen year=`yr'
		capture save `tempf'
		if _rc!=0 {
			append using `tempf'
			qui save `tempf', replace
		}
	}
	qui use `tempf', clear


	//editing and labelling
	label var agecat "Age category"
	label define agelbl 1 "<1" 2 "01-04" 3 "05-09" 4 "10-14" 5 "15-19" 6 "20-24" 7 "25-29"
/*
	*/ 8 "30-34" 9 "35-39" 10 "40-44" 11 "45-49" 12 "50-54" 13 "55-59" 14 "60-64" 15
"65-69" /*
	*/ 16 "70-74" 17 "75-79" 18 "80-84" 19 "85+"
	label val agecat agelbl
	foreach x in PRCpeople PRCmale PRCfemale {
		qui replace `x'=100*`x'
	}
	label var PRCpeople "% over all deaaths for England: all people"
	label var PRCmale "% over all deaths for England: males"
	label var PRCfemale "% over all deaths for England: females"


	//all, males and females
```

```
	foreach x in people male female {
		twoway contour PRC`x' agecat year, zlabel(#10) format(%5.0f) ///
		xscale(range(`yrstr' 2016)) xlabel(`yrstr'(5)2011 2016,angle(45) labsize(3)) xtitle("Year", size(medlarge)) ///
		yscale(range(1 19)) ylabel(1(1)19,valuelabel angle(0) labsize(2.4)) ytitle("Age category", size(medlarge)) ///
		, legend(off)
		graph export "2-contourX_`cause'_`x'.png", replace
		graph export "2-contourX_`cause'_`x'.pdf", replace
	}
end
```

## 03- Raw plots

```
capture program drop getall
program getall
	foreach x in people male female {
		rawplots 1981 deaths0 "All" `x'
		rawplots 1981 deaths1 "Accidents" `x'
		rawplots 1981 deaths2 "Alcohol" `x'
		rawplots 1981 deaths3 "Cardiovascular" `x'
		rawplots 1981 deaths4 "Suicide" `x'
		rawplots 1981 deaths5 "Drugs" `x'
		rawplots 1981 deaths6 "Cancer (excl breast)" `x'
		rawplots 1993 deaths7 "Breast cancer" `x'
		rawplots 1981 deaths8 "Unexplained" `x'
	}
end
```

```
capture program drop rawplots

program rawplots

	//inputs

	local yrstr `1'

	local dvar "`2'"

	local cause "`3'"

	local sex "`4'"

	set more off

	local dir1 "P:\Evan\Informatics\North-South divide\specific-causes\"

	local dir2 "`dir1'\graphs\"

	local dir3 "`dir1'\excel\"

	capture mkdir "`dir3'"

	cd "`dir2'"

	qui use "`dir1'dataset.dta", clear


	//gen some needed variables

	qui {

		qui drop if midage>=75

		if "`sex'"=="male" {

			keep if male==1

		}

		else if "`sex'"=="female" {

			keep if male==0

		}

		collapse (sum) `dvar' population, by(year north)

		gen drate=`dvar'/population*100
```

```
		gen sterror=sqrt(drate*(100-drate)/population)
		gen l95CI=drate-1.96*sterror
		gen u95CI=drate+1.96*sterror
		keep if year>=`yrstr'
	}

	//raw plots with CI
	//to shade between xlines
	qui sum u95CI
	local max=round(r(max),.01)
	if `max'<r(max) {
		local max=`max'+.01
	}
	*qui sum l95CI
	*local min=round(r(min),.01)
	qui gen c3=`max' if (year>=1990 & year<=1991)
	qui gen c4=`max' if (year>=2008 & year<=2009)
	if `yrstr'<=1990 {
		graph twoway area c3 year, bcolor(sand) || area c4 year, bcolor(sand) /*

		*/ || rarea l95CI u95CI year if north==0, color(gs14) || rarea l95CI u95CI year if north==1, color(gs14) /*
		*/ || line drate year if north==0, lcolor(blue) || line drate year if north==1, lcolor(maroon) /*
		*/ legend(order(5 "South(`sex')" 6 "North(`sex')" 3 "95% CI" 1 "Recession periods")) /*
		*yscale(range(0,`max'))
		*/ ytitle("% crude mortality aged{&le}75: `cause'", size(small)) /*
```

```
		*/ xtitle("Year") /*
		*/ ylabel(, format(%4.3f) labsize(2.5) angle(horizontal)) /*
		*/ xlabel(1981 1985 1990 1995 2000 2005 2010 2015 2016, labsize(2.2) angle(45)) /*
		*/ xsize(6) ysize(4)
	}
	else {
		graph twoway area c4 year, bcolor(sand) /*
		*/ || rarea l95CI u95CI year if north==0, color(gs14) || rarea l95CI u95CI year if north==1, color(gs14) /*
		*/ || line drate year if north==0, lcolor(blue) || line drate year if north==1, lcolor(maroon) /*
		*/ legend(order(4 "South(`sex')" 5 "North(`sex')" 2 "95% CI" 1 "Recession periods")) /*
		*yscale(range(0,`=round(`max',.01)'))
		*/ ytitle("% crude mortality aged{&le}75: `cause'", size(small)) /*
		*/ xtitle("Year") /*
		*/ ylabel(, format(%4.3f) labsize(2.5) angle(horizontal)) /*
		*/ xlabel(1993 1995 2000 2005 2010 2015 2016, labsize(2.2) angle(45)) /*
		*/ xsize(6) ysize(4)
	}

	graph export "3-Crude_`cause'_`sex'_byregion.png", replace
	graph export "3-Crude_`cause'_`sex'_byregion.eps", replace
	graph export "3-Crude_`cause'_`sex'_byregion.pdf", replace
	window manage close graph
	preserve
	qui keep year north	`dvar' population drate		sterror l95CI u95CI
```

```
	*local xcause=subinstr("`cause'"," ","_",.)
	if "`cause'"=="Cardiovascular" local cause="Cardio"
	qui export excel "`dir3'graphexport.xlsx", firstrow(variables) 
sheet("Crude_`xcause'_`sex'") sheetreplace
	restore
end
```

## 04-SMR plots

```
capture program drop getall
program getall
	foreach x in people male female {
		forvalues i=0(1)1 {
			smrplots `i' 1981 deaths0 "All" `x'
			*smrplots `i' 1981 deaths1 "Accidents" `x'
			*smrplots `i' 1981 deaths2 "Alcohol" `x'
			*smrplots `i' 1981 deaths3 "Cardiovascular" `x'
			*smrplots `i' 1981 deaths4 "Suicide" `x'
			*smrplots `i' 1981 deaths5 "Drugs" `x'
			*smrplots `i' 1981 deaths6 "Cancer (excl breast)" `x'
			*smrplots `i' 1993 deaths7 "Breast cancer" `x'
			*smrplots `i' 1981 deaths8 "Unexplained" `x'
		}
	}
	//combine graphs by gender
	foreach x in "All" /*"Accidents" "Alcohol" "Cardiovascular" "Suicide" "Drugs" /*
	*/ "Cancer (excl breast)" "Breast cancer" "Unexplained"*/ {
		foreach y in /*"undr75"*/ "25t044" {
```

```
			combgraph `y' `x'
		}
	}
end


capture program drop smrplots
program smrplots
	//inputs
	local tp "`1'"
	local yrstr `2'
	local dvar "`3'"
	local cause "`4'"
	local sex "`5'"
	set more off
	local dir1 "P:\Evan\Informatics\North-South divide\specific-causes\"
	local dir2 "`dir1'\graphs\"
	local dir3 "`dir1'\excel\"
	local dir4 "`dir1'\SMR\"
	capture mkdir "`dir3'"
	capture mkdir "`dir4'"
	cd "`dir2'"
	qui use "`dir1'dataset.dta", clear


	//calculate SMRs (north and south)
	qui {
		if `tp'==0 {
```

```
}
        qui drop if midage>=75
        local scat=0
        local ecat=15
        local tmpstr "aged{&le}75"
        local tmpstx "undr75"
        if "`cause'"=="Suicide" {
                qui drop if midage<15
                local scat=4
                local tmpstr "15{&le}aged{&le}75"
        }
}
else if `tp'==1 {
        qui drop if midage>44 | midage<25
        local scat=6
        local ecat=9
        local tmpstr "25{&le}aged{&le}44"
        local tmpstx "25t044"
}
if "`sex'"=="male" {
        keep if male==1
}
else if "`sex'"=="female" {
        keep if male==0
}
```

***

```
keep if year>=`yrstr'

keep `dvar' population year male north agecat

//for whole population

if "`sex'"!="male" & "`sex'"!="female" {

        rename `dvar' `dvar'_

        rename population population_

        reshape wide `dvar'@_ population@_, i(year male north) j(agecat)


        reshape wide `dvar'* population*, i(year north) j(male)

        //calculate mortality counts across all England, to relate to 100k
deaths (Add up to 100k)

        scalar fden=0

        scalar mden=0

        forvalues i=`scat'(1)`ecat' {

                //numerators

                qui sum population`i'_0

                scalar num`i'f=r(sum)

                qui sum population`i'_1

                scalar num`i'm=r(sum)

                //denominators

                scalar fden = fden + num`i'f

                scalar mden = mden + num`i'm

        }

        forvalues i=`scat'(1)`ecat' {

                //not rounding this time

                scalar p`i'_0=(100000*num`i'f/fden)

                scalar p`i'_1=(100000*num`i'm/mden)
```

```
			}
			//calculate SMR into variable
			qui gen SMR=0
			foreach x in 0 1 {
				forvalues i=`scat'(1)`ecat' {
					qui replace
SMR=SMR+((`dvar'`i'_`x'/population`i'_`x')*p`i'_`x')/2
				}
			}
			label var SMR "SMR, by agegroup and sex: `cause'"
		}
		//for males-females
		else {
			reshape wide `dvar'@ population@, i(year male north) j(agecat)
			//calculate mortality counts across all England, to relate to 100k
deaths (Add up to 100k)
			scalar fden=0
			forvalues i=`scat'(1)`ecat' {
				//numerator
				qui sum population`i'
				scalar num`i'f=r(sum)
				//denominator
				scalar fden = fden + num`i'f
			}
			forvalues i=`scat'(1)`ecat' {
				//not rounding this time
				scalar p`i'=(100000*num`i'f/fden)
```

```
            }
            //calculate SMR into variable
            qui gen SMR=0
            forvalues i=`scat'(1)`ecat' {
                        qui replace SMR=SMR+((`dvar'`i'/population`i')*p`i')
            }
            label var SMR "SMR, by agegroup for `sex's: `cause'"
        }

        qui egen allD=rowtotal(`dvar'*)
        label var allD "All deaths: `cause'"
        /*calculate CI for SMR*/
        qui gen SMRlo = SMR - invnormal(.975)*SMR/sqrt(allD)
        label var SMRlo "SMR lower 95% CI"
        qui gen SMRup = SMR + invnormal(.975)*SMR/sqrt(allD)
        label var SMRup "SMR upper 95% CI"
        order year north allD SMR*
        qui compress
        *note that for Suicide the SMR is for aged 15 to 75
        save "`dir4'SMR`tmpstx'_`cause'_`sex'.dta", replace
}

//SMR plots with CI
qui sum SMRup
if `=round(ceil(r(max)),10)'>r(max) {
        local smax=round(ceil(r(max)),10)
```

```
	}
	else {
		local smax=round(ceil(r(max))+10,10)
	}
	qui sum SMRlo
	local smin=max(round(floor(r(min)),100),0)
	qui gen c3=`smax' if (year>=1990 & year<=1991)
	qui gen c4=`smax' if (year>=2008 & year<=2009)
	if `yrstr'<=1990 {
		graph twoway /*area c3 year, bcolor(sand) || area c4 year, bcolor(sand) */ /*

		*/ || rarea SMRlo SMRup year if north==0, color(gs14) || rarea SMRlo SMRup
year if north==1, color(gs14) /*
		*/ || line SMR year if north==0, lcolor(blue) || line SMR year if north==1,
lcolor(maroon) /*
		*/ legend(order(3 4 1)) /*
		*/ legend(rows(1) size(2.4) label(3 "South") label(4 "North") label(1 "95%
CI"))/*
		*/ yscale(range(0)) /*
		*/ ytitle(`"`=strproper("`sex'")' SMR `tmpstr' (per 100,000): `cause'"',
size(small)) /*
		*/ xtitle("Year") /*
		*/ ylabel(#6, format(%3.0f) labsize(2.5) angle(horizontal)) /*
		*/ xlabel(1981 1985 1990 1995 2000 2005 2010 2015 2016, labsize(2.2)
angle(45)) /*
		*/ xsize(6) ysize(4)
	}
	else {
```

```
		graph twoway /*area c4 year, bcolor(sand) ||*/ /*
		*/ rarea SMRlo SMRup year if north==0, color(gs14) || rarea SMRlo SMRup year if north==1, color(gs14) /*
		*/ || line SMR year if north==0, lcolor(blue) || line SMR year if north==1, lcolor(maroon) /*
		*/ legend(order(3 4 1)) /*
		*/ legend(rows(1) size(2.4) label(3 "South") label(4 "North") label(1 "95% CI"))/*
		*/ yscale(range(0)) /*
		*/ ytitle(`"`=strproper("`sex'")' SMR `tmpstr' (per 100,000): `cause'"', size(small)) /*
		*/ xtitle("Year") /*
		*/ ylabel(#6, format(%3.0f) labsize(2.5) angle(horizontal)) /*
		*/ xlabel(1993 1995 2000 2005 2010 2015 2016, labsize(2.2) angle(45)) /*
		*/ xsize(6) ysize(4)
	}

	graph export "4-SMR_`tmpstx'_`cause'_`sex'_byregion.png", replace
	graph export "4-SMR_`tmpstx'_`cause'_`sex'_byregion.eps", replace
	*graph export "4-SMR_`tmpstx'_`cause'_`sex'_byregion.pdf", replace
	graph save "4-SMR_`tmpstx'_`cause'_`sex'_byregion.gph", replace
	window manage close graph
	preserve
	local causx="`cause'"
	if "`causx'"=="Cardiovascular" {
		local causx="Cardio"
	}
	if "`causx'"=="Cancer (excl breast)" {
```

```
			local causx="Cancer-B"
		}
		if "`causx'"=="Breast cancer" {
			local causx="BreastC"
		}
		qui export excel "`dir3'graphexport.xlsx", firstrow(variables)
sheet("SMR_`tmpstx'_`causex'_`sex'") sheetreplace
		restore
end


/*combines graphs into a single file to be used in publication - one legend*/
capture program drop combgraph
program combgraph
		//inputs
		local tmpstx="`1'"
		local cause="`2'"
		local dir1 "P:\Evan\Informatics\North-South divide\specific-causes\"
		local dir2 "`dir1'\graphs\"
		cd "`dir2'"
		local dir3 "P:\Evan\Informatics\North-South divide\specific-causes\paper\graphs\"
   //findit grc1leg2
   grc1leg2 "4-SMR_`tmpstx'_`cause'_female_byregion.gph" "4-
SMR_`tmpstx'_`cause'_male_byregion.gph", /*
   */ cols(1) imargin(small) legendfrom("4-SMR_`tmpstx'_`cause'_female_byregion.gph")
/*lsize(2.0)*/ ysize(8) xsize(5.5)
   graph display, ysize(8) xsize(5.5)
   graph export "`dir3'Fig3.eps", replace
```

```
	graph export "`dir3'Fig3.png", replace width(1200)
end
```

## 05-Cross-sectional crude

```
capture program drop getall
program getall
	foreach x in people male female {
		forvalues i=0(1)1 {
			smrplots `i' 1981 deaths0 "All" `x'
			*smrplots `i' 1981 deaths1 "Accidents" `x'
			*smrplots `i' 1981 deaths2 "Alcohol" `x'
			*smrplots `i' 1981 deaths3 "Cardiovascular" `x'
			*smrplots `i' 1981 deaths4 "Suicide" `x'
			*smrplots `i' 1981 deaths5 "Drugs" `x'
			*smrplots `i' 1981 deaths6 "Cancer (excl breast)" `x'
			*smrplots `i' 1993 deaths7 "Breast cancer" `x'
			*smrplots `i' 1981 deaths8 "Unexplained" `x'
		}
	}
	//combine graphs by gender
	foreach x in "All" /*"Accidents" "Alcohol" "Cardiovascular" "Suicide" "Drugs" /*
	*/ "Cancer (excl breast)" "Breast cancer" "Unexplained"*/ {
		foreach y in /*"undr75"*/ "25t044" {
			combgraph `y' `x'
		}
	}
end
```

```
capture program drop smrplots
program smrplots
	//inputs
	local tp "`1'"
	local yrstr `2'
	local dvar "`3'"
	local cause "`4'"
	local sex "`5'"
	set more off
	local dir1 "P:\Evan\Informatics\North-South divide\specific-causes\"
	local dir2 "`dir1'\graphs\"
	local dir3 "`dir1'\excel\"
	local dir4 "`dir1'\SMR\"
	capture mkdir "`dir3'"
	capture mkdir "`dir4'"
	cd "`dir2'"
	qui use "`dir1'dataset.dta", clear

	//calculate SMRs (north and south)
	qui {
		if `tp'==0 {
			qui drop if midage>=75
			local scat=0
			local ecat=15
			local tmpstr "aged{&le}75"
```

```
		local tmpstx "undr75"
		if "`cause'"=="Suicide" {
			qui drop if midage<15
			local scat=4
			local tmpstr "15{&le}aged{&le}75"
		}
	}
	else if `tp'==1 {
		qui drop if midage>44 | midage<25
		local scat=6
		local ecat=9
		local tmpstr "25{&le}aged{&le}44"
		local tmpstx "25t044"
	}
	if "`sex'"=="male" {
		keep if male==1
	}
	else if "`sex'"=="female" {
		keep if male==0
	}

	***
	keep if year>=`yrstr'
	keep `dvar' population year male north agecat
	//for whole population
	if "`sex'"!="male" & "`sex'"!="female" {
```

```
rename `dvar' `dvar'_

rename population population_

reshape wide `dvar'@_ population@_, i(year male north) j(agecat)


reshape wide `dvar'* population*, i(year north) j(male)

//calculate mortality counts across all England, to relate to 100k deaths (Add up to 100k)

scalar fden=0

scalar mden=0

forvalues i=`scat'(1)`ecat' {

    //numerators

    qui sum population`i'_0

    scalar num`i'f=r(sum)

    qui sum population`i'_1

    scalar num`i'm=r(sum)

    //denominators

    scalar fden = fden + num`i'f

    scalar mden = mden + num`i'm

}

forvalues i=`scat'(1)`ecat' {

    //not rounding this time

    scalar p`i'_0=(100000*num`i'f/fden)

    scalar p`i'_1=(100000*num`i'm/mden)

}

//calculate SMR into variable

qui gen SMR=0

foreach x in 0 1 {
```

```
				forvalues i=`scat'(1)`ecat' {

					qui replace
SMR=SMR+((`dvar'`i'_`x'/population`i'_`x')*p`i'_`x')/2

				}

			}

			label var SMR "SMR, by agegroup and sex: `cause'"

		}

		//for males-females

		else {

			reshape wide `dvar'@ population@, i(year male north) j(agecat)

			//calculate mortality counts across all England, to relate to 100k
deaths (Add up to 100k)

			scalar fden=0

			forvalues i=`scat'(1)`ecat' {

				//numerator

				qui sum population`i'

				scalar num`i'f=r(sum)

				//denominator

				scalar fden = fden + num`i'f

			}

			forvalues i=`scat'(1)`ecat' {

				//not rounding this time

				scalar p`i'=(100000*num`i'f/fden)

			}

			//calculate SMR into variable

			qui gen SMR=0

			forvalues i=`scat'(1)`ecat' {
```

```
				qui replace SMR=SMR+((`dvar'`i'/population`i')*p`i')
			}
			label var SMR "SMR, by agegroup for `sex's: `cause'"
		}

		qui egen allD=rowtotal(`dvar'*)
		label var allD "All deaths: `cause'"
		/*calculate CI for SMR*/
		qui gen SMRlo = SMR - invnormal(.975)*SMR/sqrt(allD)
		label var SMRlo "SMR lower 95% CI"
		qui gen SMRup = SMR + invnormal(.975)*SMR/sqrt(allD)
		label var SMRup "SMR upper 95% CI"
		order year north allD SMR*
		qui compress
		*note that for Suicide the SMR is for aged 15 to 75
		save "`dir4'SMR`tmpstx'_`cause'_`sex'.dta", replace
	}

	//SMR plots with CI
	qui sum SMRup
	if `=round(ceil(r(max)),10)'>r(max) {
		local smax=round(ceil(r(max)),10)
	}
	else {
		local smax=round(ceil(r(max))+10,10)
	}
```

```
qui sum SMRlo

local smin=max(round(floor(r(min)),100),0)

qui gen c3=`smax' if (year>=1990 & year<=1991)

qui gen c4=`smax' if (year>=2008 & year<=2009)

if `yrstr'<=1990 {

	graph twoway /*area c3 year, bcolor(sand) || area c4 year, bcolor(sand) */ /*

	*/ || rarea SMRlo SMRup year if north==0, color(gs14) || rarea SMRlo SMRup year if north==1, color(gs14) /*

	*/ || line SMR year if north==0, lcolor(blue) || line SMR year if north==1, lcolor(maroon) /*

	*/ legend(order(3 4 1)) /*

	*/ legend(rows(1) size(2.4) label(3 "South") label(4 "North") label(1 "95% CI"))/*

	*/ yscale(range(0)) /*

	*/ ytitle(`"`=strproper("`sex'")' SMR `tmpstr' (per 100,000): `cause'"', size(small)) /*

	*/ xtitle("Year") /*

	*/ ylabel(#6, format(%3.0f) labsize(2.5) angle(horizontal)) /*

	*/ xlabel(1981 1985 1990 1995 2000 2005 2010 2015 2016, labsize(2.2) angle(45)) /*

	*/ xsize(6) ysize(4)

}

else {

	graph twoway /*area c4 year, bcolor(sand) ||*/ /*

	*/ rarea SMRlo SMRup year if north==0, color(gs14) || rarea SMRlo SMRup year if north==1, color(gs14) /*

	*/ || line SMR year if north==0, lcolor(blue) || line SMR year if north==1, lcolor(maroon) /*
```

```
		*/ legend(order(3 4 1)) /*
		*/ legend(rows(1) size(2.4) label(3 "South") label(4 "North") label(1 "95%
CI"))/*
		*/ yscale(range(0)) /*
		*/ ytitle(`"`=strproper("`sex'")' SMR `tmpstr' (per 100,000): `cause'"',
size(small)) /*
		*/ xtitle("Year") /*
		*/ ylabel(#6, format(%3.0f) labsize(2.5) angle(horizontal)) /*
		*/ xlabel(1993 1995 2000 2005 2010 2015 2016, labsize(2.2) angle(45)) /*
		*/ xsize(6) ysize(4)
	}

	graph export "4-SMR_`tmpstx'_`cause'_`sex'_byregion.png", replace
	graph export "4-SMR_`tmpstx'_`cause'_`sex'_byregion.eps", replace
	*graph export "4-SMR_`tmpstx'_`cause'_`sex'_byregion.pdf", replace
	graph save "4-SMR_`tmpstx'_`cause'_`sex'_byregion.gph", replace
	window manage close graph
	preserve
	local causx="`cause'"
	if "`causx'"=="Cardiovascular" {
		local causx="Cardio"
	}
	if "`causx'"=="Cancer (excl breast)" {
		local causx="Cancer-B"
	}
	if "`causx'"=="Breast cancer" {
		local causx="BreastC"
```

```
	}
	qui export excel "`dir3'graphexport.xlsx", firstrow(variables)
sheet("SMR_`tmpstx'_`causex'_`sex'") sheetreplace
	restore
end


/*combines graphs into a single file to be used in publication - one legend*/
capture program drop combgraph
program combgraph
	//inputs
	local tmpstx="`1'"
	local cause="`2'"
	local dir1 "P:\Evan\Informatics\North-South divide\specific-causes\"
	local dir2 "`dir1'\graphs\"
	cd "`dir2'"
	local dir3 "P:\Evan\Informatics\North-South divide\specific-causes\paper\graphs\"
   //findit grc1leg2
   grc1leg2 "4-SMR_`tmpstx'_`cause'_female_byregion.gph" "4-
SMR_`tmpstx'_`cause'_male_byregion.gph", /*
   */ cols(1) imargin(small) legendfrom("4-SMR_`tmpstx'_`cause'_female_byregion.gph")
/*lsize(2.0)*/ ysize(8) xsize(5.5)
   graph display, ysize(8) xsize(5.5)
   graph export "`dir3'Fig3.eps", replace
	graph export "`dir3'Fig3.png", replace width(1200)
end
```

## 06- cross-sectional IRR

```
capture program drop getall
program getall
	*irrplot 25 44 all 20
	irrplot 25 44 males 20
	irrplot 25 44 females 20
	*irrplot 0 75 all 20
	*irrplot 0 75 males 20
	*irrplot 0 75 females 20
	//combine graphs
	combgraph 25 44
end

capture program drop irrplot
program irrplot
	//inputs
	local xstr=`1'
	local xend=`2'
	local grp="`3'"
	/*threshold of total deaths within year above which models are run*/
	local lmt `4'

	***
	set maxiter 50
	set more off
	tempfile tempf tempg
```

```
local dir1 "P:\Evan\Informatics\North-South divide\specific-causes\"
local dir2 "`dir1'\graphs\"
cd "`dir2'"
//strings and matrices
local midagelst="0.5 3 7.5 12.5 17.5 22.5 27.5 32.5 37.5 42.5 47.5 52.5 57.5 62.5 67.5 72.5 77.5 82.5 87.5"
matrix mat1 = J(19,5,.)
matrix colnames mat1 = IRR SE EXD deathsN midage
//load data
qui use "`dir1'dataset.dta",clear
qui keep if inlist(year,2014,2015,2016)
label define nslbl 0 "South" 1 "North"
label val north nslbl
//keep data of interest
if "`grp'"=="males" {
	qui keep if male==1
}
if "`grp'"=="females" {
	qui keep if male==0
}
qui collapse (sum) deaths0-deaths8, by(ageband agecat midage north population)
qui save `tempg', replace


//loop on death group
forvalues i=1(1)8 {
	qui use `tempg', clear
```

```
		local dvar "deaths`i'"
		//loop on age group
		forvalues y=1(1)`=wordcount("`midagelst'")' {
			local lmage = word("`midagelst'",`y')
			//model
			qui sum `dvar' if midage==`lmage'
			if r(sum) > `lmt' {
				qui poisson `dvar' north if midage==`lmage',
exposure(population)
				matrix mat1[`y',1]=exp(_b[north])
				matrix mat1[`y',2]=exp(_b[north])*_se[north]
				qui sum `dvar' if midage==`lmage' & north==1
				matrix mat1[`y',3]=round(r(sum)*(1-1/mat1[`y',1]))
			}
			else {
				matrix mat1[`y',1]=1
				matrix mat1[`y',2]=.
				matrix mat1[`y',3]=0
			}
			qui sum `dvar' if midage==`lmage' & north==1
			matrix mat1[`y',4]=r(sum)
			matrix mat1[`y',5]=`lmage'
		}
		drop _all
		qui svmat mat1, names(col)
		qui egen agecat=seq()
```

```
		qui gen deaths=`i'
		capture save `tempf'
		if _rc!=0 {
			append using `tempf'
			qui save `tempf', replace
		}
	}
	qui use `tempf', clear

	//editing and labelling
	label var agecat "Age category"
	label define agelbl 1 "<1" 2 "01-04" 3 "05-09" 4 "10-14" 5 "15-19" 6 "20-24" 7 "25-29"
/*
	*/ 8 "30-34" 9 "35-39" 10 "40-44" 11 "45-49" 12 "50-54" 13 "55-59" 14 "60-64" 15
"65-69" /*
	*/ 16 "70-74" 17 "75-79" 18 "80-84" 19 "85+"
	label val agecat agelbl
	label var EXD "Excess deaths in the North"
	qui save `tempf', replace

	//make some edits and save as dataset to report in appendix
	qui egen id=seq(), from(1) to(19)
	qui keep if midage>=`xstr' & midage<=`xend'
	qui gen EXD_lo=round(deathsN*(1-1/(IRR-invnormal(0.975)*SE)))
	qui gen EXD_up=round(deathsN*(1-1/(IRR+invnormal(0.975)*SE)))
	qui gen IRR_lo=IRR-invnormal(0.975)*SE
	qui gen IRR_up=IRR+invnormal(0.975)*SE
```

```
qui keep EXD* id midage deaths agecat IRR*
qui gen north=1
label define nslbl 0 "South" 1 "North"
label val north nslbl
keep agecat deaths EXD* IRR*
sort agecat deaths
label var EXD "Excess number of deaths"
label var IRR "Incidence rate ratio"
label define dthlbl 1 "Accidents" 2 "Alcohol" 3 "Cardiovascular" 4 "Suicide" ///
5 "Drugs" 6 "Cancer (excl breast)" 7 "Breast cancer" 8 "Other"
label var deaths "Death type"
label val deaths dthlbl
qui egen id=seq(), from(1) to(8)
reshape wide EXD EXD_lo EXD_up IRR IRR_lo IRR_up, i(id) j(agecat)
qui compress
order id deaths
qui save "`dir1'IRR death counts `xstr'-`xend' `grp'.dta", replace


qui use `tempf', clear
//restrict data for plotting
qui egen id=seq(), from(1) to(19)
qui keep if midage>=`xstr' & midage<=`xend'
qui keep EXD id midage deaths agecat
qui gen north=1
qui replace north=0 if EXD<0
label define nslbl 0 "South" 1 "North"
```

```
label val north nslbl

qui replace EXD=-EXD if EXD<0

qui reshape wide EXD, i(id midage agecat north) j(deaths)

//make some edits following co-author comments

qui save `tempg', replace

qui keep if north==0

qui drop north

foreach x of varlist EXD1-EXD8 {

    qui replace `x'=-`x'

}

qui merge 1:m midage agecat id using `tempg', replace update

qui drop if north==0

qui drop north _merge


//graph: numbers

/*graph bar EXD1-EXD8, over(north, gap(*0.5) label(labsize(2.5) angle(90))) over(agecat, gap(*1.2) label(labsize(3.5) angle(45))) stack*/

graph bar EXD1-EXD8, over(agecat, gap(*1.2) label(labsize(3.5) angle(45))) stack ///

legend(row(1) size(2.4) label(1 "Accidents") label(2 "Alcohol") label(3 "Cardiovascular") label(4 "Suicide")) ///

legend(row(2) size(2.4) label(5 "Drugs") label(6 "Cancer (excl breast)") label(7 "Breast cancer") label(8 "Other")) ///

ytitle("Numbers of adjusted excess deaths, 2014-16: `grp'", size(2.5)) yscale(titlegap(*10)) ///

ylabel(0(500)1500, format(%3.0f) labsize(2.5)) ///

yscale(range(0 1500))

graph export "6-stack_IRR_`=round(`xstr')'to`=round(`xend')'_`grp'_numbers.png", replace
```

```
	graph export "6-stack_IRR_`=round(`xstr')'to`=round(`xend')'_`grp'_numbers.eps", replace
	*graph export "6-stack_IRR_`=round(`xstr')'to`=round(`xend')'_`grp'_numbers.pdf", replace
	graph save "6-stack_IRR_`=round(`xstr')'to`=round(`xend')'_`grp'_numbers.gph", replace
	window manage close graph


	//graph: percentages
	qui use `tempg', clear
	graph bar EXD1-EXD8, over(north, gap(*0.5) label(labsize(2.5) angle(90))) over(agecat, gap(*1.2) label(labsize(3.5) angle(45))) stack percentages ///
	legend(row(1) size(2.4) label(1 "Accidents") label(2 "Alcohol") label(3 "Cardiovascular") label(4 "Suicide")) ///
	legend(row(2) size(2.4) label(5 "Drugs") label(6 "Cancer (excl breast)") label(7 "Breast cancer") label(8 "Other")) ///
	ytitle("% of adjusted excess deaths, 2014-16: `grp'", size(2.5)) yscale(titlegap(*10)) ///
	ylabel(, format(%3.0f) labsize(2.5) angle(horizontal))
	graph export "6-stack_IRR_`=round(`xstr')'to`=round(`xend')'_`grp'_percentages.png", replace
	graph export "6-stack_IRR_`=round(`xstr')'to`=round(`xend')'_`grp'_percentages.eps", replace
	*graph export "6-stack_IRR_`=round(`xstr')'to`=round(`xend')'_`grp'_percentages.pdf", replace
	window manage close graph


	//generate log
	qui log using "`dir1'log6a-IRR death counts `xstr'-`xend' `grp'", name(log1) smcl replace
	qui egen total=rowtotal(EXD1-EXD8)
```

```
	list, sepby(agecat)
	foreach x of varlist EXD1-total {
		sum `x'
		di as text "total=" as result r(sum)
	}
	di _newline(2) as text "1=Accidents 2=Alcohol 3=Cardiovascular, DM and obesity 4=Suicide 5=Drugs 6=Cancer (excl breast) 7=Breast cancer 8=Other"
	//log close
	qui log close _all
	qui log2html "`dir1'log6a-IRR death counts `xstr'-`xend' `grp'", replace ti("Poisson (IRR) based death counts by reason for 2014-2016: `xstr'-`xend', `grp'")

end

/*combines graphs into a single file to be used in publication - one legend*/
capture program drop combgraph
program combgraph
	//inputs
	local xstr=`1'
	local xend=`2'
	local dir1 "P:\Evan\Informatics\North-South divide\specific-causes\"
	local dir2 "`dir1'\graphs\"
	cd "`dir2'"
	local dir3 "P:\Evan\Informatics\North-South divide\specific-causes\paper\graphs\"
   //findit grc1leg2
   grc1leg2 "6-stack_IRR_`=round(`xstr')'to`=round(`xend')'_females_numbers.gph" "6-stack_IRR_`=round(`xstr')'to`=round(`xend')'_males_numbers.gph", /*
```

```
    */ cols(1) imargin(small) legendfrom("6-
stack_IRR_`=round(`xstr')'to`=round(`xend')'_females_numbers.gph") lsize(1.7) ysize(8)
xsize(5.5)

    graph display, ysize(8) xsize(5.5)

    graph export "`dir3'Fig2.eps", replace

        graph export "`dir3'Fig2.png", replace width(1200)


end




//overall mortality output for media

capture program drop irrout

program irrout

        //inputs

        local xstr=25

        local xend=44

        local grp="females"


        set maxiter 50

        set more off

        tempfile tempf tempg

        local dir1 "P:\Evan\Informatics\North-South divide\specific-causes\"

        local dir2 "`dir1'\graphs\"

        cd "`dir2'"

        //strings and matrices

        local midagelst="0.5 3 7.5 12.5 17.5 22.5 27.5 32.5 37.5 42.5 47.5 52.5 57.5 62.5 67.5
72.5 77.5 82.5 87.5"
```

```
matrix mat1 = J(19,5,.)

matrix colnames mat1 = IRR SE EXD deathsN midage

//load data

qui use "`dir1'dataset.dta",clear

qui keep if inlist(year,2014,2015,2016)

label define nslbl 0 "South" 1 "North"

label val north nslbl

//keep data of interest

if "`grp'"=="males" {

	qui keep if male==1

}

if "`grp'"=="females" {

	qui keep if male==0

}

qui collapse (sum) deaths0, by(ageband agecat midage north population)

qui save `tempg', replace


//open log

qui log using "`dir1'log6b-IRR deaths `xstr'-`xend' `grp'", name(log1) smcl replace


poisson deaths0 north if midage>=`xstr' & midage<=`xend', irr exposure(population)



poisson deaths0 north if midage==27.5, irr exposure(population)

poisson deaths0 north if midage==32.5, irr exposure(population)

poisson deaths0 north if midage==37.5, irr exposure(population)
```

poisson deaths0 north if midage==42.5, irr exposure(population)

//log close

qui log close _all

qui log2html "`dir1'log6b-IRR deaths `xstr'-`xend' `grp'", replace ti("Poisson (IRR) based death count model for 2014-2016: `xstr'-`xend', `grp'")

end

## 07- Get data LSOA

local dir1 "P:/Evan/Informatics/North-South divide/specific-causes/"

local dir2 "`dir1'/original_data/"

local dir3 "R:/Evan/Databases/2011LSOA-population/"

local dir4 "R:/Evan/Databases/2011LSOA-PCO-SHA/"

local dir5 "R:/Evan/Databases/IMD/2011LSOA/"

cd "`dir1'"

tempfile tempe tempf

set more off

//2016 deaths excel at the LSOA level

import excel "`dir2'deathsbylsoanorthsouthdivideengland2016.xlsx", sheet("Table") firstrow cellrange(A4:AO32771) clear

drop V

replace Ind="North" if Ind=="N"

replace Ind="South" if Ind=="S"

encode Ind, gen(region)

drop Ind

rename LSOAarea lsoacode2011

```
//age groups
local cnt=-1
foreach x of varlist C-U {
	local cnt=`cnt'+1
	rename `x' males`cnt'
}
local cnt=-1
foreach x of varlist W-AO {
	local cnt=`cnt'+1
	rename `x' females`cnt'
}
foreach x in males females {
	qui replace `x'1=`x'1+`x'0
	drop `x'0
	label var `x'1 "<=04"
}
//reshape
reshape long males females, i(lsoacode2011) j(agegrp)
rename males deaths1
rename females deaths0
reshape long deaths, i(lsoacode2011 agegrp) j(sex)
qui save `tempe', replace

//add population data
local strm "males"
local strf "females"
```

```
foreach z in m f {
	qui use "`dir3'/lsoa2011_`str`z''_by_year.dta", clear
	qui keep if year==2016
	keep lsoacode2011 `z'0to4-`z'90plus
	local cnt=0
	foreach x of varlist `z'0to4-`z'90plus {
		local cnt=`cnt'+1
		rename `x' `str`z''`cnt'
	}
	qui replace `str`z''18=`str`z''18+`str`z''19
	qui drop `str`z''19
	capture save `tempf'
	if _rc!=0 {
		qui merge 1:1 lsoacode2011 using `tempf', nogen
	}
}
//reshape
reshape long males females, i(lsoacode2011) j(agegrp)
rename males population1
rename females population0
reshape long population, i(lsoacode2011 agegrp) j(sex)
merge 1:1 lsoacode2011 agegrp sex using `tempe', nogen
//there are LSOAs that do not appear in the deaths file =>assume all zeros are ommitted
qui gen midage=2
forvalues i=2(1)18 {
	qui replace midage=(`i'-1)*5+2.5 if agegrp==`i'
```

```
}
qui replace deaths=0 if deaths==.
//labels and such
label var midage "Midage"
label var region "Region"
label define sexlbl 0 "Females" 1 "Males"
label val sex sexlbl
label var sex "Sex"
label define agelbl 1 "<=04" 2 "05-09" 3 "10-14" 4 "15-19" 5 "20-24" 6" 25-29" 7 "30-34" ///
8 "35-39" 9 "40-44" 10 "45-49" 11 "50-54" 12 "55-59" 13 "60-64" 14 "65-69" 15 "70-74" ///
16 "75-79" 17 "80-84" 18 "85+"
label val agegrp agelbl
label var agegrp "Age group"
label var deaths "Deaths"
label var population "Population"
qui save `tempe', replace

//add North South info for LSOAs with no death info
local dir4 "R:/Evan/Databases/2011LSOA-PCO-SHA/"
qui use "`dir4'/oa11_to_lsoa11.dta", clear
qui keep oa11cd lsoa11cd
qui merge 1:1 oa11cd using "`dir4'/oa11_to_PCO-SHA.dta"
qui keep if _merge==3
qui keep lsoa11cd sha11cd sha11nm
rename lsoa11cd lsoacode2011
qui duplicates drop lsoacode2011, force
```

qui merge 1:m lsoacode2011 using `tempe'

qui keep if _merge==3 /*Wales in the population files*/

qui replace region=1 if inlist(sha11cd,"E18000001","E18000002","E18000003","E18000004","E18000005") /*North*/

qui replace region=2 if inlist(sha11cd,"E18000006","E18000007","E18000008","E18000009","E18000010") /*South*/

qui drop _merge

label var sha11cd "SHA code"

label var sha11nm "SHA name"

label var lsoacode2011 "LSOA code 2011"

qui save `tempe', replace

//add 2015 deprivation

qui use "`dir5'/imd2015_LSOA11.dta", clear

*qui keep lsoacode2011 imd2015

merge 1:m lsoacode2011 using `tempe', nogen

//save

compress

order region lsoacode2011 sha11cd sha11nm sex agegrp midage deaths population imd*

sort lsoacode2011 sex agegrp

save "datasetLSOA.dta", replace

## 08- Poisson LSOA IMD aggr

//run Poisson models at the LSOA level

```
capture program drop getPm

program getPm

	set more off

	local dir1 "P:/Evan/Informatics/North-South divide/specific-causes/"

	use "datasetLSOA.dta", clear

	xi i.midage

	qui encode sha11nm, gen(sha2011)


	//log open

	qui log using "log8a-Poisson models", name(log1) smcl replace


	di _newline(3) as text "Not including deprivation"

	//region=N/S model and Poisson

	poisson deaths ib2.region i.sex _Imidage_2-_Imidage_18 if population>0, 
exposure(population) irr


	di _newline(3) as text "Full model: deprivation and N/S binary"

	//region=N/S model and Poisson

	poisson deaths ib2.region i.sex imd2015 _Imidage_2-_Imidage_18 if population>0, 
exposure(population) irr


	di _newline(3) as text "Full model: deprivation and region"

	//region=N/S model and Poisson

	poisson deaths i.sex imd2015 ib3.sha2011 _Imidage_2-_Imidage_18 if population>0, 
exposure(population) irr


	//log close
```

```
	qui log close _all
	qui log2html "log8a-Poisson models", replace ti("Poisson models at the LSOA level")

end


//run negative binomial models at the LSOA level
capture program drop getNBm
program getNBm
	set more off
	local dir1 "P:/Evan/Informatics/North-South divide/specific-causes/"
	use "datasetLSOA.dta", clear
	xi i.midage
	qui encode sha11nm, gen(sha2011)

	//log open
	qui log using "log8b-Negative binomial models", name(log1) smcl replace

	di _newline(3) as text "Not including deprivation"
	//region=N/S model and negative binomial regression
	nbreg deaths ib2.region i.sex _Imidage_2-_Imidage_18 if population>0,
exposure(population) irr

	di _newline(3) as text "Full model: deprivation and N/S binary"
	//region=N/S model and negative binomial regression
	nbreg deaths ib2.region i.sex imd2015 _Imidage_2-_Imidage_18 if population>0,
exposure(population) irr
```

di _newline(3) as text "Full model: deprivation and region"

//region=N/S model and negative binomial regression

nbreg deaths i.sex imd2015 ib3.sha2011 _Imidage_2-_Imidage_18 if population>0, exposure(population) irr

//log close

qui log close _all

qui log2html "log8b-Negative binomial models", replace ti("NB models at the LSOA level")

end

## 09- Poisson LSOA IMD ind all

//run Poisson models at the LSOA level

capture program drop getPm

program getPm

set more off

local dir1 "P:/Evan/Informatics/North-South divide/specific-causes/"

use "datasetLSOA.dta", clear

qui xi i.midage

qui encode sha11nm, gen(sha2011)

//log open

qui log using "log9a-Poisson models", name(log1) smcl replace

//collinearity issues so dropping some domains

collin region sex imdB2015 imdE2015 imdF2015 imdG2015 _Imidage_2-_Imidage_18 if population>0

```
	di _newline(3) as text "Full model: individual deprivation domains (excl income, education and health) and N/S binary"

	//region=N/S model and Poisson

	poisson deaths ib2.region i.sex imdB2015 imdE2015 imdF2015 imdG2015 _Imidage_2-_Imidage_18 if population>0, exposure(population) irr


	//collinearity issues so dropping some domains

	qui xi i.sha2011 i.midage

	collin sex imdB2015 imdE2015 imdF2015 imdG2015 _Isha2011_2-_Isha2011_10 _Imidage_2-_Imidage_18 if population>0

	di _newline(3) as text "Full model: individual deprivation domains (excl income, education and health) and region"

	//region=N/S model and Poisson

	poisson deaths i.sex imdB2015 imdE2015 imdF2015 imdG2015 ib3.sha2011 _Imidage_2-_Imidage_18 if population>0, exposure(population) irr


	//log close

	qui log close _all

	qui log2html "log9a-Poisson models", replace ti("Poisson models at the LSOA level (individual IMD domains excl income, education and health)")

end


//run negative binomial models at the LSOA level

capture program drop getNBm

program getNBm

	set more off

	local dir1 "P:/Evan/Informatics/North-South divide/specific-causes/"

	use "datasetLSOA.dta", clear
```

qui xi i.midage

qui encode sha11nm, gen(sha2011)

//log open

qui log using "log9b-Negative binomial models", name(log1) smcl replace

di _newline(3) as text "Full model: individual deprivation domains (excl income, education and health) and N/S binary"

//region=N/S model and negative binomial regression

nbreg deaths ib2.region i.sex imdB2015 imdE2015 imdF2015 imdG2015 _Imidage_2-_Imidage_18 if population>0, exposure(population) irr

di _newline(3) as text "Full model: individual deprivation domains (excl income, education and health) and region"

//region=N/S model and negative binomial regression

nbreg deaths i.sex imdB2015 imdE2015 imdF2015 imdG2015 _Imidage_2-_Imidage_18 if population>0, exposure(population) irr

//log close

qui log close _all

qui log2html "log9b-Negative binomial models", replace ti("NB models at the LSOA level (individual IMD domains excl income, education and health)")

end

## 10- Region plots

capture program drop regplot

program regplot

set more off

```
local dir1 "P:\Evan\Informatics\North-South divide\specific-causes\"

local dir2 "`dir1'\graphs\"

capture mkdir "`dir2'"

cd "`dir2'"

tempfile tempf

qui use "`dir1'dataset_regionres.dta", clear

foreach x of varlist IRR IRR_lo IRR_up {

        qui replace `x'=(`x'-1)*100

}

qui save `tempf', replace


//loop for 2 model results

foreach mdl in 3 5 {

        qui use `tempf', clear

        qui keep if model==`mdl'

        //sort on IRR

        gsort -IRR

        qui egen newreg=seq()

        label define nreglbl 1 "`=region_str[1]'" 2 "`=region_str[2]'" 3
"`=region_str[3]'" 4 "`=region_str[4]'" ///

        5 "`=region_str[5]'" 6 "`=region_str[6]'" 7 "`=region_str[7]'" 8
"`=region_str[8]'" 9 "`=region_str[9]'"

        label val newreg nreglbl

        //plot

        graph twoway rcap IRR_up IRR_lo newreg || scatter IRR newreg if area==0,
ms(O) mc(forest_green) ///

                || scatter IRR newreg if area==1, ms(D) mc(maroon) ///
```

```
			legend(order(2 3 1)) ///
			legend(rows(1) label(2 "South") label(3 "North") label(1 "95% CI")) ///
			ytitle("% adjusted excess mortality vs London", size(2.5)) ///
			xtitle("Region") ///
			yscale(range(0)) ///
			ylabel(#5, format(%3.0f) labsize(2.5) angle(horizontal)) ///
			xlabel(1(1)9, valuelabel labsize(2.2) angle(45)) ///
			xsize(6) ysize(4)

		graph export "10-regions_IRR_model`mdl'.png", replace
		graph export "10-regions_IRR_model`mdl'.eps", replace
		*graph export "10-regions_IRR_model`mdl'.pdf", replace
		graph save "10-regions_IRR_model`mdl'.gph", replace
		window manage close graph
	}

end

/*combines graphs into a single file to be used in publication - one legend*/
capture program drop combgraph
program combgraph
	//inputs
	local dir1 "P:\Evan\Informatics\North-South divide\specific-causes\"
	local dir2 "`dir1'\graphs\"
	cd "`dir2'"
	local dir3 "P:\Evan\Informatics\North-South divide\specific-causes\paper\graphs\"
```

```
   //findit grc1leg2

   grc1leg2 "10-regions_IRR_model3.gph" "10-regions_IRR_model5.gph", /*

   */ cols(1) imargin(small) legendfrom("10-regions_IRR_model3.gph") /*

         */ xtitlefrom("10-regions_IRR_model3.gph") xtob1title ring(60) lsize(2.2) ysize(8)
xsize(5.5)

   graph display, ysize(8) xsize(5.5)

   graph export "`dir3'Fig4.eps", replace

         graph export "`dir3'Fig4.png", replace width(1200)

end
```

## 11- LSOA SMR plots

```
capture program drop getall

program getall

         foreach x in people male female {

                  *smrplots 0 deaths `x'

                  smrplots 1 deaths `x'

         }

end


capture program drop smrplots

program smrplots

         //GET SMRs

         //inputs

         if "`1'"=="" {

                  di as error "Need inputs, use the wrapper or e.g. smrplots 0 deaths people"

                  error 197

         }
```

```
	local tp "`1'"
	local dvar "`2'"
	local sex "`3'"
	set more off
	local dir1 "P:/Evan/Informatics/North-South divide/specific-causes/"
	local dir2 "`dir1'/graphs/"
//auxilliary directory
	local dir3 "P:/Evan/Spatial/Large programme/data_intermediate/"
	//temp variables
	tempvar tempv

	cd "`dir2'"
	qui use "`dir1'datasetLSOA.dta", clear

	//calculate SMRs (north and south)
	qui {
		if `tp'==0 {
			qui drop if midage>=75
			local scat=1
			local ecat=15
			local tmpstr "aged{&le}75"
			local tmpstx "undr75"
		}
		else if `tp'==1 {
			qui drop if midage>44 | midage<25
			local scat=6
```

```
		local ecat=9
		local tmpstr "25{&le}aged{&le}44"
		local tmpstx "25t044"
	}
	if "`sex'"=="male" {
		keep if sex==1
	}
	else if "`sex'"=="female" {
		keep if sex==0
	}

	***
	keep `dvar' population sex agegrp region lsoacode2011 sha11cd sha11nm
	//for whole population
	if "`sex'"!="male" & "`sex'"!="female" {
		rename `dvar' `dvar'_
		rename population population_
		reshape wide `dvar'@_ population@_, i(sex lsoacode2011) j(agegrp)

		reshape wide `dvar'* population*, i(lsoacode2011) j(sex)
		//calculate mortality counts across all England, to relate to 100k
deaths (Add up to 100k)
		scalar fden=0
		scalar mden=0
		forvalues i=`scat'(1)`ecat' {
			//numerators
			qui sum population`i'_0
```

```
				scalar num`i'f=r(sum)
				qui sum population`i'_1
				scalar num`i'm=r(sum)
				//denominators
				scalar fden = fden + num`i'f
				scalar mden = mden + num`i'm
			}
			forvalues i=`scat'(1)`ecat' {
				//not rounding this time
				scalar p`i'_0=(100000*num`i'f/fden)
				scalar p`i'_1=(100000*num`i'm/mden)
			}
			//calculate SMR into variable
			gen SMR=0
			gen `tempv'=0
			foreach x in 0 1 {
				forvalues i=`scat'(1)`ecat' {
					replace
SMR=SMR+((`dvar'`i'_`x'/population`i'_`x')*p`i'_`x')/2 if population`i'_`x'>0
					replace `tempv'=`tempv'+population`i'_`x'
				}
			}
			replace SMR=. if `tempv'<100
			drop `tempv'
			label var SMR "SMR, by agegroup and sex"
		}
```

```
		//for males-females
		else {
			reshape wide `dvar'@ population@, i(sex lsoacode2011) j(agegrp)
			//calculate mortality counts across all England, to relate to 100k
deaths (Add up to 100k)
			scalar fden=0
			forvalues i=`scat'(1)`ecat' {
				//numerator
				qui sum population`i'
				scalar num`i'f=r(sum)
				//denominator
				scalar fden = fden + num`i'f
			}
			forvalues i=`scat'(1)`ecat' {
				//not rounding this time
				scalar p`i'=(100000*num`i'f/fden)
			}
			//calculate SMR into variable
			gen SMR=0
			gen `tempv'=0
			forvalues i=`scat'(1)`ecat' {
				replace SMR=SMR+((`dvar'`i'/population`i')*p`i') if
population`i'>0
				replace `tempv'=`tempv'+population`i'
			}
			replace SMR=. if `tempv'<100
			drop `tempv'
```

```
            label var SMR "SMR, by agegroup for `sex's"
        }

        qui egen allD=rowtotal(`dvar'*)
        label var allD "All deaths"
        /*calculate CI for SMR*/
        qui gen SMRlo = SMR - invnormal(.975)*SMR/sqrt(allD)
        label var SMRlo "SMR lower 95% CI"
        qui gen SMRup = SMR + invnormal(.975)*SMR/sqrt(allD)
        label var SMRup "SMR upper 95% CI"
        order region allD SMR*
        //add lsoasid2011
        merge m:1 lsoacode2011 using "`dir3'/lsoa_data.dta"
        drop if _merge!=3
        compress
    }


    //GRAPH SPATIAL
    //where county layer coordinates are
    local dir4 "P:/Evan/Spatial/Large programme/data_auxiliary/"
    //where SHA coordinates are
    local dir5 "P:/Evan/QOF/GPSystems/spatial/"
cd "`dir1'"
    tempfile tempf1 tempf2 tempx
```

```
/*use data*/

qui sort lsoacode2011

qui save `tempf1', replace


local tmpndlbl="<100 population"

/*loop for all England, Greater London, Greater Manchester,  West Midlands and SHAs*/

foreach x in England {

*foreach x in England Manchester Birmingham E18000001 E18000002 E18000003 E18000004 E18000005 E18000006 E18000007 E18000008 E18000009 E18000010 {

        qui use `tempf1', clear

        local lblstr ""

        local plgstr ""

        local outstr="`x'"

        local pngdwidth=1200

        if "`x'"=="England" {

                local plgstr "polygon(data("`dir5'sha_coordinates.dta") ocolor(black) osize(medium))"

                local lblstr "label(data("`dir4'Labels_SHA.dta") x(E_centroid) y(N_centroid) label(sha11nm) size(*0.6 ..) length(20)  color(black))"

                local lsoaborder "ocolor(none ..) ndocolor(none ..)"


                local pos=9

                local pngwidth=2400

        }

        /*E01004766 to E01005930*/

        else if "`x'"=="Manchester" {
```

```
		qui keep if strpos((lsoaname2011),"Wigan")>0 | strpos((lsoaname2011),"Bolton")>0 | strpos((lsoaname2011),"Bury")>0 /*
		*/ | strpos((lsoaname2011),"Rochdale")>0 | strpos((lsoaname2011),"Oldham")>0 | strpos((lsoaname2011),"Manchester")>0 /*
		*/ | strpos((lsoaname2011),"Salford")>0 | strpos((lsoaname2011),"Trafford")>0 | strpos((lsoaname2011),"Tameside")>0 /*
		*/ | strpos((lsoaname2011),"Stockport")>0
			local plgstr "polygon(data("`dir4'GreaterManchester_coordinates.dta") ocolor(black) osize(medium))"
			*local lblstr "label(data("Labels_Manchester.dta") x(E_centroid) y(N_centroid) label(lsoalabel) size(*1.3 ..) color(purple))"
			*local lsoaborder "osize(vvthin ..)"
			local lsoaborder "ocolor(none ..) ndocolor(none ..)"
			local pos=7
		}
		/*E01008881 to E01010567*/
		else if "`x'"=="Birmingham" {
			qui keep if strpos((lsoaname2011),"Wolverhampton")>0 | strpos((lsoaname2011),"Walsall")>0 | strpos((lsoaname2011),"Dudley")>0 /*
			*/ | strpos((lsoaname2011),"Sandwell")>0 | strpos((lsoaname2011),"Birmingham")>0 | strpos((lsoaname2011),"Solihull")>0 /*
			*/ | strpos((lsoaname2011),"Coventry")>0
			local outstr="`x'"
			local plgstr "polygon(data("`dir4'WestMidlands_coordinates.dta") ocolor(black) osize(medium))"
			*local lblstr "label(data("Labels_Birmingham.dta") x(E_centroid) y(N_centroid) label(lsoalabel) size(*1.3 ..) color(purple))"
			*local lsoaborder "osize(vvthin ..)"
			local lsoaborder "ocolor(none ..) ndocolor(none ..)"
```

```
			local pos=2
		}
		/*Wales*/
		else if "`x'"=="Wales" {
			/*id different to code but same order*/
			qui keep if country==1
			local outstr="Wales"
			local plgstr "polygon(data("`dir4'Wales_coordinates.dta") ocolor(black) osize(medium))"
			local lsoaborder "ocolor(none ..) ndocolor(none ..)"
			local pos=9
		}
		//SHAs
		else {
			qui keep if sha11cd=="`x'"
			local outstr=sha11nm[1]
			local plgstr "polygon(data("`dir4'SHA-`x'_coordinates.dta") ocolor(black) osize(medium))"
			local lsoaborder "ocolor(none ..) ndocolor(none ..)"
			local pE18000001=3
			local pE18000002=9
			local pE18000003=7
			local pE18000004=7
			local pE18000005=1
			local pE18000006=5
			local pE18000007=7
			local pE18000008=5
```

```
local pE18000009=5

local pE18000010=10

local pos=`p`x''

}


//custom breaks

local cb0people "0 100 200 300 400 500 600 3504"

local cb0male "0 100 200 300 400 500 600 700 800 6736"

local cb0female "0 100 200 300 400 500 600 700 800 6152"

local cb1people "0 0.001 100 200 300 400 500 600 2460"

local cb1male "0 0.001 100 200 300 400 500 600 3514"

local cb1female "0 0.001 100 200 300 400 500 600 2863"

*clmethod(custom) clbreaks(`cb`tp'`sex'')

*clmethod(quantile) clnumber(9)

local strcol="Purples"

if `tp'==1 local strcol="Reds"


/*actual graph*/

format SMR %4.0f

local ttlstr=""

spmap SMR using "`dir3'lsoa_coordinates.dta", id(lsoasid2011) legend(region(lcolor(mint)) position(`pos')) fcolor(`strcol') ndfcolor(yellow) ndlabel("`tmpndlbl'") /*

*/ legstyle(1) clmethod(custom) clbreaks(`cb`tp'`sex'') `plgstr' `lblstr' `lsoaborder' title("`ttlstr'", size(medium))

local tmpprfx "SMR"

graph export "`dir2'/11-`tmpprfx'_`outstr'_`tmpstx'_`sex'.png", replace width(`pngwidth')
```

```
		if "`x'"=="England" & "`sex'"=="people" {
			graph export "`dir2'/11`tmpprfx'_`outstr'_`tmpstx'_`sex'.pdf", replace

		}
	}

End
```

## 12- Cross-sectional IRR age aggr

```
capture program drop getall
program getall
	irrplot 25 44 all 20
	*irrplot 25 44 males 20
	*irrplot 25 44 females 20
	*irrplot 0 75 all 20
	*irrplot 0 75 males 20
	*irrplot 0 75 females 20
	//combine graphs
	*combgraph 25 44
end

capture program drop irrplot
program irrplot
	//inputs
	local xstr=`1'
	local xend=`2'
	local grp="`3'"
```

```
/*threshold of total deaths within year above which models are run*/
local lmt `4'


***
set maxiter 50
set more off
tempfile tempf tempg
local dir1 "P:\Evan\Informatics\North-South divide\specific-causes\"
local dir2 "`dir1'\graphs\"
cd "`dir2'"
//strings and matrices
local midagelst="0.5 3 7.5 12.5 17.5 22.5 27.5 32.5 37.5 42.5 47.5 52.5 57.5 62.5 67.5 72.5 77.5 82.5 87.5"
matrix mat1 = J(1,2,.)
matrix colnames mat1 = IRR SE
//load data
qui use "`dir1'dataset.dta",clear
qui keep if inlist(year,2014,2015,2016)
label define nslbl 0 "South" 1 "North"
label val north nslbl
//keep data of interest
if "`grp'"=="males" {
	qui keep if male==1
}
if "`grp'"=="females" {
	qui keep if male==0
```

```
}
qui collapse (sum) deaths0-deaths8, by(ageband agecat midage north population)
qui save `tempg', replace

//loop on death group
forvalues i=1(1)8 {
	qui use `tempg', clear
	local dvar "deaths`i'"
	qui sum `dvar' if midage>=`xstr' & midage<=`xend'
	if r(sum) > `lmt' {
		qui poisson `dvar' north if midage>=`xstr' & midage<=`xend',
exposure(population)
		matrix mat1[1,1]=exp(_b[north])
		matrix mat1[1,2]=exp(_b[north])*_se[north]
	}
	else {
		matrix mat1[1,1]=1
		matrix mat1[1,2]=.
	}

	drop _all
	qui svmat mat1, names(col)
	qui gen deaths=`i'
	capture save `tempf'
	if _rc!=0 {
		append using `tempf'
```

```
			qui save `tempf', replace
		}
	}
	qui use `tempf', clear

	//make some edits and save as dataset to report in appendix
	qui gen IRR_lo=IRR-invnormal(0.975)*SE
	qui gen IRR_up=IRR+invnormal(0.975)*SE
	qui gen north=1
	label define nslbl 0 "South" 1 "North"
	label val north nslbl
	label var IRR "Incidence rate ratio"
	label define dthlbl 1 "Accidents" 2 "Alcohol" 3 "Cardiovascular" 4 "Suicide" ///
	5 "Drugs" 6 "Cancer (excl breast)" 7 "Breast cancer" 8 "Other"
	label var deaths "Death type"
	label val deaths dthlbl
	qui compress
	sort deaths
	order deaths IRR*
	qui save "`dir1'IRR aggregate `xstr'-`xend' `grp'.dta", replace

end
```
